# Well-differentiated G1 and G2 pancreatic neuroendocrine tumors: a meta-analysis of published expanded DNA sequencing data

**DOI:** 10.3389/fendo.2024.1351624

**Published:** 2024-05-29

**Authors:** Kirstine Øster Andersen, Sönke Detlefsen, Klaus Brusgaard, Henrik Thybo Christesen

**Affiliations:** ^1^ Hans Christian Andersen Children’s Hospital, Odense University Hospital, Odense, Denmark; ^2^ Department of Clinical Research, Faculty of Health Sciences, University of Southern Denmark, Odense, Denmark; ^3^ Odense Pancreas Center (OPAC), Odense, Denmark; ^4^ Department of Pathology, Odense University Hospital, Odense, Denmark; ^5^ Department of Clinical Genetics, Odense University Hospital, Odense, Denmark; ^6^ Steno Diabetes Center Odense, Odense, Denmark

**Keywords:** pancreatic neuroendocrine tumors, systematic review, meta-analysis, genetics, somatic, germline, MEN1, Knudson’s two-hit hypothesis

## Abstract

**Introduction:**

Well-differentiated pancreatic neuroendocrine tumors (PNETs) can be non-functional or functional, e.g. insulinoma and glucagonoma. The majority of PNETs are sporadic, but PNETs also occur in hereditary syndromes, primarily multiple endocrine neoplasia type 1 (MEN1). The Knudson hypothesis stated a second, somatic hit in *MEN1* as the cause of PNETs of MEN1 syndrome. In the recent years, reports on genetic somatic events in both sporadic and hereditary PNETs have emerged, providing a basis for a more detailed molecular understanding of the pathophysiology. In this systematic review and meta-analysis, we made a collation and statistical analysis of aggregated frequent genetic alterations and potential driver events in human grade G1/G2 PNETs.

**Methods:**

A systematic search was performed in concordance with the Preferred Reporting Items for Systematic Review and Meta-Analyses (PRISMA) reporting guidelines of 2020. A search in Pubmed for published studies using whole exome, whole genome, or targeted gene panel (+400 genes) sequencing of human G1/G2 PNETs was conducted at the 25^th^ of September 2023. Fourteen datasets from published studies were included with data on 221 patients and 225 G1/G2 PNETs, which were divided into sporadic tumors, and hereditary tumors with pre-disposing germline variants, and tumors with unknown germline status. Further, non-functioning and functioning PNETs were distinguished into two groups for pathway evaluation. The collated genetical analyses were conducted using the ‘maftools’ R-package.

**Results:**

Sporadic PNETs accounted 72.0% (162/225), hereditary PNETs 13.3% (30/225), unknown germline status 14.7% (33/225). The most frequently altered gene was *MEN1*, with somatic variants and copy number variations in overall 42% (95/225); hereditary PNETs (germline variations in *MEN1*, *VHL*, *CHEK2*, *BRCA2*, *PTEN*, *CDKN1B*, and/or *MUTYH*) 57% (16/30); sporadic PNETs 36% (58/162); unknown germline status 64% (21/33). The *MEN1* point mutations/indels were distributed throughout *MEN1*. Overall, *DAXX* (16%, 37/225) and *ATRX*-variants (12%, 27/225) were also abundant with missense mutations clustered in mutational hotspots associated with histone binding, and translocase activity, respectively. *DAXX* mutations occurred more frequently in PNETs with *MEN1* mutations, p<0.05. While functioning PNETs shared few variated genes, non-functioning PNETs had more recurrent variations in genes associated with the Phosphoinositide 3-kinase, Wnt, NOTCH, and Receptor Tyrosine Kinase-Ras signaling onco-pathways.

**Discussion:**

The somatic genetic alterations in G1/G2 PNETs are diverse, but with distinct differences between sporadic vs. hereditary, and functional vs. non-functional PNETs. Increased understanding of the genetic alterations may lead to identification of more drivers and driver hotspots in the tumorigenesis in well-differentiated PNETs, potentially giving a basis for the identification of new drug targets. (Funded by Novo Nordisk Foundation, grant number NNF19OC0057915).

## 1 Introduction

Pancreatic neuroendocrine tumors (PNETs) represent a rare tumor type accounting for less than 3% of all pancreatic malignancies (1). Most PNETs develop sporadically. Here as only five to ten percent of PNETs occur due to hereditary syndromes including multiple endocrine neoplasia type 1 (MEN1), and more rarely von Hippel-Lindau disease, neurofibromatosis type 1, and tuberous sclerosis (2).

According to the Knudson’s two-hit hypothesis from 1993, PNETs occurring in patients with MEN1 syndrome are likely caused by a somatic second hit in *MEN1* in the PNET in individuals carrying a germline *MEN1* mutation (3, 4). The *MEN1* gene encodes the putative tumor suppressor menin, which plays a role in biological processes such as histone and transcription regulation (5–9), DNA repair (10), and apoptosis (9).

Somatic variants in *MEN1* gene are reported in 25–44% of all PNETs (11–14). Additionally, *DAXX* and *ATRX*, both encoding chromatin-remodelers, are frequently altered somatically in PNETs, as well as other genes in the mTOR and DNA repair pathways (12, 13). PNETs may be non-functioning, or functioning leading to a clinically measurable hormonal hypersecretion syndrome such as insulinoma and glucagonoma. A mutational hotspot in *YY1*, (p.Thr372Arg), has been identified in 8–30% of sporadic insulinomas (15–17). Along with *YY1*, other genes have been proposed to be drivers in insulinomas (18, 19), although their relative frequencies are rather low.

In the last decades, investigations with expanded genetic analyses have provided an explosion of our knowledge on somatic gene changes in tumors in general, which may contribute to clinical diagnosis, prognosis, treatment and categorization of patients. Little is known, however, about tumor genetics by expanded genetic analyses in well differentiated (wd)-PNETs.

Understanding the tumor genetics of PNETs may provide a basis for improved diagnosis and management for PNET patients and may contribute to the discovery of new drug targets as a supplement to present drugs, such as the mTOR-inhibitor everolimus and the kinase inhibitor sunitinib malate (20–22). Given these perspectives, we aimed to conduct a systematic review of published DNA sequencing data gained by expanded genetic analyses of wd-PNETs following the Preferred Reporting Items for Systematic Review and Meta-Analyses (PRISMA) reporting guidelines (23). Thereby we gathered information to shed light on the frequently altered genes in association to genetically predisposed patients and distinct functionality of PNETs.

## 2 Methods

### 2.1 Search string

Genetic datasets of PNETs using whole exome sequencing (WES), whole genome sequencing (WGS), and targeted gene-panels of more than 400 genes were searched for in Pubmed on 25^th^ of September 2023 using the following search criteria: “Pancreatic neuroendocrine tumor” AND (“sequencing” OR “NGS”), “Pancreatic neuroendocrine neoplasm” AND (“sequencing” OR “NGS”),”Insulinoma” AND (“sequencing” OR “NGS”), “Glucagonoma” AND (“sequencing” OR “NGS”),”Somatostatinoma” AND (“sequencing” OR “NGS),”Gastrinoma” AND (“sequencing” OR “NGS”),”VIPoma” AND (“sequencing” OR “NGS”), “Serotonin-producing tumors” AND (“sequencing” OR “NGS”), “ACTH-producing tumors” AND (“sequencing” OR “NGS”), “Gastroenteropancreatic” AND (“sequencing” OR “NGS”), “GEP-NET” AND (“sequencing” OR “NGS”), “GEPNET” AND (“sequencing” OR “NGS”), “PNET” AND (“sequencing” OR “NGS”), “P-NET” AND (“sequencing” OR “NGS”), “PanNET” AND (“sequencing” OR “NGS”), “Pan-NET” AND (“sequencing” OR “NGS”), “PNEN” AND (“sequencing” OR “NGS”). The studies were screened by one reviewer and included datasets were also retrieved by the same reviewer.

All pancreatic functional tumor types in the search were retrieved from Guilmette et al (2019) (24) and WHO Classification of Tumors of Endocrine Organs (2017) (25). The search included words appearing in the title and abstract of each article. Selected articles were then screened individually for inclusion and exclusion criteria as stated below.

### 2.2 Inclusion and exclusion criteria

We included studies using WES, WGS and/or a gene panel of more than 400 genes to determine the somatic variants in PNETs. The cut-off of 400 genes was chosen based on a Japanese study with use of data from The Cancer Genome Atlas (26), showing that cancer-panel sizes at or above 400 genes had the best statistic performance power to identify tissue mutations in comparison to WES (26). In our dataset, we included human PNETs with tumor grade G1 or G2. Wd-G3 PNETs were not included in the present analyses given the interpretation difficulties and the fact that G3-NETs were not separated from G3 neuroendocrine carcinomas (NECs) in articles based on WHO classifications prior to 2017 (13). Both patients with and without tumor syndromes with both functioning and non-functioning PNETs were included. Both studies using formalin-fixed paraffin-embedded (FFPE) and fresh frozen samples were included.

**Figure 1:**
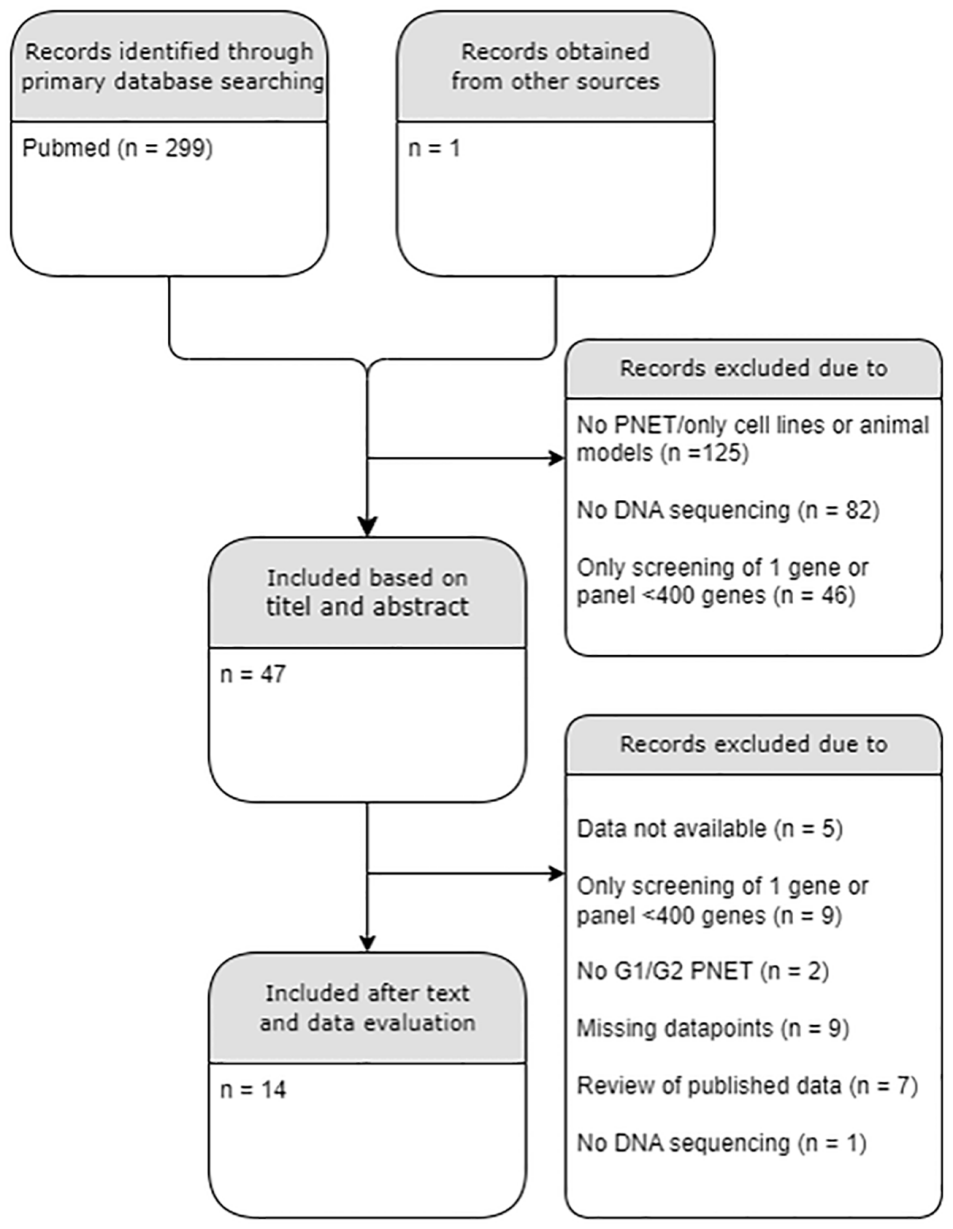
PRISMA diagram of the literature search. A total of 14/300 datasets were included. PNET, Pancreatic neuroendocrine tumor; G, histological grade. .

Exclusion criteria included studies on cell-lines and human studies on extra-pancreatic tissues, carcinomas, non-islet PNETs, metastatic tissues, PNETs with tumor grade G3 only and NECs; Studies with data without proper sample characteristics were excluded. Studies without obtainable full text or main dataset were also excluded. Replicated data were only presented once from the original study. The literature search flow chart is presented in **Figure 1**. The full list of excluded and included papers is listed in **Supplementary Tables 1**, **2** and **Table 1**, respectively.

**Table 1:** The sequencing characteristics of all 225 pancreatic neuroendocrine tumors from each of the 14 included articles.

Published article	No. of PNETs meeting the criteria	Sequencing type and platform	Average read depth (tumor)	Reference genome	No. of PNETs in CNV analysis	References
Scarpa et al. (13)	93	WGS on HiSeq 2000 (Illumina)	61	GRCh37	12‡	(13)
Cao et al. (15)	10	WES on HiSeq 2000 (Illumina)	157	GRCh37	0	(15)
Qi et al. (27)	3	WES on HiSeq 2500 (Illumina)	NA	GRCh37	0	(27)
Wang et al. (19)	16	WES on HiSeq 2500 (Illumina)	105	GRCh37	16†	(19)
Naruoka et al. (28)	2	WES on Ion Torrent platform	NA	GRCh37	2	(28)
Naruoka et al. (29)	1	WGS on HiSeq 4000 (Illumina)	NA	GRCh37	1	(29)
Tamura et al. (30)	1	WES on HiSeq 2500 (Illumina)	129	GRCh37	1	(30)
Zheng et al. (14)	12	612 cancer-related genes on NextSeq 500 (Illumina)	NA	GRCh37	0	(14)
Wang et al. (31)	2	WES on HiSeq X10 (Illumina)	400	GRCh37	2	(31)
Yachida et al. (32)*	33	WES and WGS on HiSeq 2500 (Illumina)	579.3	GRCh37	33	(32)
Melone et al. (33)**	13	523 cancer-relevant genes on NextSeq500 (Illumina)	NA	GRCh38	6	(33)
Tirosh et al. (34)	6	Panel of 500 genes on HiSeq 2000 (Illumina)	257	GRCh37	2	(34)
Yang et al. (35)	31	WES on HiSeq2500 (Illumina)	NA	GRCh37	0	(35)
Hu et al. (36)	2	WES on BGISEQ-500 (Beijing Genomics Institute)	136.18	GRCh37	0	(36)

The number of pancreatic neuroendocrine tumors from each published data set along with the sequencing type and platform was retrieved from each article. Further, the average read depth and reference genome were recovered in most cases. PNET, Pancreatic neuroendocrine tumor; WGS, Whole genome sequencing; WES, Whole exome sequencing; FFPE, Formalin fixed, paraffin-embedded; NA, Not available; CNV, copy number variation; (*) Human Genome Variation Society (HGVS) nomenclature for protein alterations were generated using the (CDS) position and the reference/tumor alleles. (**) Conversion to reference genome GrCh37 using LiftOver was performed and HGVS protein alterations were generated using the coding sequence (CDS) position and the reference/tumor alleles. (‡) Only PNETs and genes (*MEN1, VHL, CDKN1B, MUTYH, CHEK2, BRCA2*) associated with predisposition to PNET development were available for CNV analysis. (†) Only three genes available for CNV analysis (*MEN1, CDKN1C and EZH2*).

### 2.3 Meta-analysis pipeline

The meta-analysis of included data involved the following steps: 1) Select all datasets in each published database comprising samples belonging to the chosen conditions. 2) For each separate dataset, find and select PNETs with a tumor grade of G1 or G2. 3) Categorize the samples based on tumor type (functional vs non-functional) and sporadic vs hereditary when specified, retrieve DNA variations, including mutations, and copy number variations (CNVs; including amplifications (Amp), deletions (Del), and copy-neutral loss of heterozygosity (cnLOH) of gene region or whole chromosome), where applicable, 5) exclude synonymous variants, 6) exclude intronic variants, 7) exclude common variants identified in the general population (GnomAD, frequency < 1%), 8) list the mutations and CNVs in genes somatically altered in PNETs, 9) compose data to a single mutation annotation format MAF-like file for analysis as described below.

### 2.4 Data correction

One dataset was aligned to the reference genome GRCh38 and was converted to GRCh37 using LiftOver (https://genome.ucsc.edu/cgi-bin/hgLiftOver). Twelve intronic variants in *CUX* could not be converted. Alterations on nucleotide level were predicted using the software Transvar (37) using the amino acid alteration and coding sequence (CDS) as input. The generated nucleotide alteration (reference and alternative allele) was compared to four of the included datasets (14, 15, 19, 32), n=71/225. By using the software, both strands could be considered altered, thus we accepted the generated nucleotide if it followed the base pairing rule in comparison to the datasets. Two studies lacked HGVS description of amino acid alterations (32, 33), and these were generated using the genomic coordinates of the CDS and the represented amino acids (reference and alternative amino acid) or nucleotide change, respectively. The study of Scarpa et al. (13) used a different annotation when describing indels, and the HGVS annotation was achieved using Transvar based on the genomic coordinates of the CDS and the chromosome number. A few splice-variants were excluded due to missing data.

### 2.5 Mutation annotation format-file generation

Data were collected from each included article and the data were listed in a MAF-like structure. The collection of data was achieved in excel and the data-frame was compiled to a CSV-file before the generation of the MAF-file in R. The resultant MAF-like file was generated for all samples and consisted of the following information: Genetic data, including Human Genome Organization (HUGO) gene nomenclature symbol, variant classification, variant type, reference allele, tumor seq allele1, tumor seq allele2, and amino acid change along with the tumor sample barcode and the respective article. Clinical data about metastasis, tumor type, and tumor syndrome was also added to the MAF file for each tumor sample barcode. When applicable, CNV data were collected and added to an additional customized table in a second file. The file could be merged to the MAF-like file during the meta-analysis.

### 2.6 Analysis of sequencing data

The generated MAF-like file including all retrieved sample information was used for further analysis. We categorized each PNET of a patient into sporadic vs. hereditary, including germline variations in *MEN1*, *VHL*, *NF1*, *CDKN1B*, *BRCA2*, *MUTYH*, *CHEK2*, *PTEN*, by use of the descriptions from the articles and corresponding datasets. Further, each PNET was categorized into non-functional (e.g. PPomas, and other PNETs without hypersecreting syndrome) vs. functional PNETs (e.g. insulinoma, glucagonoma, VIPoma, gastrinoma) according to the retrieved published data. Further, the presence of distant metastases was registered, if published.

The R-package Maftools (38) was used to produce a summary of the file, oncoplots and a plot of implicated oncogenic pathways, along with analyses of gene-specific mutations, co-occurrence of mutations, and variant enrichment.

The pathogenicity of missense mutations in *DAXX* and *ATRX* were assessed using Polyphen-2 (39). Protein domains in DAXX and ATRX were retrieved from Wang et al. (40).

### 2.7 Statistics

P-values were determined using two-tailed Fishers-exact test, a Freeman-Halton extended Fishers exact test, or Mann-Whitney U test where appropriate. P-values less than 0.05 were considered significant. Data were analyzed using R (v. 4.2.3.).

## 3 Results

### 3.1 Clinical data

Our study included 14 of 300 datasets available using our search criteria, **Table 1**. The 14 datasets comprised a total of 225 individual G1/G2 PNET samples from 221 patients. Of the 225 PNETs included, 162 (72%) were sporadic with no associated germline alteration and 30 (13%) were hereditary with germline predisposing alterations in *MEN1* (14 PNETs in 11 patients), *MUTYH*, *CHEK2*, *VHL*, *CDKN1B*, *BRCA2*, and *PTEN*, **Table 2** and **Supplementary Table 3**. Two patients harbored variants in two predisposing genes (*MEN1*/*MUTYH* and *CHEK2*/*MUTYH*). In 33 PNETs (15%), information on germline predisposition was missing.

**Table 2:** Clinical features of 225 well-differentiated pancreatic neuroendocrine tumors (PNETs).

Variable	Hereditary	Sporadic *	Unspecified	p-value**
***Number of PNETs***	30	162	33	
***Germline variations*** *MEN1* *MUTYH* *VHL* *CHEK2* *CDKN1B* *BRCA* *PTEN*	14 (46.6%) 6 (20%) 5 (16.6%) 4 (13.3%) 1 (10%) 1 (10%) 1 (10%)			
Mean age (range)***	46 (20–78)	56 (17–87)	54 (27–77)	**0.005**
Females (%)***	13 (46.4%)	63 (39.9%)	23 (69.7%)	0.54
***PNET type***				0.80
*Functional, all*	5	40	0	
*Insulinoma*	3	34	0	
*Glucagonoma*	0	2	0	
*Gastrinoma*	2	0	0	
*VIPoma*	0	1	0	
*Unspecified*	0	3	0	
*Non-functioning*	17	106	33	
*Unspecified type*	8	16	0	
***Distant metastasis***				0.75
*Yes*	4	21	0	
*No*	18	120	1	
*Unspecified*	8	21	32	

PNET, Pancreatic neuroendocrine tumor; VIPoma, Vasoactive intestinal peptide tumor. *Sporadic may refer to PNETs without MEN1 germline variant. ** Hereditary vs. sporadic. *** Age was not specified for 31 individuals and sex was not specified for six individuals. These individuals were not included in the given statistical analyses.

The bold values are representing p-values < 0.05

Patients with germline predisposition were significantly younger than patients with sporadic PNETs, 46 vs. 56 years, p=0.005. The PNETs were functional in 45 (20%), of which insulinomas accounted for 37 (82%), non-functional in 156 (69%), and with unspecified functionality in 24 (11%). Our meta-analysis showed no difference in functional and non-functional PNETs in hereditary vs. sporadic PNETs, 5/22 (22.7%) vs. 40/146 (27.4%), p=0.80.

Of the 225 PNETs, 25 (11%) were accompanied with distant metastasis. No difference was seen between genetically predisposed and sporadic PNETs regarding the presence of metastasis.

### 3.2 Somatic DNA variants by Transvar vs. published data

The 225 PNETs had a total of 6,194 reported non-synonymous somatic variants. We identified a difference between computed nucleotide change by Transvar and the annotated change in 74 cases, accounting for 7.3% of tested nucleotide alterations. In addition, the Transvar software failed to identify nucleotide change for 420 of the reported changes. In 118 cases, the cause of failing was an invalid gene annotation, 294 lacked a valid transcript, 6 had an invalid mutation nomenclature and 2 were out of range. Due to the pronounced difference between the Transvar output and the published data, we decided only to use the annotations described by the articles regarding the nucleotides on each tumor allele. The overall distribution of transversions and transitions in the resultant dataset is shown in **Supplementary Figure 1**.

### 3.3 Somatic DNA variations in PNETs

**Figure 2:**
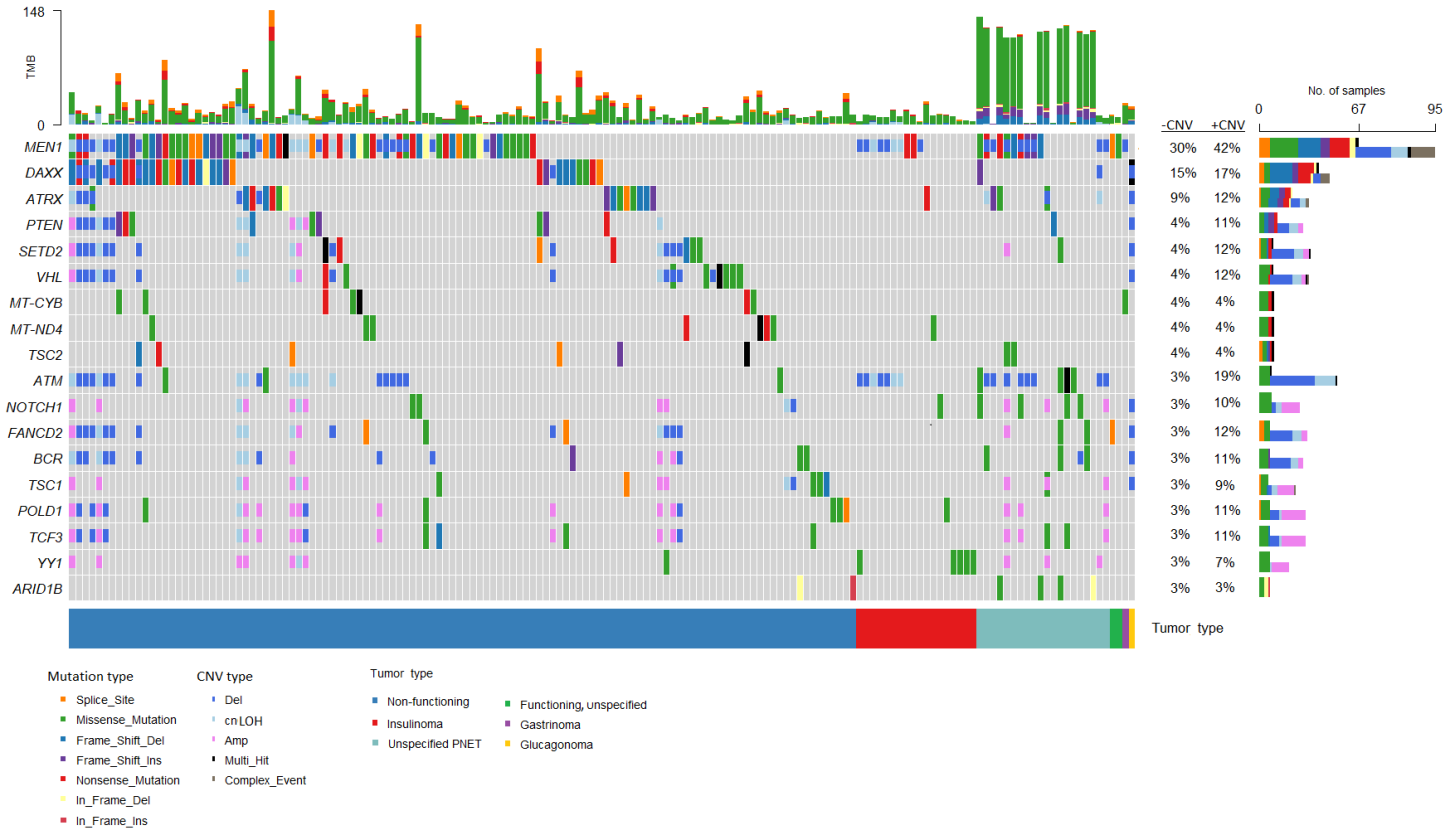
Genes frequently altered in pancreatic neuroendocrine tumors (PNETs). Oncoplot of genes altered in more than 2% of all samples are presented in the datasets (n=225). The sum of mutations annotated in each PNET is shown in the upper panel. Each colored bar indicates a somatic variant colored based on the mutation type and copy number variation as depicted. Colored filled boxes represents mutations, partly filled boxes represents copy number variations. The tumor types of each of the PNETs are shown in the lower bar. Panel on the right sums up the number of mutations (upper) and copy number variations (CNVs) (lower) identified in each specific gene. Further, the percentage of PNETs with mutations (- CNV) and mutations plus CNVs (+CNV) are presented at the right. TMB, tumor mutation burden; cnLOH, copy-neutral loss of heterozygosity; CNV, copy number variations.

**Figure 3:**
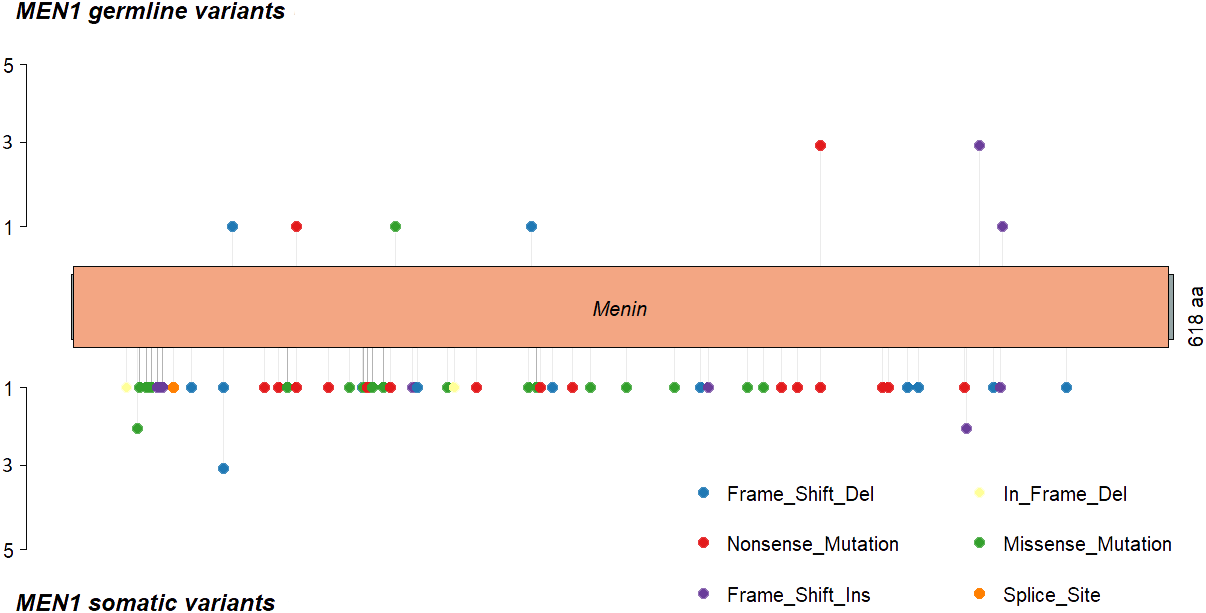
*MEN1* mutational distribution in MEN1 patients and sporadic PNETs, where *MEN1* is altered in 30% on mutational level. (Upper panel) *MEN1* mutations found in germline from 11 (73%) of MEN1 patients. One MEN1 patient harbored germline copy number variations in *MEN1*, and one MEN1 patients had a splice site variant (not shown). Lastly, one PNET had an unspecified *MEN1*-germline variant. (Lower panel) somatic *MEN1* mutations from two patient with MEN1 (p.Leu175Pro and p.G469Afs*35) and 65 sporadic PNETs. Nine somatic variants were further identified in the *MEN1* splice sites (not shown). Twelve (80%) MEN1 patients had somatic copy number variations in the *MEN1* locus. Transcript: NM_130799 (isoform 2), protein identifier: NP_570711, menin length: 610 amino acids.

The most frequently somatically altered genes in all PNETs are highlighted in **Figure 2**. The top three variated genes were *MEN1* (n=95; 42%), *DAXX* (n= 37, 17%) and *ATRX* (n=27; 12%). Somatic point mutations/indels in *MEN1* were identified in 67 PNETs and CNVs in 41 PNETs, including 13 PNETs with both a point mutation/indel and CNV in the gene. The somatic *MEN1* point mutations/indels in the coding region were distributed throughout the gene as shown in **Figure 3** (lower row).

**Figure 4:**
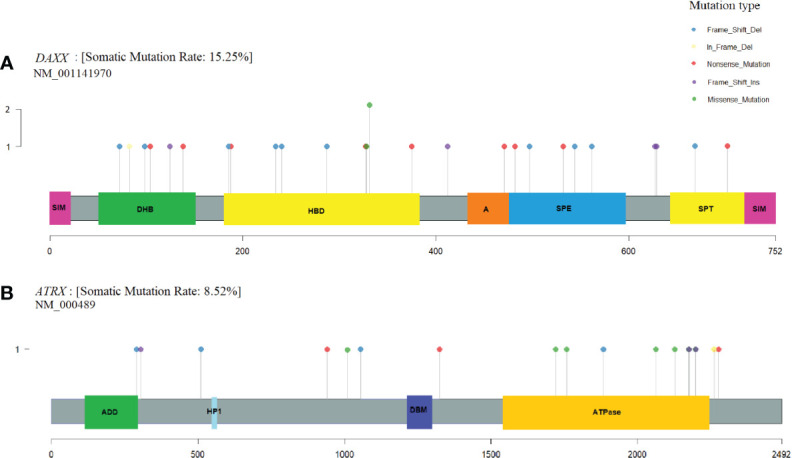
*DAXX* and *ATRX* somatic alterations in pancreatic neuroendocrine tumors (PNETs). Somatic variations uncovered in the frequently altered genes **(A)** *DAXX* (15%) and **(B)** *ATRX* (8.5%). Each protein is highlighted in gray, using the transcripts NM_001141970 and NM_000489, respectively. Domains are represented as colored boxes. The pins correspond to single somatic mutations identified in PNETs of the cohort and the color of the pin indicates the mutation type, and the height depicts the number of the variant type in the locus. Annotated domains are adapted from Wang et al. (40). Domains in DAXX: SIM, Sumo-interaction motif; DHB, DAXX helical bundle; HBD, histone binding domain; A, Acidic segment rich in Glu/Asp residues; SPE, segment rich in Ser/Pro/Glu residues; SPT, segment rich in Ser/Pro/Thr residues. Domains in ATRX: ADD, ATRX-DNMT1-DNMT1L domain; HP1, HP1-binding motif; DBM, DAXX binding motif; ATPase, ATPase domain.

Variants in the coding regions of *DAXX* and *ATRX* are presented in **Figure 4**. The *DAXX* and *ATRX* mutations were unique except for one *DAXX* alteration, which was present in two PNETs. All of the missense mutations in *DAXX* (n=3) and *ATRX* (n=6) were predicted as probably damaging by PolyPhen2. Notably, the *DAXX* missense mutations were located in close proximity to each other (amino acid 328 to 331) in the histone-binding domain. The majority of missense variants in *ATRX* (5/6) were all located in the ATPase domain spanning residues 1,550–2,226.

#### 3.3.1 Co-occurrence of mutated genes

**Figure 5:**
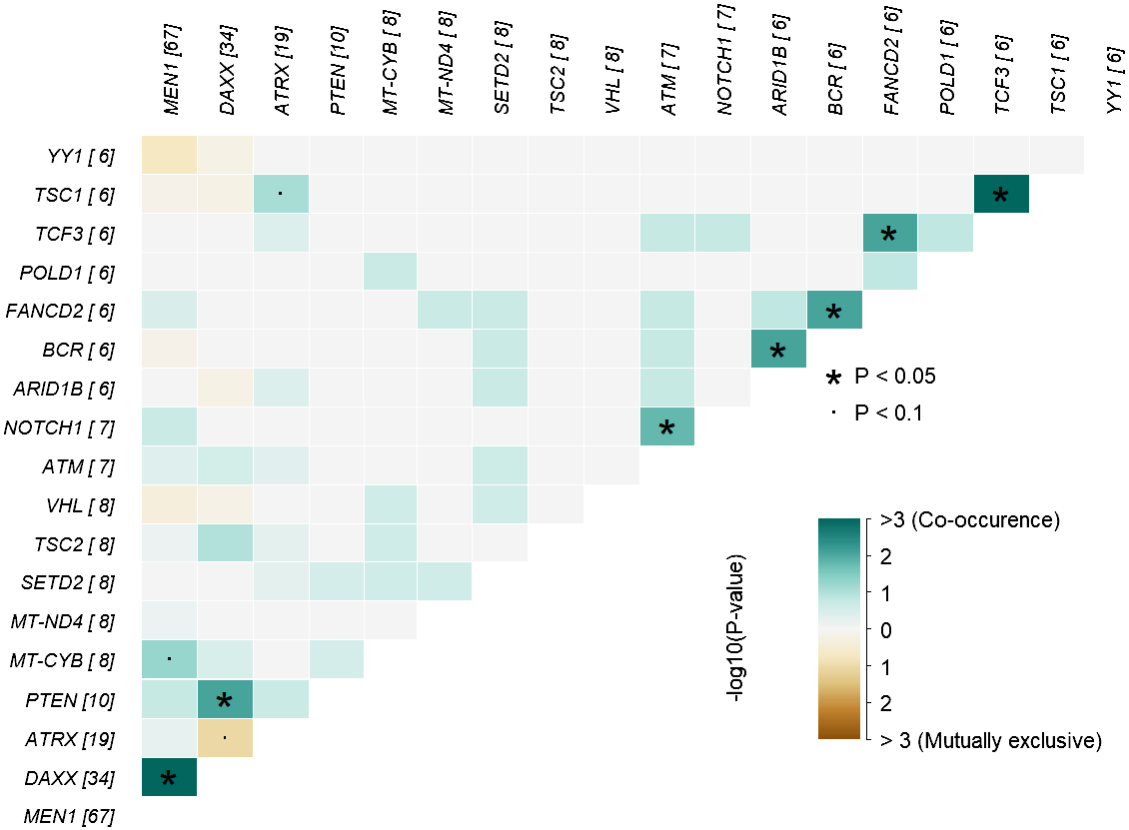
Co-occurrence plot showing mutually exclusive (brown) or co-occurring (cyan) set of altered genes (mutations) identified in PNETs. The plot shows the top of mutated genes (altered in more than 2% of PNETs) and p-values are indicated as asterics (p-value < 0.05) or dots (p-value < 0.1) determined by pair-wise Fisher’s exact test. * P-value < 0.05 and the dot represents p-value < 0.1

In analysis of co-occurrence of mutated genes, *DAXX* and *MEN1* mutations were more frequently co-occurring compared to other pairwise gene co-occurrences, p<0.05, **Figure 5**. A trend toward mutually exclusiveness for *DAXX* and *ATRX* variants was observed (p<0.1), whereas *MEN1* and *YY1* variants were mutually exclusive, however insignificant due to low numbers.

#### 3.3.2 Hereditary and sporadic PNETs

**Figure 6:**
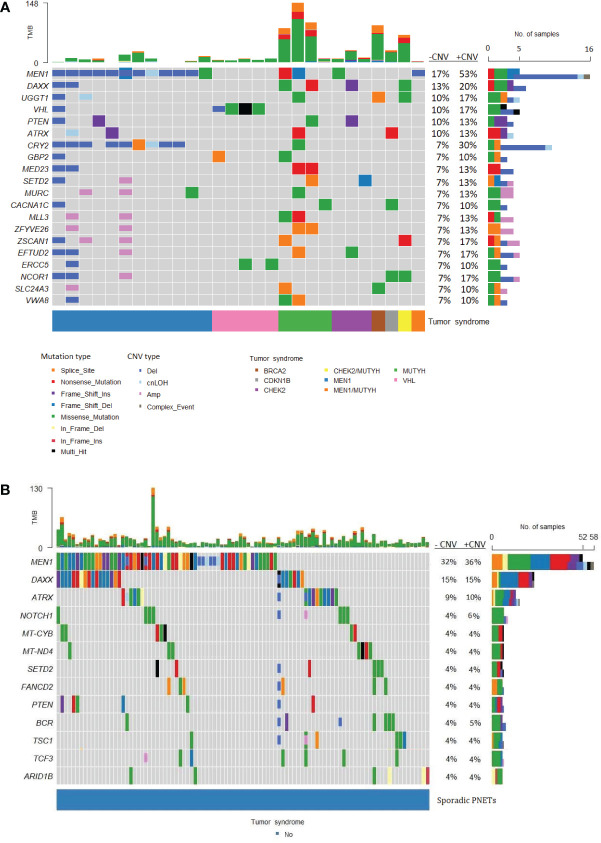
Frequently altered genes in hereditary and sporadic pancreatic neuroendocrine tumors (PNETs). The gene list is ordered after the frequency of somatic mutations in specific genes. **(A)** Data from PNETs from patients with germline mutation in *MEN1, MUTYH, CHEK2, BRCA, CDKN1B, CHEK2/MUTYH, MEN1/MUTYH* or *VHL* (n=30) and **(B)** PNETs from patients with sporadic PNETs (n=162).The tumor syndrome of each of the PNETs is shown in the lower bar. Each colored bar in the oncoplot indicates a somatic variant colored based on the mutation type or copy number variation as depicted. Filled colored boxes represent gene-specific somatic mutations, and partly colored boxes represent copy number variations in the specific gene region or chromosome. Panel on the right sums up the number of PNETs with mutations (upper), and mutations plus copy number variations (CNVs) (lower) identified in each specific gene. Likewise, the percentage is represented for PNETs with mutations (-CNV) and for PNETs with mutations and CNVs (+CNV). PNETs with more than one mutation in a gene were represented as a multi-hit (black), and genes with mutation and copy number variation were depicted as a complex event (grey). The sum of variations annotated in each PNET is shown in the upper panel. CNV, copy number variation; cnLOH, copy-neutral loss of heterozygosity; TMB, tumor mutation burden.

*MEN1* was the most frequently altered gene in both hereditary and sporadic PNETs (**Figures 6A, B**). Somatic *MEN1* mutations were found in 5/30 (16.7%) of hereditary PNETs vs. 52/162 (32.1%) of the sporadic PNETs (p=0.13).

Of the 14 hereditary PNETs from patients with a germline *MEN1* mutation, all 13 with available data had a second somatic hit in *MEN1.* CNVs in the *MEN1* region were seen in 12 (11 deletions and 1 cnLOH; one with an additional somatic *MEN1* point mutation); one had a somatic *MEN1* point mutation only. Notably, *MEN1*-germline PNETs did not share other somatic gene mutations than *MEN1*.

The germline *MEN1* mutations in the hereditary group are shown in upper row of the **Figure 3**, and constitute of 11 point mutations/indels in addition to 1 splice site variant and one 1 germline CNV in *MEN1*. One germline mutation (1/14) was unknown. As for the somatic *MEN1* mutations, the germline mutations were distributed throughout the gene. CNVs in *MEN1* were more frequently detected in hereditary compared to sporadic PNETs, 12/30 vs. 8/162, p<0.00001. CNVs were rarely reported in other genes in hereditary PNETs except for *CRY2* CNVs, which accounted 8/10 (80%).

*DAXX* and *ATRX* point mutations and CNVs occurred equally frequent in hereditary vs. sporadic PNETs, **Figures 4A, B**. Somatic mutations in *DAXX* were found in four PNETs with hereditary *CHEK2* and/or *MUTYH*. A trend toward more frequent *PTEN* mutations in hereditary vs. sporadic PNETs was observed (13% vs. 4% including CNVs, p=0.07).

The abundancy of other shared altered genes was low in both hereditary and sporadic PNETs. None of the other 17 abundant genes in hereditary PNETs were found among the 10 other genes in sporadic PNETs. Notably, three PNETs with germline *MUTYH* and one with a germline *BRCA2* variant had the highest tumor mutational burden (TMB) compared to the other germline predisposed PNETs.

#### 3.3.3 Variants in non-functioning and functioning PNETs

Non-functioning and functioning PNETs accounted for 201/225 (89.3%) of the included PNETs and 24 (10.7%) PNETs had non-specified functionality. Non-functioning PNETs accounted 156/201 (77.6%) and functioning PNETs with specified functionality 42/201 (21%). The functioning PNETs were insulinomas (n=37), glucagonomas (n=2), gastrinomas (n=2) and VIPoma (n=1).

**Figure 7:**
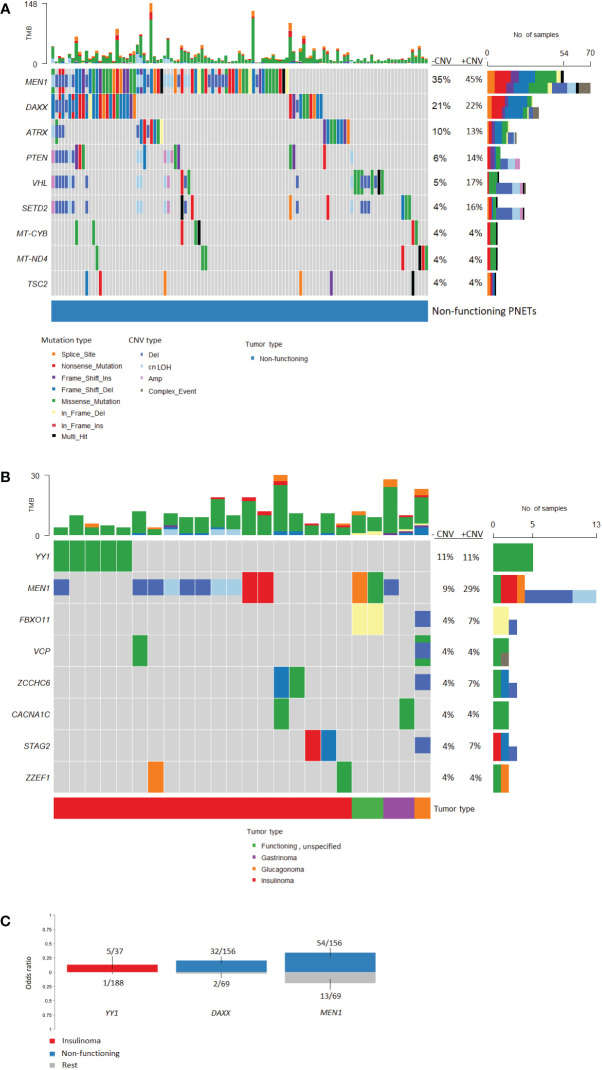
Oncoplots of somatic mutations and copy number variations identified in pancreatic neuroendocrine tumors (PNETs) divided into tumor types. Frequently altered genes identified in **(A)** non-functioning PNETs and **(B)** functioning PNETs are presented (found in more than 3%). Variant type is depicted in the barplot and the sum of mutations (upper) and the sum of mutations plus copy number variations (lower) in each gene is represented in the right bar. The percentage represents the percentage of PNETs with mutations (-CNV) and mutations plus copy number variations (+CNV) in each gene. The upper bar shows the total number of mutations in each specific PNET sample. **(C)** Groupwise comparison of gene variants based on tumor type. Copy number variations are not included in the analysis. The corresponding bars are colored by tumor type and indicates genes significantly altered (p-value < 0.05) between tumor types. The y-axis represents the odds ratio. TMB, Tumor mutation burden; CNV, copy number variation; cnLOH, copy-neutral loss of heterozygosity.

Separate mutational profiles for non-functioning and functioning PNETs are seen in **Figures 7A, B**. As for hereditary and sporadic PNETs, somatic mutations in *MEN1*, *DAXX* and *ATRX* were most frequent in non-functioning PNETs.

Functioning PNETs only shared mutations in eight genes, of which *YY1* (5/45, 11.1%) and *MEN1* (4/45, 8.9%) were the most frequently mutated (**Figure 7B**). Additionally, CNVs in *MEN1* were abundant (9/45, 20%) in functioning PNETs. However, when comparing the SNVs, *MEN1* mutations were only enriched in non-functioning PNETs (**Figure 7C**). Apart from *MEN1*, non-functioning and functioning PNETs did not share other altered genes. *DAXX* mutations were only identified in 1/45 (2.2%) of the functioning vs. 32/156 (21%) of the non-functioning PNETs, p=0.0023, highlighting the enrichment of *DAXX* mutations in non-functioning PNETs (**Figure 7C**). The single *DAXX* mutation was detected in one of the two glucagonomas.

Variants in *YY1*, *STAG2*, *ZCCHC6*, and *ZZEF1* were insulinoma-specific, when compared to other functioning PNETs. Enrichment of *YY1*-variants was significantly higher in insulinomas vs. other PNETs (**Figure 7C**). In line with this, the recurrent mutation in *YY1* (p.Thr372Arg) was identified in five insulinoma samples (5/37 insulinomas, 14%), and a single non-functioning PNET (n=1/156, accounting for 0.6% of the non-functioning PNETs), p=0.0011. No other *YY1*-variants were identified.

##### 3.3.3.1 Onco-pathways in functioning and non-functioning PNETs

**Figure 8:**
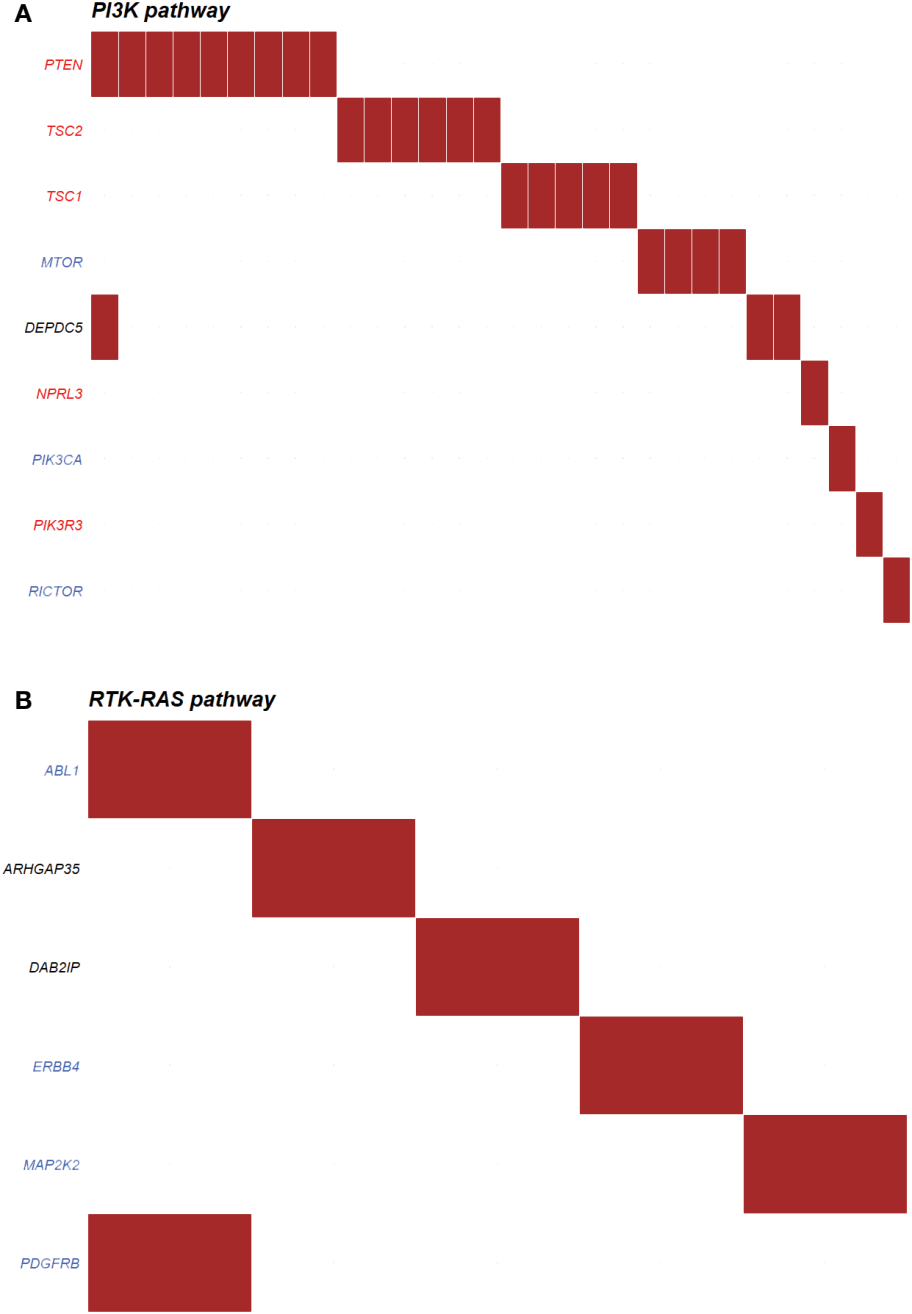
Top oncogenic pathway enriched in **(A)** non-functioning (n=30) and **(B)** functioning (n=5) pancreatic neuroendocrine tumors (PNETs). The oncogenic pathways uncovered in PNETs are based on somatic mutations. The oncogenic signaling pathways are based on pathways from The Cancer Genome Atlas (TCGA) cohorts. Genes highlighted in red are tumor suppressor genes, and genes highlighted in blue are proto-oncogenes. Each column indicates a PNET and a somatic mutation is represented by a red box in the row of the altered gene. PI3K, Phosphoinositide 3-kinase; RTK-RAS, Receptor Tyrosine Kinase-Ras.

The most enriched oncogenic pathway in non-functioning PNETs were phosphoinositide 3-kinase (PI3K) (**Figure 8A**), also known as PI3K/AKT/mTOR pathway. In this pathway, the most abundant altered genes were coding for PTEN (n=9), TSC2 (n=6), TSC1 (n=5) and MTOR (n=4). Other onco-pathways altered in more than 10% of non-functioning PNETs were Receptor Tyrosine Kinase-Ras (RTK-Ras) signaling pathway (n=20), NOTCH (n=16), and Wnt (n=16) pathways (**Supplementary Figure 2**). Few non-functioning PNETs had acquired ≥2 variants in the same pathway.

Functioning PNETs did not share any variants in relations to annotated onco-pathways, however six genes were altered in five different PNETs in association to the RTK-Ras (**Figure 8B**). No other onco-pathway was enriched for more than 10% of functioning PNETs.

## 4 Discussion

In this meta-analysis of published somatic mutations in G1/G2 PNETs, *MEN1* was the most frequently altered gene across all PNETs no matter heredity and functionality. *DAXX* and *ATRX* were also abundant in hereditary, sporadic and non-functioning PNETs, but rare in functioning PNETs, in which the recurrent *YY1* variant (p.Thr372Arg) was significantly enriched. Only non-functioning PNETs had enrichment of onco-pathways.

### 4.1 MEN1

*MEN1* point mutations was identified in 30% of the analyzed tumors and 42% when including CNVs. Consistently, previous smaller individual studies with or without CNV analyses reported a frequency of 25–44% (11–14). In PNETs from MEN1 patients, *MEN1* was somatically altered as a second hit in all 13 with available data. Inactivation of a second *MEN1* allele may happen due to LOH, point mutations, or epigenetic inactivation. In our dataset, 92% (12/13) of the MEN1 patients acquired LOH in the *MEN1* locus as the somatic second hit (one patient had no *MEN1* somatic data). Likewise, LOH has earlier been proposed to be the main mechanism of full *MEN1* inactivation, accounting for 90% of PNETs in MEN1 patients (41). MEN1 patients did not share other somatic gene mutations, which may reflect the sovereignty of menin deficiency in their PNET development.

In sporadic PNETs, Jiao et al. identified point mutations/indels in *MEN1* in 30/68 (44%) of the examined cohort. (12). In our meta-analyses, which did not include this study, 52/162 (32%) sporadic PNETs harbored somatic *MEN1* point mutations/indels. Only six sporadic PNETs had a CNV of the *MEN1* locus, and CNVs in *MEN1* were significantly more frequent in hereditary PNETs compared to sporadic PNETs. This difference may, however, be assigned the low number of CNV data in sporadic PNETs. Somatic *MEN1* alterations were equally distributed in non-functioning vs. functioning PNETs with a high frequency of CNVs in each group. This further highlights the need of CNV analyses in all subtypes of PNETs when studying their tumorigenesis.

### 4.2 DAXX and ATRX

*DAXX* and *ATRX* were the second and third most frequently altered genes in our meta-analysis. Two patients with hereditary *MEN1* had a somatic alteration in both *MEN1* and *DAXX*. The heterogeneity of *ATRX* and *DAXX* mutations has been explored earlier and it was also apparent in the cohort of PNETs. Only one variant in *DAXX* was altered in two PNETs, the remaining *DAXX*-mutations were exclusive and specific to each PNET.

A mutational hotspot in *DAXX* has earlier been proposed, resulting in alterations in the amino acid NM_001141970.1, Serine 102; p.(Ser102Leu) and p.(Ser102*) (42). In our dataset, which did not include the mentioned study, we did not identify variants altering Serine 102. Instead, we identified another hotspot for three non-synonymous missense variations in *DAXX* spanning from amino acid 328 to 331, located in a histone binding domain (amino acids 178–389) of the protein (40). Indeed, Jiao et al. (12)also identified a *DAXX* missense variant in this domain p.(Ala297Pro) and an in-frame deletion p.(Leu309GlnPhe), and Quevedo et al.(43 *)* identified a p.(Ala313Val) variant in a PNET. Taken together, the *DAXX* histone-binding domain 178–389 represents a new mutational hotspot in PNETs, although *DAXX* mutations were widespread.

*ATRX* mutations were also unique in regard to the position in the gene and were only observed once and with no tendency toward specific mutation types. The six somatic missense mutations in *ATRX* were all predicted probably damaging. Five of the identified missense mutations, (p.(His1759Asp), p.(Met1800Ile), p.(Lys2036Glu), p.(Ser2116Phe), and p.(Tyr2176Cys), were clustered in the C-terminal ATPase domain (amino acid 1,550–2,226) of *ATRX* (40). This domain contains seven conserved motifs responsible for the ATP-hydrolysis and may further be responsible for DNA translocase activity (44, 45). Experimental evidence has shown that this domain is DNA-dependent and may be associated with chromatin remodeling or DNA replication (46). The variant p.(Lys2036Glu) is placed within the highly conserved helicase domain IV (47, 48). The five mutations could have a noticeable effect on the function of ATRX, and may hinder the ATPase activity of the protein. The mutational hotspot may indicate, that this function is pivotal for endocrine cells, as the interaction with DAXX at H3.3 still may be intact. Interestingly, other PNET study data from Jiao et al. and Quevedo et al. showed a total of four *ATRX* missense variants, all clustering in the ATPase domain (12, 43), supporting the importance of the ATRX ATPase activity in PNETs.

In the sporadic cohort, *DAXX* and *ATRX* were mutually exclusive except for one PNET which had a CNV in both genes. This further strengthens the potential for *DAXX* and *ATRX* alterations as independent tumor drivers in PNETs. Furthermore, *DAXX* was altered significantly more in non-functioning compared to functioning PNETs, indicating a frequent role of *DAXX* in the tumorigenesis of non-functioning PNETs. In functioning PNETs, only one *DAXX* and one *ATRX* alteration was seen in a glucagonoma, and an insulinoma, respectively. *ATRX* and *DAXX* mutations has previously been associated with a more alpha cell-like phenotype in a study not included in the present meta-analysis (49).

### 4.3 YY1

*YY1* mutations was especially frequent in insulinomas, in which 11% harbored a point mutation or indel. Mutations in *YY1* was less frequently seen in the meta-analysis compared to the included Asian study of Cao et al., where up to 30% of insulinomas had *YY1* alterations (15). The recurrent *YY1* mutation p.(Thr372Arg) occurred in 14% in our analysis, narrowing the frequency of 8–30% of reported in three individual studies, of which only the study by Cao et al. was included in our meta-analysis (15–17). Of note, Lichtenhauer et al. found a lower prevalence of *YY1* alterations of 12% in insulinomas from their Caucasian cohort (16), compared to sporadic insulinomas from an Asian cohort (15).

Surprisingly, *YY1* was not exclusively altered in insulinomas in our datasets. One non-functioning tumor also harbored the characteristic p.(Thr372Arg) mutation, and the *YY1* region was amplified in nine, especially non-functioning, PNETs. The latter may indicate that *YY1* could be a proto-oncogene just as observed for the gene amplifications and overexpression of *MDM2* in cancer (50). *YY1* codes for the potential proto-oncogenic ying yang 1 transcription factor (51), confirming the relevance of *YY1* in tumorigenesis.

### 4.4 Other genes

In hereditary PNETs from other than MEN1 patients, germline mutations were observed in *MUTYH*, *CHEK2*, *BRCA2*, *VHL*, *PTEN*, and *CDKN1B*, of which one had germline mutations in both *CHEK2* and *MUTYH*. Furthermore, one MEN1 patient had germline mutations in both *MEN1* and *MUTYH*. Moreover, *DAXX* variants occurred in four patients with *CHEK2* and/or *MUTYH* germline presentation, highlighting the need of expanded genetics in PNET tumorigenesis research.

Of the six patients with germline *MUTYH* variants, three of the variants were predicted as pathogenic (13). Our datasets indicated a high level of tumor mutational burden in these individuals, even though the variant numbers between datasets should be compared with caution.

While the MUTYH protein is involved in base-excision-repair during DNA damage (52), BRCA2 and CHK2 are members of the homologous recombination pathway, repairing double-stranded DNA breaks (53). A *BRCA* germline variant was detected in one patient, and *CHEK2* germline variants were identified in four patients. Genomic instability caused by defective DNA repair proteins is a well-described hallmark of cancer (54). Accordingly, patients with germline variants in *MUTYH*, *BRCA2* or *CHEK2* should be considered at risk of PNET development, as well as at risk for hereditary cancer (55). The presence of *BRCA2* germline variants in pancreatic lesions, including PNETs, has been described earlier (13, 14, 56–58).

Germline mutations in *VHL* causing von-Hippel Lindau syndrome appeared in five patients from our datasets. Development of PNETs in this rare syndrome is seen in 17% (273/2,330) of all VHL patients according to The European-American-Asian-VHL-panNET-Registry (59, 60). Especially variants in exon 3 have been associated with malignancy (59, 61). Of the five patients with a *VHL* germline alteration, one had a missense variant in exon 3 and somatic LOH of the second allele and was metastasis-free (13). Somatically, we identified eight *VHL* variants in the whole dataset, of which one was a frameshift mutation and seven were missense variants distributed in all three exons. However, only a single of these PNETs had metastasis and this variant was in exon 2. Seven of the eight PNETs with *VHL* variants were non-functioning. This predominance of non-functioning PNETs is in line with other studies (61, 62).

Notably, one PNET-patient included in our datasets, had been diagnosed with Cowden syndrome, harboring a deleterious germline *PTEN*-variant (34). A case report of the patient has been published (63), highlighting the rarity of the association of PNET development and the syndrome. Lastly, one PNET harbored a germline variant in *CDKN1B*. Loss of germline *CDKN1B* is referred to as MEN4 syndrome and can cause a MEN1-like phenotype. Only very few PNETs have been described having germline variants in *CDKN1B* (64, 65). Notably, patients with hereditary PNETs were significantly younger than patients with sporadic PNETs, as described earlier (1, 66).

### 4.5 Enriched pathways

PNETs, which are mostly non-functioning, have been associated with variants in AKT/PI3K/mTOR pathway, i.e*. PTEN, TSC1, TSC2, PIK3CA* and *DEPDC5*, which regulates cell survival and proliferation (13, 67). It has been suggested that patients with mTOR pathway aberrations may benefit from therapy using mTOR inhibitors (12, 13). In our combined datasets, we also found variants in the PI3K/mTOR pathway, especially in the non-functioning tumors (30/156 PNETs). The most abundant altered genes were coding for PTEN (n=9), TSC2 (n=6), TSC1 (n=5) and MTOR (n=4). Only one tumor had mutations in more than one gene encoding a PI3K/mTOR pathway interactor. Interestingly, the use of everolimus in non-functioning NETs improved the progression-free survival compared to placebo (11 vs 3.9 months, RADIANT-4 trial, (68)), and although the documented use of everolimus in functioning NETs is sparse, mTOR-inhibitor treatment may also affect the clinical symptoms, independent of restrain of tumor growth (69, 70).

RTK-Ras (n=20), Wnt pathway (n=16) and NOTCH (n=16) pathways were also altered in more than 10% of non-functioning PNET. While most of the NOTCH associated proteins were tumor suppressors, the RTK-Ras pathway mostly consists of proto-oncogenes (**Supplementary Figure 2**), and a maximum of three non-functioning PNETs had mutations in the same gene (*ERBB2*, *AXIN2*, and *NOTCH2*), thereby indicating common pathway variations, but not necessarily gene specific alterations also among the non-functioning tumors. The identification of aberrant pathways may in turn give insight into potential targets for drug designs in the future. Drugs targeting the distinct protooncogenic pathways RTK-Ras, Wnt and NOTCH do already exist. These include for example the multi-kinase inhibitor sorafenib (71), which may impact the entire pathway. For the Wnt and NOTCH-pathways only very few FDA approved drugs are available, however a few new pathway-specific drugs have entered clinical trials (72, 73).

### 4.6 Strengths and limitations

Strengths our study included the systematic search for eligible articles and, by nature of a meta-analysis, our large sample size from 14 datasets compared to individual datasets. Moreover, our inclusion criteria of reported G1/G2 PNETs ensured a focus on wd-PNETs, as G3 PNET is a newly defined entity, not incorporated in most previous studies.

In the WHO version of 2010, wd-NETs of low to intermediate grade was defined as G1-G2, whereas poorly differentiated, high-grade neoplasms were defined as G3 (74). In 2017, WHO altered the definition and introduced a G3 NET described as well-defined with a distinct morphology compared to G3 neuroendocrine carcinomas (NECs) (75). G3-NET shares a molecular mutation profile similar to G1 and G2 NET and are associated with better survival than the poorly differentiated G3 NEC (76), which also has a mutational profile more similar to pancreatic ductal adenocarcinomas (77). G3 NETs may be difficult to distinguish from (G3) NECs, as both entities share a high proliferation rate (Ki67 proliferation index > 20% and/or mitotic rate above 20 per high power field) (78). New studies on wd-PNETs should, however, include G3 PNETs according to the new WHO classification.

Limitations included the varying methods in the included articles, which may have affected the results. The variations include different tissue preparations (FFPE or frozen), different type of library preparations (with or without gene enrichment), different sequencing platform, and resultant different read depth. Six of the articles did not describe the average read depth, although for one article the quality of the sequencing was described using Q_30_ (27), which indicates the likelihood of incorrect base calling in 1 of 1000 times (79). The differences between the article’s methods may be reflected by the variant calling, as some articles presented with very few variants (28, 29, 34) compared to others (32, 33, 80). Of note, after retrieving the variants, we excluded synonymous and intronic variants, even though such variants can alter the splicing of genes (81).

Moreover, CNV information could only be gathered from 33% of the PNETs, of which we only included structural variations, i.e. amp, del, and cnLOH as structural variations, e.g. tandem duplications, inversions, and translocations, have unpredictable consequences and such variations were only described in one of the 14 included studies (n=33) (32). Underreporting of clinically important CNVs could, therefore, not be ruled out.

Lastly, patients without a known germline predisposition were termed ‘sporadic’, even though most articles did not specify other germline gene results but for *MEN1*. This may lead to underreporting of rarer germline gene variants in PNETs such as *VHL*, *PTEN*, *CDKN1B*, *BRCA2*, *CHEK2*, and *MUTYH*.

## 5 Conclusion

In 225 G1/G2 PNETs, *MEN1* was most frequently somatically altered in all patient groups. *DAXX* and *ATRX* were abundant in hereditary, sporadic and non-functioning PNETs, but rare in functioning PNETs. Whereas *MEN1* mutations were distributed throughout the gene, *DAXX*, and *ATRX*, missense variants were clustered in mutational hotspots associated with histone binding, and translocase activity, respectively. In functioning PNETs, the well-known *YY1* variant (p.Thr372Arg) was significantly enriched while few other gene alterations were shared. Non-functioning PNETs had more recurrent variations in genes associated with the PI3K, Wnt, NOTCH, and RTK -Ras signaling onco-pathways.

Our review of PNET variations may contribute to the overall understanding of the genetic alterations in PNETs. Future studies on expanded genetics in PNETs should precisely describe the functional status and germline dispositions, including not only *MEN1*, but expanded germline gene analyses, and expanded CNV analyses of the PNETs. Genotype-phenotype correlations should be strengthened not only for PNETs with a single gene alteration, but also for PNETs with co-occurrence of more than one possibly oncogenic gene alteration. The meta-analysis could be helpful in the search for new targeted treatment approaches in PNETs. Future approaches to characterize PNETs could involve genomic, transcriptomic, proteomic and importantly epigenetic analyses to better understand the complexity of the tumorigenesis.

## Data availability statement

Publicly available datasets were analyzed in this study. This data can be found here: (13): PMID: 28199314 DOI: 10.1038/nature21063; (15): PMID: 24326773 DOI: 10.1038/ncomms3810; (27): PMID: 29435419 DOI: 10.1002/2211-5463.12366; (19): PMID: 28974674 DOI: 10.1038/s41467-017-00992-9; (28): PMID: 28503312 DOI: 10.1038/hgv.2017.13; (29): PMID: 33840689 DOI: 10.2220/biomedres.42.89; (30): PMID: 20857520 DOI: 10.3748/wjg.v16.i36.4515; (14): PMID: 33747156 DOI: 10.3892/etm.2021.9859; (31): PMID: 29725435 DOI: 10.3892/ol.2018.8184; (32): PMID: 34880079 DOI: 10.1158/2159-8290.CD-21-0669; (33): PMID: 35794609 DOI: 10.1186/s12967-022-03511-7; (34): PMID: 30865533 DOI: 10.4158/EP-2018-0603; (35): PMID: 34644566 DOI: 10.1016/j.celrep.2021.109817; (36): PMID: 37099786 DOI: 10.1097/MPA.0000000000002199.

## Author contributions

KA: Conceptualization, Data curation, Formal analysis, Project administration, Software, Writing – original draft, Writing – review & editing. SD: Supervision, Writing – review & editing. KB: Data curation, Supervision, Writing – review & editing. HC: Supervision, Writing – original draft, Writing – review & editing.
