# Supplementary material for: Well-differentiated G1 and G2 pancreatic neuroendocrine tumors: a meta-analysis of published expanded DNA sequencing data
Source: Front Endocrinol (Lausanne). 2024 May 29;15:1351624. doi: 10.3389/fendo.2024.1351624 (PMC11167081; doi:10.3389/fendo.2024.1351624)
Supplement: Supplementary file 1 [file DataSheet_1.docx]

Supplementary Material

# Supplementary Data

# Supplementary Tables

# Supplementary Table 1. Excluded datasets and articles based on title and abstract.

|  | **Exclusion criterion:** | | |  |
| --- | --- | --- | --- | --- |
| **Author** | **No human PNET (G1-G2)/only cell lines/animal models** | **No DNA sequencing** | **Only screening of single gene or panel (<400 genes)** | **Reference** |
| Zhou *et al.*  (2021) |  | No DNA sequencing of PNET tissue |  | (Zhou, Liu et al. 2021) |
| Terracciano *et al.*  (2021) | Cell line study |  |  | (Terracciano, Capone et al. 2021) |
| Mahjoub *et al.*  (2013) |  | No DNA sequencing of PNET tissue |  | (Mahjoub and O'Reilly 2013) |
| Shan *et al.*  (1998) |  |  | Only MEN1 gene screening | (Shan, Nakamura et al. 1998) |
| Mönig *et al.*  (1993) |  | Sequencing of extra-pancreatic tissue |  | (Mönig, Ali et al. 1993) |
| Kawasaki *et al.*  (2020) | No PNET sample |  |  | (Kawasaki, Toshimitsu et al. 2020) |
| Birla *et al.*  (2015) |  |  | Only MEN1 gene screening | (Birla, V et al. 2015) |
| Zhang *et al.*  (2022) |  |  | Only MEN1 gene screening | (Zhang, Yu et al. 2022) |
| Yang *et al.*  (2009) |  | No WES or WGS |  | (Yang, Zhou et al. 2009) |
| Evers *et al.*  (1994) |  | No WES or WGS |  | (Evers, Rady et al. 1994) |
| Jiang *et al.*  (2007) |  |  | Only MEN1 gene screening | (Jiang, Lu et al. 2007) |
| Kawamura *et al.*  (2005) |  |  | Only MEN1 gene screening | (Kawamura, Shimada et al. 2005) |
| Ding *et al.*  (2002) | Extra-pancreatic tissue |  |  | (Ding, Cheng et al. 2002) |
| Raoul *et al.*  (2021) | Extra-pancreatic tissue |  |  | (Raoul, Heymann et al. 2021) |
| Raghavan *et al.*  (2007) |  |  | Only MEN1 gene screening | (Raghavan, Shah et al. 2007) |
| Shaw *et al.*  (1993) |  | No WES or WGS |  | (Shaw, Cormican et al. 1993) |
| Öberg |  | No WES or WGS |  | (Oberg 2018) |
| Nielsen *et al.*  (1990) | No human samples |  |  | (Nielsen, Welinder et al. 1990) |
| Lu *et al.*  (1998) | Cell lines only |  |  | (Lu, Li et al. 1998) |
| Manialawy *et al.*  (2020) |  | No WES or WGS |  | (Manialawy, Khan et al. 2020) |
| Miller *et al.*  (2015) |  | No WES or WGS |  | (Miller, Kidd et al. 2015) |
| Lindberg *et al.*  (2007) |  |  | Only panel sequencing | (Lindberg, Akerström et al. 2007) |
| Uttenthal *et al.*  (1985) |  | No WES or WGS |  | (Uttenthal, Ghiglione et al. 1985) |
| Zhou *et al.*  (2009) |  |  | Only panel sequencing | (Zhou, Dhall et al. 2009) |
| Donadel *et al.*  (1998) | Only cell lines |  |  | (Donadel, Marinos et al. 1998) |
| Lou *et al.*  (2022) | Only cell lines |  |  | (Lou, Ye et al. 2022) |
| Chatani *et al.*  (2020) |  | No WES or WGS |  | (Chatani, Agarwal et al. 2020) |
| Choi *et al.*  (2022) |  | No WES or WGS |  | (Choi and Paik 2022) |
| Schmitz *et al.*  (2021) | Cell lines/mice only |  |  | (Schmitz, Weissbach et al. 2021) |
| Vandamme *et al.*  (2015) | Cell lines only |  |  | (Vandamme, Peeters et al. 2015) |
| Luley *et al.*  (2020) | Cell lines only |  |  | (Luley, Biedermann et al. 2020) |
| Umetsu *et al.*  (2023) | Not G1/G2 tumor |  |  | (Umetsu, Kakar et al. 2023) |
| Tran *et al.*  (2021) |  | No WES or WGS |  | (Tran, Scott et al. 2021) |
| Yan *et al.*  (2020) |  | No WES or WGS |  | (Yan, Yu et al. 2020) |
| Gleeson *et al.*  (2017) |  |  | Only panel sequencing | (Gleeson, Voss et al. 2017) |
| Niemeijer *et al.*  (2015) |  | No WES or WGS |  | (Niemeijer, Papathomas et al. 2015) |
| Cloyd *et al.*  (2020) |  | No WES or WGS |  | (Cloyd, Wiseman et al. 2020) |
| Tirosh *et al.*  (2019) |  | No WES or WGS |  | (Tirosh, Mukherjee et al. 2019) |
| Hackeng *et al.*  (2016) |  | No WES or WGS |  | (Hackeng, Hruban et al. 2016) |
| Vandamme *et al.*  (2015) | Cell lines only |  |  | (Vandamme, Beyens et al. 2015) |
| Pea *et al.*  (2015) |  | No WES or WGS |  | (Pea, Hruban et al. 2015) |
| Alonso-Gordoa *et al.*  (2015) |  | No WES or WGS |  | (Alonso-Gordoa, Díez et al. 2015) |
| Williamson *et al.*  (2019) | No G1/G2 PNET |  |  | (Williamson, Steel et al. 2019) |
| Liu *et al.*  (2014) |  | No WES or WGS |  | (Liu, Tang et al. 2014) |
| Yuan *et al.*  (2014) |  |  | Only panel sequencing | (Yuan, Shi et al. 2014) |
| Szkukalek *et al.*  (2021) | Not G1/G2 PNET |  |  | (Szkukalek, Dóczi et al. 2021) |
| Iyer *et al.*  (2017) | Cell lines only |  |  | (Iyer, Modali et al. 2017) |
| Shell *et al.*  (2017) |  | No WES or WGS |  | (Shell, Patel et al. 2017) |
| Sun *et al.*  (2022) | No human samples |  |  | (Sun, Estrella et al. 2022) |
| Finnerty *et al.*  (2019) |  | No WES or WGS |  | (Finnerty, Moore et al. 2019) |
| Boora *et al.*  (2015) | Cell lines only |  |  | (Boora, Kanwar et al. 2015) |
| Suemitsu *et al.*  (2021) | No PNET sample |  |  | (Suemitsu, Ono et al. 2021) |
| Kooblall *et al.*  (2021) |  |  | Only MEN1 screening | (Kooblall, Boon et al. 2021) |
| Lagarde *et al.*  (2022) |  |  | Only MEN1 screening | (Lagarde, Mougel et al. 2022) |
| Koyama *et al.*  (2022) |  |  | Only MEN1 screening | (Koyama, Nagase et al. 2022) |
| Ma *et al.*  (2020) |  |  | Only MEN1 screening | (Ma, Guo et al. 2020) |
| Seabrook *et al.*  (2022) |  | Only germline sequencing |  | (Seabrook, Wijewardene et al. 2022) |
| Li *et al.*  (2021) |  | No WES or WGS of PNET |  | (Li, Zhang et al. 2021) |
| Li *et al.*  (2018) |  |  | Only screening of GCGR and ARG1 | (Li, Zhao et al. 2018) |
| Boratto *et al.*  (2020) |  | No WES or WGS of PNET |  | (Boratto, Cardoso et al. 2020) |
| Saoud *et al.*  (2023) | No PNET sample |  |  | (Saoud, Wu et al. 2023) |
| Balogh *et al.*  (2004) |  | No WES or WGS |  | (Balogh, Patócs et al. 2004) |
| Demirtaş *et al.*  (2020) |  | No WES or WGS |  | (Demirtaş, Ata et al. 2020) |
| Regnell *et al.*  (2017) | No PNET |  |  | (Regnell and Lernmark 2017) |
| Liang *et al.*  (2018) |  | No WES or WGS |  | (Liang, Jiang et al. 2018) |
| Zhou *et al.*  (2018) |  | No WES or WGS |  | (Zhou, Gong et al. 2018) |
| Mathew *et al.*  (2022) | No PNET |  |  | (Mathew, Bowman et al. 2022) |
| Xin *et al.*  (2018) | No PNET |  |  | (Xin, Zhang et al. 2018) |
| Salinno *et al.*  (2021) | No PNET |  |  | (Salinno, Büttner et al. 2021) |
| Rafaeloff *et al.*  (1997) |  | No WES or WGS |  | (Rafaeloff, Pittenger et al. 1997) |
| Wang *et al.*  (2004) |  | No WES or WGS |  | (Wang, Xu et al. 2004) |
| Capodanno *et al.*  (2020) |  | No WES or WGS of PNET |  | (Capodanno, Buishand et al. 2020) |
| Bristulf *et al.*  (1994) | Only cell lines |  |  | (Bristulf, Gatti et al. 1994) |
| Iida *et al.*  (2022) | No PNET |  |  | (Iida, Nakanishi et al. 2022) |
| Song *et al.*  (2017) |  | No WES or WGS |  | (Song, Yu et al. 2017) |
| Cram *et al.*  (1999) |  | No WES or WGS |  | (Cram, McIntosh et al. 1999) |
| Desai *et al.*  (2015) | No human PNET |  |  | (Desai, Kharade et al. 2015) |
| Wang *et al.*  (2022) |  | No WES or WGS |  | (Wang, Karakose et al. 2022) |
| Kim *et al.*  (2017) |  |  | Only MEN1 gene screening | (Kim, Park et al. 2017) |
| Kudo *et al.*  (2021) | No PNET |  |  | (Kudo, Takano et al. 2021) |
| Lee *et al.*  (1999) | Only cell lines/mice |  |  | (Lee, Kim et al. 1999) |
| Wang *et al.*  (2022) | Only cell lines |  |  | (Wang, Li et al. 2022) |
| Vizziano-Cantonnet *et al.*  (2018) | No human PNET |  |  | (Vizziano-Cantonnet, Lasalle et al. 2018) |
| Murat Cde *et al.*  (2015) |  | No WES or WGS |  | (Murat Cde, da Rosa et al. 2015) |
| Ediger *et al.*  (2014) | No human PNET |  |  | (Ediger, Du et al. 2014) |
| Wang *et al.*  (2019) | Only cell lines |  |  | (Wang, Jiang et al. 2019) |
| Hasani-Ranjbar *et al.*  (2011) |  |  | Only MEN1 gene screening | (Hasani-Ranjbar, Amoli et al. 2011) |
| Zhao *et al.*  (2021) | Only cell lines |  |  | (Zhao, Carmean et al. 2021) |
| Huang *et al.*  (2022) | No PNET |  |  | (Huang, Howard et al. 2022) |
| Challis *et al.*  (2014) |  |  | Only GCK gene screening | (Challis, Harris et al. 2014) |
| Jyotsna *et al.*  (2015) |  |  | Only MEN1 gene screening | (Jyotsna, Malik et al. 2015) |
| Hameed *et al.*  (2011) | No PNET |  |  | (Hameed, Ellard et al. 2011) |
| Okamoto *et al.*  (2002) |  |  | Only MEN1 gene screening | (Okamoto, Tamada et al. 2002) |
| Katashima *et al.*  (2021) | No PNET |  |  | (Katashima, Matsumoto et al. 2021) |
| Song *et al.*  (2021) |  |  | Only YY1 gene screening | (Song, Xu et al. 2021) |
| Cromer *et al.*  (2015) |  | No WES or WGS |  | (Cromer, Choi et al. 2015) |
| Baran-Gale *et al.*  (2013) | No PNET |  |  | (Baran-Gale, Fannin et al. 2013) |
| Karakose *et al.*  (2020) |  | No WES or WGS |  | (Karakose, Wang et al. 2020) |
| Moon *et al.*  (2015) | No PNET |  |  | (Moon, Lee et al. 2015) |
| Ackeifi *et al.*  (2020) | No PNET |  |  | (Ackeifi, Swartz et al. 2020) |
| Irshad *et al.*  (2017) |  |  | Only YY1 gene screening | (Irshad, Jyotsna et al. 2017) |
| Nishi *et al.*  (1992) |  | No WES or WGS |  | (Nishi, Sanke et al. 1992) |
| Carlsson *et al.*  (2020) | No PNET |  |  | (Carlsson, Shepherd et al. 2020) |
| Beck *et al.*  (2019) | No PNET |  |  | (Beck, Chandi et al. 2019) |
| Lee *et al.*  (1998) | Only cell lines |  |  | (Lee, Bradford et al. 1998) |
| Mandal *et al.*  (2020) | No PNET |  |  | (Mandal, De et al. 2020) |
| Xu *et al.*  (2010) |  |  | Only MEN1 gene screening | (Xu, Li et al. 2010) |
| Moore *et al.*  (2012) | No PNET |  |  | (Moore, Cunha et al. 2012) |
| Kelly *et al.*  (2018) | No PNET |  |  | (Kelly, Bidwell et al. 2018) |
| Andersson *et al.*  (2020) | No PNET |  |  | (Andersson Svärd, Maziarz et al. 2020) |
| Pivovarcikova *et al.*  (2019) | No PNET |  |  | (Pivovarcikova, Agaimy et al. 2019) |
| Kawasaki *et al.*  (2000) | No PNET |  |  | (Kawasaki, Sera et al. 2000) |
| MenN *et al.*  (2019) | No human PNET |  |  | (Men, Sun et al. 2019) |
| Xie *et al.*  (2002) | No PNET |  |  | (Xie, Cai et al. 2002) |
| Philipson *et al.*  (1993) |  | No WES or WGS |  | (Philipson, Kusnetsov et al. 1993) |
| Durinovic-Bellò *et al.*  (1994) | No PNET |  |  | (Durinovic-Bellò, Steinle et al. 1994) |
| Shah *et al.*  (2008) |  |  | Only MEN1 gene screening | (Shah, Raghavan et al. 2008) |
| MacDonald *et al.*  (2004) | No PNET |  |  | (MacDonald, Husain et al. 2004) |
| Durning *et al.*  (2016) | Only cell lines |  |  | (Durning, Flanagan-Steet et al. 2016) |
| Kubi *et al.*  (2019) | No PNET |  |  | (Kubi, Chen et al. 2019) |
| Muntasell *et al.*  (2002) |  | No WES or WGS |  | (Muntasell, Carrascal et al. 2002) |
| Archetti *et al.*  (2015) |  | No WES or WGS |  | (Archetti, Ferraro et al. 2015) |
| Saeed *et al.*  (2023) | No PNET |  |  | (Saeed, Mohammed et al. 2023) |
| Perren *et al.*  (2006) |  |  | Only NF1 gene screening | (Perren, Wiesli et al. 2006) |
| Giacché *et al.*  (2012) |  |  | Only MEN1 gene screening | (Giacché, Panarotto et al. 2012) |
| Scharfmann *et al.*  (1993) |  | No WES or WGS |  | (Scharfmann, Tazi et al. 1993) |
| Schuppe *et al.*  (1999) |  | No WES or WGS |  | (Schuppe, Neumann et al. 1999) |
| Wild *et al.*  (2001) |  | No WES or WGS |  | (Wild, Langer et al. 2001) |
| Vessey *et al.*  (1994) |  | No WES or WGS |  | (Vessey, Jones et al. 1994) |
| Matsutani *et al.*  (1996) |  | No WES or WGS |  | (Matsutani, Takeuchi et al. 1996) |
| Tateno *et al.*  (2001) | No PNET |  |  | (Tateno, Fukunishi et al. 2001) |
| Hai *et al.*  (2001) |  |  | Only MEN1 gene screening | (Hai, Muto et al. 2001) |
| Raymond *et al.*  (2021) |  | No WES or WGS |  | (Raymond, Korzun et al. 2021) |
| Dijkstra *et al.*  (2021) | No PNET |  |  | (Dijkstra, van den Berg et al. 2021) |
| Venizelos *et al.*  (2021) | No G1/G2 PNET |  |  | (Venizelos, Elvebakken et al. 2021) |
| Scoville *et al.*  (2020) |  | No WES or WGS |  | (Scoville, Cloyd et al. 2020) |
| Taboada *et al.*  (2022) | No G1/G2 PNET |  |  | (Taboada, Claro et al. 2022) |
| Panarelli *et al.*  (2022) |  | No WES or WGS |  | (Panarelli, Tyryshkin et al. 2019) |
| Kiesewetter *et al.*  (2020) |  | No WES or WGS |  | (Kiesewetter and Raderer 2020) |
| Busico *et al.*  (2020) | No G1/G2 PNET |  |  | (Busico, Maisonneuve et al. 2020) |
| Flaum *et al.*  (2016) |  | No WES or WGS |  | (Flaum, Valle et al. 2016) |
| Kong *et al.*  (2022) |  | No WES or WGS |  | (Kong and Hicks 2022) |
| Wang *et al.*  (2018) |  | No WES or WGS |  | (Wang, Chen et al. 2018) |
| Fortunati *et al.*  (2023) |  | No WES or WGS |  | (Fortunati, Bonazzi et al. 2023) |
| Ramage *et al.*  (2018) |  | No WES or WGS |  | (Ramage, Naraev et al. 2018) |
| Cloyd *et al.*  (2020) |  | No WES or WGS |  | (Cloyd, Ejaz et al. 2020) |
| Cives *et al.*  (2016) |  | No WES or WGS |  | (Cives, Simone et al. 2016) |
| Gajate *et al.*  (2017) |  | No WES or WGS |  | (Gajate, Martínez-Sáez et al. 2017) |
| Viol *et al.*  (2022) | No human PNET |  |  | (Viol, Sipos et al. 2022) |
| Prisciandaro *et al.*  (2022) | No G1/G2 PNET |  |  | (Prisciandaro, Antista et al. 2022) |
| Shulkes (1994) |  | No WES or WGS |  | (Shulkes 1994) |
| Hofving *et al.*  (2018) | Only cell lines |  |  | (Hofving, Arvidsson et al. 2018) |
| Kövesdi *et al.*  (2019) |  |  | Only MEN1 screening | (Kövesdi, Tóth et al. 2019) |
| Cuyle *et al.*  (2018) |  | No WES or WGS |  | (Cuyle and Prenen 2018) |
| Jeong *et al.*  (2023) |  | No WES or WGS |  | (Jeong, Park et al. 2023) |
| Sun *et al.*  (2022) | No G1/G2 PNET |  |  | (Sun, Zhao et al. 2022) |
| Tran *et al.*  (2022) | No human PNET |  |  | (Tran, Borbon et al. 2022) |
| Patel *et al.*  (2016) |  | No WES or WGS |  | (Patel, Chan et al. 2016) |
| Strosberg *et al.*  (2015) |  | No WES or WGS |  | (Strosberg, Goldman et al. 2015) |
| Ungefroren *et al.*  (2022) | No PNET |  |  | (Ungefroren, Künstner et al. 2022) |
| Walther *et al.*  (2014) |  |  | Only GNAS1 screening | (Walther, Walther et al. 2014) |
| Schaaf *et al.*  (2007) |  |  | Only MEN1 screening | (Schaaf, Pickel et al. 2007) |
| Dizdar *et al.*  (2019) |  |  | Only BRAF screening | (Dizdar, Werner et al. 2019) |
| Venizelos *et al.*  (2023) | No G1/G2 PNET |  |  | (Venizelos, Sorbye et al. 2023) |
| Saif *et al.*  (2019) |  | No WES or WGS |  | (Saif, Parikh et al. 2019) |
| Pardi *et al.*  (2017) |  |  | Only shallow panel | (Pardi, Borsari et al. 2017) |
| Boons *et al.*  (2022) | Only public PNET data |  |  | (Boons, Vandamme et al. 2022) |
| Lamberti *et al.*  (2023) | No G1/G2 PNET |  |  | (Lamberti, Prinzi et al. 2023) |
| Gerard *et al.*  (2021) | No G1/G2 PNET |  |  | (Gerard, Garcia et al. 2021) |
| Cinque *et al.*  (2017) |  |  | Only MEN1 screening | (Cinque, Sparaneo et al. 2017) |
| Miranda *et al.*  (2023) |  |  | Only MEN1 screening | (Miranda, Valadares et al. 2023) |
| Iyer *et al.*  (2017) |  | No WES or WGS |  | (Iyer, Phan et al. 2017) |
| Singh *et al.*  (2023) |  | No WES or WGS |  | (Singh, Hope et al. 2023) |
| Knappskog *et al.*  (2023) | No PNET |  |  | (Knappskog, Grob et al. 2023) |
| Wei *et al.*  (2018) |  | No WES or WGS |  | (Wei, Hua et al. 2018) |
| Orr-Asman *et al.*  (2017) | Mouse xenograft |  |  | (Orr-Asman, Chu et al. 2017) |
| Yu *et al.*  (1991) | No human PNET |  |  | (Yu, Xin et al. 1991) |
| Zatelli *et al.*  (2014) |  |  | Only MEN1 screening | (Zatelli, Tagliati et al. 2014) |
| Gierlikowski *et al.*  (2020) |  |  | Only shallow gene panel | (Gierlikowski, Skwarek-Szewczyk et al. 2020) |
| Woischke *et al.*  (2021) | No G1/G2 PNET |  |  | (Woischke, Jung et al. 2021) |
| Pardi *et al.*  (2015) |  |  | Only CDKN1B screening | (Pardi, Mariotti et al. 2015) |
| Cohen *et al.*  (1983) |  | No WES or WGS |  | (Cohen, Feiner et al. 1983) |
| Bocchini *et al.*  (2023) |  | No WES or WGS |  | (Bocchini, Tazzari et al. 2023) |
| Beckert *et al.*  (2020) | No PNET |  |  | (Beckert, Sanchez-Padilla et al. 2020) |
|  |  |  |  |  |
| Depoilly *et al.*  (2022) |  |  | Only ATRX and DAXX screening | (Depoilly, Leroux et al. 2022) |
| Hauser *et al.*  (2019) |  | No WES or WGS |  | (Hauser, Gerson et al. 2019) |
| Ling *et al.*  (2022) |  |  | Only germline variant identification | (Ling, Hong et al. 2022) |
| Zhang *et al.*  (2013) |  | A review without new data |  | (Zhang, Francois et al. 2013) |
| Singhi *et al.*  (2017) |  | No WES or WGS |  | (Singhi, Liu et al. 2017) |
| Lin *et al.*  (2015) |  | No WES or WGS |  | (Lin, Watanabe et al. 2015) |
| Remon *et al.*  (2019) | No PNET |  |  | (Remon, Lacroix et al. 2019) |
| Liu *et al.*  (2022) | No PNET |  |  | (Liu, Dhanda et al. 2022) |
| Liu *et al.*  (2023) | No PNET |  |  | (Liu, Zhu et al. 2023) |
| Perrier (2018) |  | No WES or WGS, only review |  | (Perrier 2018) |
| Zhang *et al.*  (2022) | No PNET |  |  | (Zhang, Chen et al. 2022) |
| Almehmadi *et al.*  (2021) | No PNET |  |  | (Mohammed Almehmadi, Saleh Dairi et al. 2021) |
| Maxwell *et al.*  (2016) |  | No WES or WGS |  | (Maxwell, Sherman et al. 2016) |
| Zhu *et al.*  (2018) | No PNET |  |  | (Zhu, Liu et al. 2018) |
| Niehusmann *et al.*  (2022) | No PNET |  |  | (Niehusmann, Stensvold et al. 2022) |
| Choi *et al.*  (2000) | No PNET |  |  | (Choi, Kong et al. 2000) |
| Wong *et al.*  (2018) | Only WGS of metastases |  |  | (Wong, Yang et al. 2018) |
| Stevenson *et al.*  (2018) |  | No WES or WGS, only a review |  | (Stevenson, Lines et al. 2018) |
| Korshunov *et al.*  (2021) | No PNET |  |  | (Korshunov, Okonechnikov et al. 2021) |
| Mühlisch *et al.*  (2006) | No PNET |  |  | (Mühlisch, Schwering et al. 2006) |
| He *et al.*  (2022) | No PNET |  |  | (He, Song et al. 2022) |
| Kubota *et al.*  (2015) |  |  | Only CTNN1B screening | (Kubota, Kawakami et al. 2015) |
| Dinter *et al.*  (2019) | No PNET |  |  | (Dinter, Bohnenberger et al. 2019) |
| Sasaki *et al.*  (2019) | No PNET |  |  | (Sasaki, Tomomasa et al. 2019) |
| Cros *et al.*  (2016) |  |  | Only FGFR4 screening | (Cros, Moati et al. 2016) |
| Vashist *et al.*  (2011) |  | Only identifying repeat polymorphism |  | (Vashist, Uzunoglu et al. 2011) |
| von Eckardstein *et al.*  (1997) | No PNET |  |  | (von Eckardstein, Gries et al. 1997) |
| Ida *et al.*  (1995) | No PNET |  |  | (Ida, Kobayashi et al. 1995) |
| Zhou *et al.*  (2023) |  |  | 341 genes in gene panel | (Zhou, Han et al. 2023) |
| Lines *et al.*  (2017) |  | No WES or WGS |  | (Lines, Stevenson et al. 2017) |
| Ho *et al.*  (2015) | No PNET |  |  | (Ho, Shih et al. 2015) |
| Wyvekens *et al.*  (2022) | No PNET |  |  | (Wyvekens, Sholl et al. 2022) |
| Gao *et al.*  (2018) | No PNET |  |  | (Gao, Feng et al. 2018) |
| Mellai *et al.*  (2011) | No PNET |  |  | (Mellai, Piazzi et al. 2011) |
| Radig *et al.*  (1998) | No PNET |  |  | (Radig, Schneider-Stock et al. 1998) |
| Xu *et al.*  (2019) | No PNET |  |  | (Xu, Zheng et al. 2019) |
| Peng *et al.*  (2022) | No PNET |  |  | (Peng, Cao et al. 2022) |
| Zhang *et al.*  (2016) | No PNET |  |  | (Zhang, Zhang et al. 2016) |
| Li *et al.*  (2020) | No PNET |  |  | (Li, Vasiljevic et al. 2020) |
| Grigoriu *et al.*  (2021) | No PNET |  |  | (Grigoriu, Terzea et al. 2021) |
| Rabinowits *et al.*  (2017) | No PNET |  |  | (Rabinowits, Barletta et al. 2017) |
| El-Ayadi *et al.*  (2018) | No PNET |  |  | (El-Ayadi, Egervari et al. 2018) |
| Unland *et al.*  (2014) | No PNET |  |  | (Unland, Kerl et al. 2014) |
| Koch *et al.*  (2001) | No PNET |  |  | (Koch, Waha et al. 2001) |
| Weisbrod *et al.*  (2013) |  | No WES or WGS |  | (Weisbrod, Zhang et al. 2013) |
| Miller *et al.*  (2013) | No PNET |  |  | (Miller, Ward et al. 2013) |
| Hu *et al.*  (2022) | No PNET |  |  | (Hu, Yu et al. 2022) |
| Libutti *et al.*  (2000) |  |  | Only germline VHL screening | (Libutti, Choyke et al. 2000) |
| Chou *et al.*  (2016) |  |  | Gene panel of 43 genes | (Chou, Lin et al. 2016) |
| Kövesdi *et al.*  (2020) |  | No WES or WGS |  | (Kövesdi, Kurucz et al. 2020) |
| Greco *et al.*  (2015) | No G1/G2 PNET |  |  | (Greco, Lennington et al. 2015) |
| Zurawel *et al.*  (2000) | No PNET |  |  | (Zurawel, Allen et al. 2000) |
| Dong *et al.*  (2015) | No PNET |  |  | (Dong, Liu et al. 2015) |
| Suwala *et al.*  (2021) | No PNET |  |  | (Suwala, Stichel et al. 2021) |
| Frühwald *et al.*  (2000) | No PNET |  |  | (Frühwald, O'Dorisio et al. 2000) |
| Simbolo *et al.*  (2022) | No PNET |  |  | (Simbolo, Centonze et al. 2022) |
| Sumegi *et al.*  (2011) | No PNET |  |  | (Sumegi, Nishio et al. 2011) |
| Gessi *et al.*  (2011) | No PNET |  |  | (Gessi, Setty et al. 2011) |
| van Noesel *et al.*  (2002) | No PNET |  |  | (van Noesel, van Bezouw et al. 2002) |
| Blansfield *et al.*  (2007) |  |  | Only germline screening | (Blansfield, Choyke et al. 2007) |
| Rekhi *et al.*  (2010) | No PNET |  |  | (Rekhi, Basak et al. 2010) |
| Backer *et al.*  (1998) | No PNET |  |  | (Backer, Mount et al. 1998) |
| Okada *et al.*  (2011) | No PNET |  |  | (Okada, Kamata et al. 2011) |
| Febres-Aldana *et al.*  (2020) | No PNET |  |  | (Febres-Aldana, Krishnamurthy et al. 2020) |
| Parija *et al.*  (2005) | No PNET |  |  | (Parija, Shirley et al. 2005) |
| Portwine *et al.*  (2001) | No PNET |  |  | (Portwine, Chilton-MacNeill et al. 2001) |
| Shiratsuchi *et al.*  (2002) | No PNET |  |  | (Shiratsuchi, Saito et al. 2002) |
| Gisder *et al.*  (2023) |  |  | Only DAXX and ATRX screening | (Gisder, Overheu et al. 2023) |
| Noda *et al.*  (2020) |  | No WES or WGS |  | (Noda, Kuroki et al. 2020) |

# Supplementary Table 2. Articles excluded based on data and text after thorough article review.

| **Author** | **Exclusion criteria** | **Reference** |
| --- | --- | --- |
| Anoshkin *et al.*  (2021) | No mutations identified using WES | (Anoshkin, Vasilyev et al. 2021) |
| Hong *et al.*  (2020) | WES data not available | (Hong, Qiao et al. 2020) |
| Skalniak *et al.*  (2023) | Only germline mutations | (Skalniak, Trofimiuk-Müldner et al. 2023) |
| Iacovazzo *et al.*  (2018) | Samples not specified | (Iacovazzo, Flanagan et al. 2018) |
| Fottner *et al.*  (2022) | WES data not available | (Fottner, Sollfrank et al. 2022) |
| Vax *et al.*  (2003) | Sanger sequencing of CDK4 gene | (Vax, Bibi et al. 2003) |
| Rico *et al.*  (2021) | Only MEN1 screening | (Rico, Duan et al. 2021) |
| Boons *et al.*  (2018) | WES data not available | (Boons, Vandamme et al. 2018) |
| Martin *et al.*  (2018) | No mutations identified in low grade PNET | (Martin, LaBauve et al. 2018) |
| Xiao *et al.*  (2023) | Only raw data available and not accessible | (Xiao, Xu et al. 2023) |
| Lichtenauer *et al.*  (2015) | Missing HGVS data | (Lichtenauer, Di Dalmazi et al. 2015) |
| Sakurai *et al.*  (2023) | Missing method section | (Sakurai, Wakabayashi et al. 2023) |
| Tang *et al.*  (2012) | Only isolation of gDNA for copy number variations | (Tang, Contractor et al. 2012) |
| Edil *et al.*  (2020) | Review of the heterogeneity of PNETs and pancreatic ductal adenocarcinomas, respectively | (Edil, Luo et al. 2020) |
| Challis *et al.*  (2017) | Histological grade not specified | (Challis, Powlson et al. 2017) |
| Kidd *et al.*  (2015) | A review of PNET pathobiology | (Kidd, Modlin et al. 2015) |
| Backman *et al.*  (2017) | 22 genes in gene panel and no data on initial sequencing | (Backman, Norlén et al. 2017) |
| Boons *et al.*  (2019) | A review of gastroenteropancreatic NENs | (Boons, Vandamme et al. 2019) |
| Scarpa *et al.*  (2019) | A review of gastroenteropancreatic NENs | (Scarpa 2019) |
| Lee *et al.*  (2016) | Review of personalized medicine in PNETs | (Lee and O'Neil 2016) |
| Pozas *et al.*  (2022) | Review of therapeutic drug targets in PNETs | (Pozas, Alonso-Gordoa et al. 2022) |
| Quevedo *et al.*  (2020) | No defined WHO grade | (Quevedo, Spreafico et al. 2020) |
| Raj *et al.*  (2022) | No G1/G2 PNET | (Raj, Coffman et al. 2022) |
| Puccini *et al.*  (2020) | No individual data points on NGS or patient data | (Puccini, Poorman et al. 2020) |
| Herring *et al.*  (2022) | A review of racial variations in GEP-NENs | (Herring, Bonner et al. 2022) |
| Park *et al.*  (2016) | Panel of 341 genes. No HGVS data. | (Park, Ha et al. 2016) |
| Bodei *et al.*  (2023) | Gene panel of 341 genes. NGS method not described in details. | (Bodei, Raj et al. 2023) |
| Pelosi *et al.*  (2021) | Use data from a study (Vijayvergia, Boland et al. 2016) with a 50-gene panel. | (Pelosi, Bianchi et al. 2021) |
| Zhang *et al.*  (2023) | No HGVS or variant data | (Zhang, Jiang et al. 2023) |
| Di Domenico *et al.*  (2020) | Only MEN1, ATRX and DAXX screening | (Di Domenico, Pipinikas et al. 2020) |
| Roy *et al.*  (2018) | Only exome sequencing of distant metastases | (Roy, LaFramboise et al. 2018) |
| Vandamme *et al.*  (2019) | Gene panel of 20 genes. | (Vandamme, Beyens et al. 2019) |
| Gong *et al.*  (2019) | Method section refers to DNA sequencing of 287 genes (Frampton, Fichtenholtz et al. 2013) | (Gong, Blais et al. 2019) |

Supplementary Figures

**
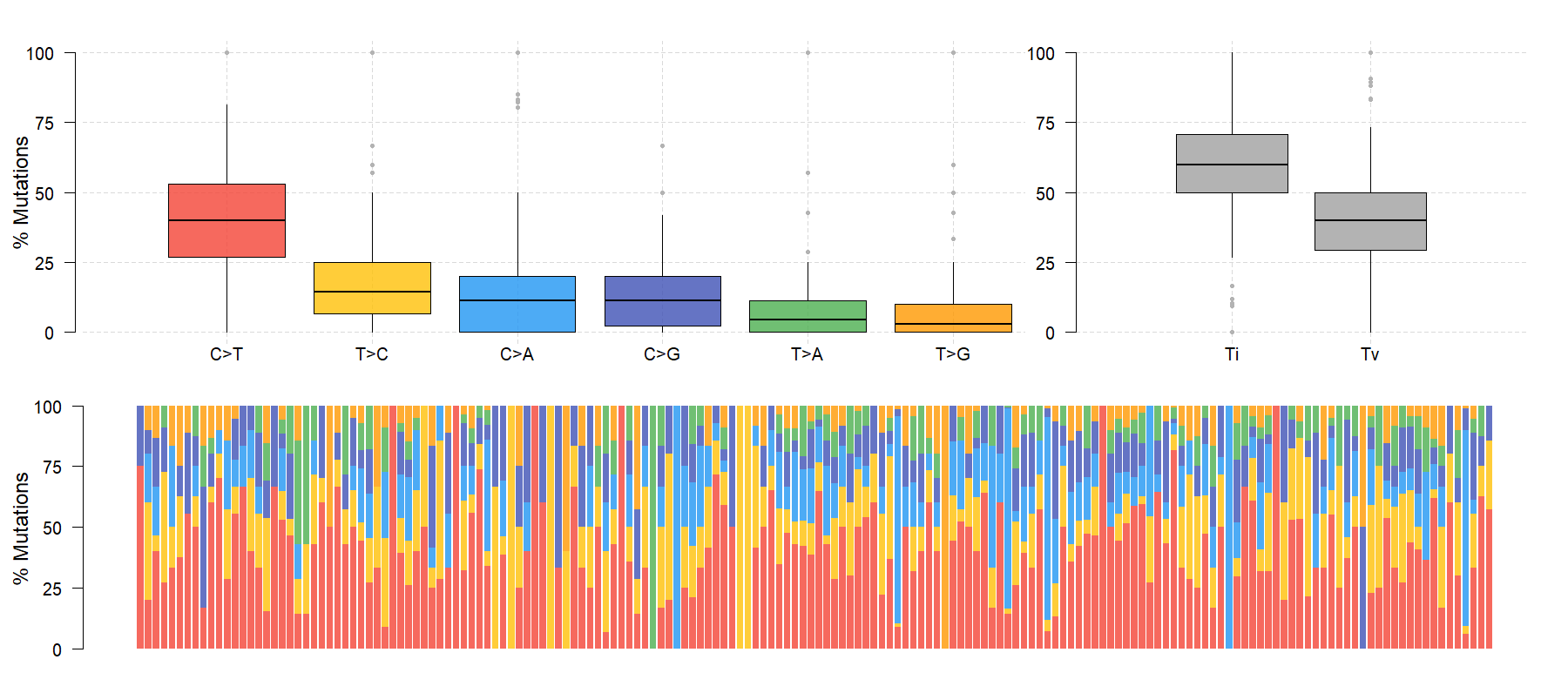
Supplementary Figure 1.**

A summary of variants in the included datasets. Upper row) the distribution of transitions (Ti), i.e. C>T and T>C and transversions (Tv), C>A , C>G, T>A and T>G. Lower row) Overview of Ti and Tv in every pancreatic neuroendocrine tumor in the dataset, represented by each column with the respective color combinations as indicated in the upper row.

**A)**

**
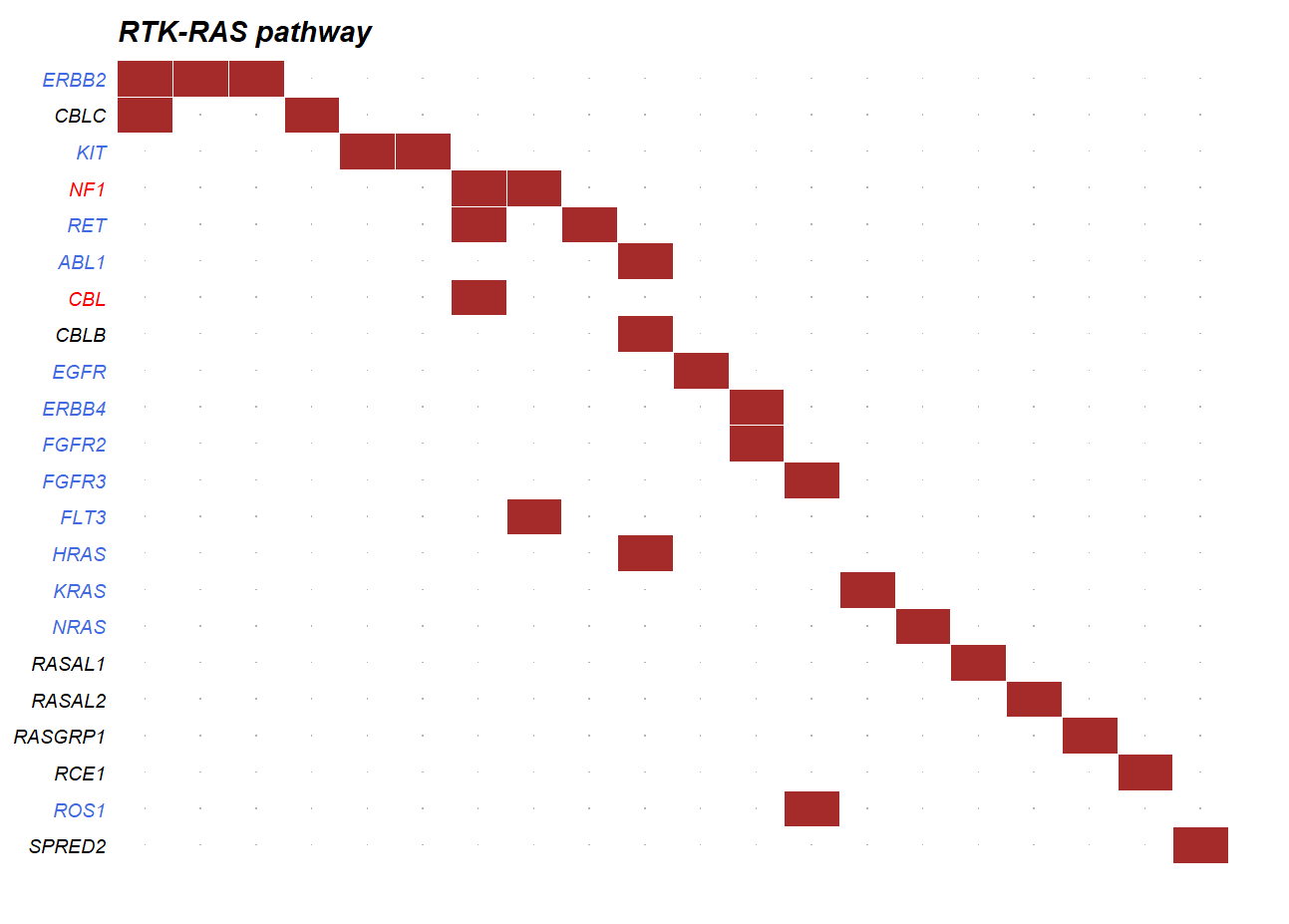
**

**B)**

**
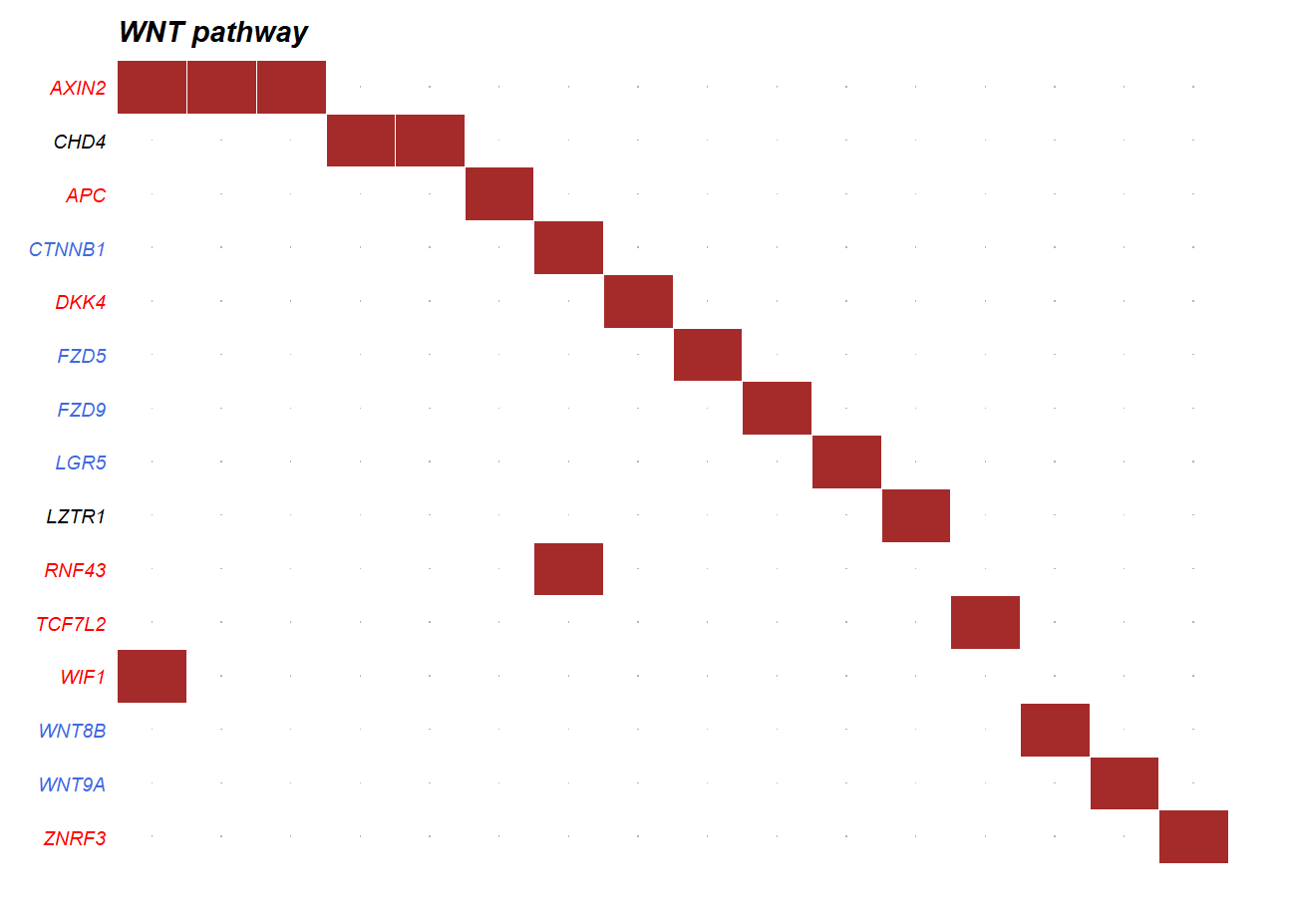
**

**C)**

**
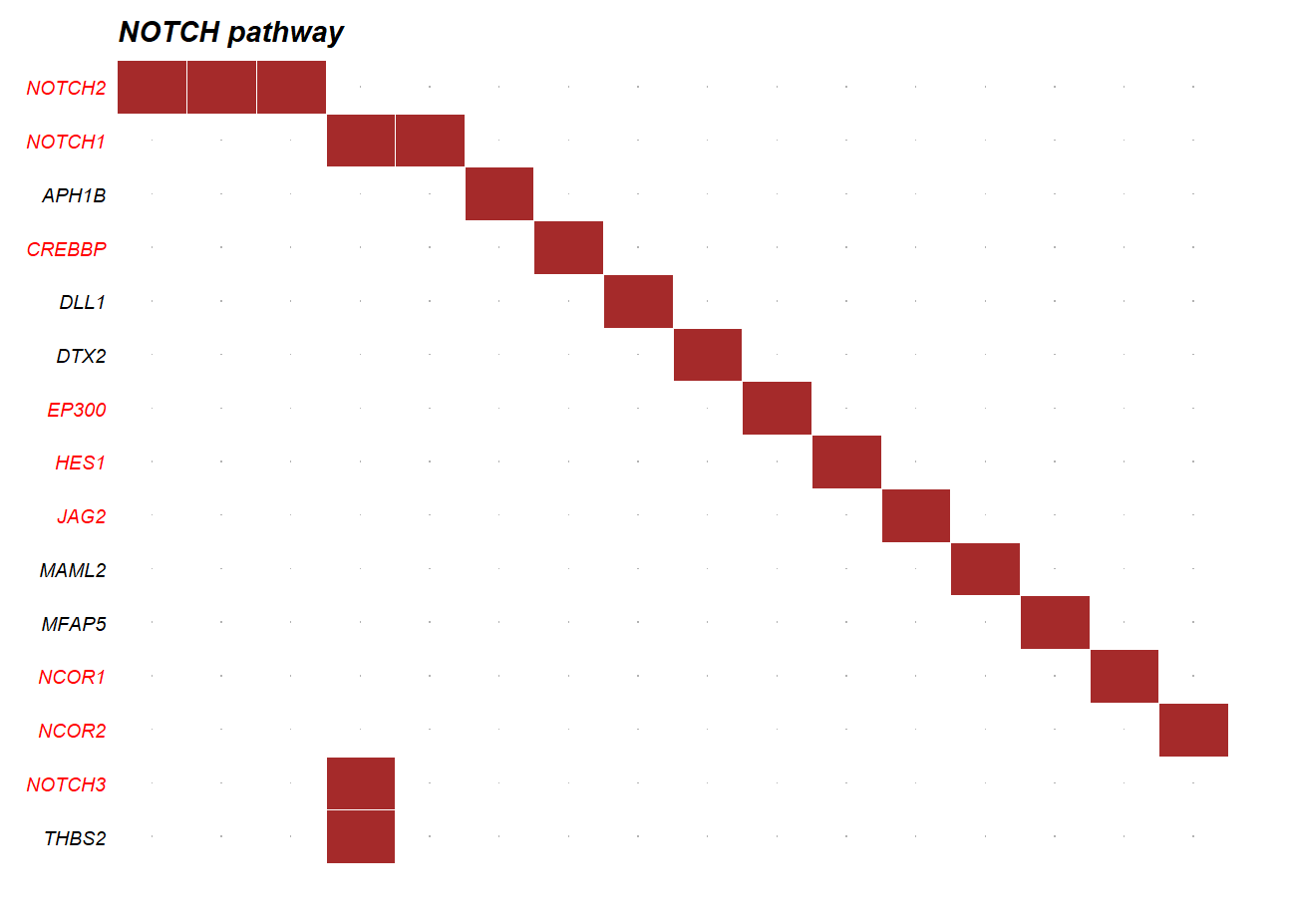
**

**Supplementary Figure 2.** Oncogenic pathways in nonfunctional pancreatic neuroendocrine tumors (PNETs). Top oncogenic pathways in >10% of nonfunctional PNETs, including **(A)** RTK-RAS (n=20), **(B)** Wnt pathway (n=16) and **(C)** NOTCH (n=16). RTK-RAS: Receptor Tyrosine Kinase-Ras signaling pathway.

# Supplementary references

Ackeifi, C., E. Swartz, K. Kumar, H. Liu, S. Chalada, E. Karakose, D. K. Scott, A. Garcia-Ocaña, R. Sanchez, R. J. DeVita, A. F. Stewart and P. Wang (2020). "Pharmacologic and genetic approaches define human pancreatic β cell mitogenic targets of DYRK1A inhibitors." JCI Insight **5**(1).

Alonso-Gordoa, T., J. J. Díez, J. Molina, P. Reguera, O. Martínez-Sáez and E. Grande (2015). "An Overview on the Sequential Treatment of Pancreatic Neuroendocrine Tumors (pNETs)." Rare Cancers Ther **3**(1): 13-33.

Andersson Svärd, A., M. Maziarz, A. Ramelius, M. Lundgren, Å. Lernmark and H. Elding Larsson (2020). "Decreased HLA-DQ expression on peripheral blood cells in children with varying number of beta cell autoantibodies." J Transl Autoimmun **3**: 100052.

Anoshkin, K., I. Vasilyev, K. Karandasheva, M. Shugay, V. Kudryavtseva, A. Egorov, L. Gurevich, A. Mironova, A. Serikov, S. Kutsev and V. Strelnikov (2021). "New Regions With Molecular Alterations in a Rare Case of Insulinomatosis: Case Report With Literature Review." Front Endocrinol (Lausanne) **12**: 760154.

Archetti, M., D. A. Ferraro and G. Christofori (2015). "Heterogeneity for IGF-II production maintained by public goods dynamics in neuroendocrine pancreatic cancer." Proc Natl Acad Sci U S A **112**(6): 1833-1838.

Backer, A., S. L. Mount, M. A. Zarka, C. E. Trask, E. F. Allen, W. L. Gerald, D. A. Sanders and D. L. Weaver (1998). "Desmoplastic small round cell tumour of unknown primary origin with lymph node and lung metastases: histological, cytological, ultrastructural, cytogenetic and molecular findings." Virchows Arch **432**(2): 135-141.

Backman, S., O. Norlén, B. Eriksson, B. Skogseid, P. Stålberg and J. Crona (2017). "Detection of Somatic Mutations in Gastroenteropancreatic Neuroendocrine Tumors Using Targeted Deep Sequencing." Anticancer Res **37**(2): 705-712.

Balogh, K., A. Patócs, J. Majnik, F. Varga, G. Illyés, L. Hunyady and K. Rácz (2004). "Unusual presentation of multiple endocrine neoplasia type 1 in a young woman with a novel mutation of the MEN1 gene." J Hum Genet **49**(7): 380-386.

Baran-Gale, J., E. E. Fannin, C. L. Kurtz and P. Sethupathy (2013). "Beta cell 5'-shifted isomiRs are candidate regulatory hubs in type 2 diabetes." PLoS One **8**(9): e73240.

Beck, R., M. Chandi, M. Kanke, M. Stýblo and P. Sethupathy (2019). "Arsenic is more potent than cadmium or manganese in disrupting the INS-1 beta cell microRNA landscape." Arch Toxicol **93**(11): 3099-3109.

Beckert, P., E. Sanchez-Padilla, M. Merker, V. Dreyer, T. A. Kohl, C. Utpatel, C. U. Köser, I. Barilar, N. Ismail, S. V. Omar, M. Klopper, R. M. Warren, H. Hoffmann, G. Maphalala, E. Ardizzoni, B. C. de Jong, B. Kerschberger, B. Schramm, S. Andres, K. Kranzer, F. P. Maurer, M. Bonnet and S. Niemann (2020). "MDR M. tuberculosis outbreak clone in Eswatini missed by Xpert has elevated bedaquiline resistance dated to the pre-treatment era." Genome Med **12**(1): 104.

Birla, S., P. J. V, R. Singla, M. Tripathi and A. Sharma (2015). "Impact of a novel 14 bp MEN1 deletion in a patient with hyperparathyroidism and gastrinoma." Endocrinol Diabetes Metab Case Rep **2015**: 150011.

Blansfield, J. A., L. Choyke, S. Y. Morita, P. L. Choyke, J. F. Pingpank, H. R. Alexander, G. Seidel, Y. Shutack, N. Yuldasheva, M. Eugeni, D. L. Bartlett, G. M. Glenn, L. Middelton, W. M. Linehan and S. K. Libutti (2007). "Clinical, genetic and radiographic analysis of 108 patients with von Hippel-Lindau disease (VHL) manifested by pancreatic neuroendocrine neoplasms (PNETs)." Surgery **142**(6): 814-818; discussion 818.e811-812.

Bocchini, M., M. Tazzari, S. Ravaioli, F. Piccinini, F. Foca, M. Tebaldi, F. Nicolini, I. Grassi, S. Severi, R. A. Calogero, M. Arigoni, J. Schrader, M. Mazza and G. Paganelli (2023). "Circulating hsa-miR-5096 predicts (18)F-FDG PET/CT positivity and modulates somatostatin receptor 2 expression: a novel miR-based assay for pancreatic neuroendocrine tumors." Front Oncol **13**: 1136331.

Bodei, L., N. Raj, R. K. Do, A. Mauguen, S. Krebs, D. Reidy-Lagunes and H. Schöder (2023). "Interim Analysis of a Prospective Validation of 2 Blood-Based Genomic Assessments (PPQ and NETest) to Determine the Clinical Efficacy of (177)Lu-DOTATATE in Neuroendocrine Tumors." J Nucl Med **64**(4): 567-573.

Boons, G., T. Vandamme, L. Mariën, W. Lybaert, G. Roeyen, T. Rondou, K. Papadimitriou, K. Janssens, B. Op de Beeck, M. Simoens, W. Demey, I. Dero, G. Van Camp, M. Peeters and K. Op de Beeck (2022). "Longitudinal Copy-Number Alteration Analysis in Plasma Cell-Free DNA of Neuroendocrine Neoplasms is a Novel Specific Biomarker for Diagnosis, Prognosis, and Follow-up." Clin Cancer Res **28**(2): 338-349.

Boons, G., T. Vandamme, M. Peeters, M. Beyens, A. Driessen, K. Janssens, K. Zwaenepoel, G. Roeyen, G. Van Camp and K. Op de Beeck (2018). "Cell-Free DNA From Metastatic Pancreatic Neuroendocrine Tumor Patients Contains Tumor-Specific Mutations and Copy Number Variations." Front Oncol **8**: 467.

Boons, G., T. Vandamme, M. Peeters, G. Van Camp and K. Op de Beeck (2019). "Clinical applications of (epi)genetics in gastroenteropancreatic neuroendocrine neoplasms: Moving towards liquid biopsies." Rev Endocr Metab Disord **20**(3): 333-351.

Boora, G. K., R. Kanwar, A. A. Kulkarni, J. Pleticha, M. Ames, G. Schroth, A. S. Beutler and M. S. Banck (2015). "Exome-level comparison of primary well-differentiated neuroendocrine tumors and their cell lines." Cancer Genet **208**(7-8): 374-381.

Boratto, S. D. F., P. A. S. Cardoso, D. G. Priolli, R. V. Botelho, A. Goldenberg, B. Bianco and J. Waisberg (2020). "von Hippel-Lindau Syndrome: Genetic Study of Case With a Rare Pathogenic Variant With Optic Nerve Hemangioblastoma, a Rare Phenotypic Expression." Front Oncol **10**: 139.

Bristulf, J., S. Gatti, D. Malinowsky, L. Bjork, A. K. Sundgren and T. Bartfai (1994). "Interleukin-1 stimulates the expression of type I and type II interleukin-1 receptors in the rat insulinoma cell line Rinm5F; sequencing a rat type II interleukin-1 receptor cDNA." Eur Cytokine Netw **5**(3): 319-330.

Busico, A., P. Maisonneuve, N. Prinzi, S. Pusceddu, G. Centonze, G. Garzone, A. Pellegrinelli, L. Giacomelli, A. Mangogna, C. Paolino, A. Belfiore, K. Kankava, F. Perrone, E. Tamborini, G. Pruneri, N. Fazio and M. Milione (2020). "Gastroenteropancreatic High-Grade Neuroendocrine Neoplasms: Histology and Molecular Analysis, Two Sides of the Same Coin." Neuroendocrinology **110**(7-8): 616-629.

Capodanno, Y., F. O. Buishand, L. Y. Pang, J. Kirpensteijn, J. A. Mol, R. Elders and D. J. Argyle (2020). "Transcriptomic analysis by RNA sequencing characterises malignant progression of canine insulinoma from normal tissue to metastatic disease." Sci Rep **10**(1): 11581.

Carlsson, A., M. Shepherd, S. Ellard, M. Weedon, Å. Lernmark, G. Forsander, K. Colclough, Q. Brahimi, C. Valtonen-Andre, S. A. Ivarsson, H. Elding Larsson, U. Samuelsson, E. Örtqvist, L. Groop, J. Ludvigsson, C. Marcus and A. T. Hattersley (2020). "Absence of Islet Autoantibodies and Modestly Raised Glucose Values at Diabetes Diagnosis Should Lead to Testing for MODY: Lessons From a 5-Year Pediatric Swedish National Cohort Study." Diabetes Care **43**(1): 82-89.

Challis, B. G., J. Harris, A. Sleigh, I. Isaac, S. M. Orme, N. Seevaratnam, K. Dhatariya, H. L. Simpson and R. K. Semple (2014). "Familial adult onset hyperinsulinism due to an activating glucokinase mutation: implications for pharmacological glucokinase activation." Clin Endocrinol (Oxf) **81**(6): 855-861.

Challis, B. G., A. S. Powlson, R. T. Casey, C. Pearson, B. Y. Lam, M. Ma, D. Pitfield, G. S. H. Yeo, E. Godfrey, H. K. Cheow, V. K. Chatterjee, N. R. Carroll, A. Shaw, J. R. Buscombe and H. L. Simpson (2017). "Adult-onset hyperinsulinaemic hypoglycaemia in clinical practice: diagnosis, aetiology and management." Endocr Connect **6**(7): 540-548.

Chatani, P. D., S. K. Agarwal and S. M. Sadowski (2020). "Molecular Signatures and Their Clinical Utility in Pancreatic Neuroendocrine Tumors." Front Endocrinol (Lausanne) **11**: 575620.

Choi, J. H. and W. H. Paik (2022). "Risk Stratification of Pancreatic Neuroendocrine Neoplasms Based on Clinical, Pathological, and Molecular Characteristics." J Clin Med **11**(24).

Choi, S. H., X. T. Kong, T. Taki, Y. Tsuchida, H. Kawaguchi, H. Kato, R. Hanada, A. T. Look and Y. Hayashi (2000). "Reduced or absent expression and codon 201Gly/Arg polymorphism of DCC gene in rhabdomyosarcoma and Ewing's sarcoma/PNET family." Int J Mol Med **6**(4): 463-467.

Chou, W. C., P. H. Lin, Y. C. Yeh, Y. M. Shyr, W. L. Fang, S. E. Wang, C. Y. Liu, P. M. Chang, M. H. Chen, Y. P. Hung, C. P. Li, Y. Chao and M. H. Chen (2016). "Genes involved in angiogenesis and mTOR pathways are frequently mutated in Asian patients with pancreatic neuroendocrine tumors." Int J Biol Sci **12**(12): 1523-1532.

Cinque, L., A. Sparaneo, F. Cetani, M. Coco, C. Clemente, M. Chetta, T. Balsamo, C. Battista, E. Sanpaolo, E. Pardi, L. D'Agruma, C. Marcocci, E. Maiello, G. N. Hendy, D. E. C. Cole, A. Scillitani and V. Guarnieri (2017). "Novel association of MEN1 gene mutations with parathyroid carcinoma." Oncol Lett **14**(1): 23-30.

Cives, M., V. Simone, F. M. Rizzo and F. Silvestris (2016). "NETs: organ-related epigenetic derangements and potential clinical applications." Oncotarget **7**(35): 57414-57429.

Cloyd, J. M., A. Ejaz, B. Konda, M. S. Makary and T. M. Pawlik (2020). "Neuroendocrine liver metastases: a contemporary review of treatment strategies." Hepatobiliary Surg Nutr **9**(4): 440-451.

Cloyd, J. M., J. T. Wiseman and T. M. Pawlik (2020). "Surgical management of pancreatic neuroendocrine liver metastases." J Gastrointest Oncol **11**(3): 590-600.

Cohen, D. H., H. Feiner, O. Jensson and B. Frangione (1983). "Amyloid fibril in hereditary cerebral hemorrhage with amyloidosis (HCHWA) is related to the gastroentero-pancreatic neuroendocrine protein, gamma trace." J Exp Med **158**(2): 623-628.

Cram, D. S., A. McIntosh, L. Oxbrow, A. M. Johnston and H. J. DeAizpurua (1999). "Differential mRNA display analysis of two related but functionally distinct rat insulinoma (RIN) cell lines: identification of CD24 and its expression in the developing pancreas." Differentiation **64**(4): 237-246.

Cromer, M. K., M. Choi, C. Nelson-Williams, A. L. Fonseca, J. W. Kunstman, R. M. Korah, J. D. Overton, S. Mane, B. Kenney, C. D. Malchoff, P. Stalberg, G. Akerstrom, G. Westin, P. Hellman, T. Carling, P. Bjorklund and R. P. Lifton (2015). "Neomorphic effects of recurrent somatic mutations in Yin Yang 1 in insulin-producing adenomas." Proc Natl Acad Sci U S A **112**(13): 4062-4067.

Cros, J., E. Moati, J. Raffenne, O. Hentic, M. Svrcek, L. de Mestier, E. Sbidian, N. Guedj, P. Bedossa, V. Paradis, A. Sauvanet, Y. Panis, P. Ruszniewski, A. Couvelard and P. Hammel (2016). "Gly388Arg FGFR4 Polymorphism Is Not Predictive of Everolimus Efficacy in Well-Differentiated Digestive Neuroendocrine Tumors." Neuroendocrinology **103**(5): 495-499.

Cuyle, P. J. and H. Prenen (2018). "Practical management of toxicities associated with targeted therapies for advanced gastroenteropancreatic neuroendocrine tumors." Ann Gastroenterol **31**(2): 140-150.

Demirtaş, C., P. Ata, A. Çetin, A. Türkyılmaz and D. G. Duman (2020). "A large Turkish pedigree with multiple endocrine neoplasia type 1 syndrome carrying a rare mutation: c.1680_1683 del TGAG." Turk J Gastroenterol **31**(7): 508-514.

Depoilly, T., R. Leroux, D. Andrade, R. Nicolle, M. Dioguardi Burgio, I. Marinoni, S. Dokmak, P. Ruszniewski, O. Hentic, V. Paradis, L. De Mestier, A. Perren, A. Couvelard and J. Cros (2022). "Immunophenotypic and molecular characterization of pancreatic neuroendocrine tumors producing serotonin." Modern Pathology **35**(11): 1713-1722.

Desai, S. S., S. S. Kharade, V. I. Parekh, S. Iyer and S. K. Agarwal (2015). "Pro-oncogenic Roles of HLXB9 Protein in Insulinoma Cells through Interaction with Nono Protein and Down-regulation of the c-Met Inhibitor Cblb (Casitas B-lineage Lymphoma b)." J Biol Chem **290**(42): 25595-25608.

Di Domenico, A., C. P. Pipinikas, R. S. Maire, K. Bräutigam, C. Simillion, M. S. Dettmer, E. Vassella, C. Thirlwell, A. Perren and I. Marinoni (2020). "Epigenetic landscape of pancreatic neuroendocrine tumours reveals distinct cells of origin and means of tumour progression." Commun Biol **3**(1): 740.

Dijkstra, K. K., J. G. van den Berg, F. Weeber, J. van de Haar, A. Velds, S. Kaing, D. Peters, F. Eskens, D. A. de Groot, M. E. T. Tesselaar and E. E. Voest (2021). "Patient-Derived Organoid Models of Human Neuroendocrine Carcinoma." Front Endocrinol (Lausanne) **12**: 627819.

Ding, W. Q., Z. J. Cheng, J. McElhiney, S. M. Kuntz and L. J. Miller (2002). "Silencing of secretin receptor function by dimerization with a misspliced variant secretin receptor in ductal pancreatic adenocarcinoma." Cancer Res **62**(18): 5223-5229.

Dinter, H., H. Bohnenberger, J. Beck, K. Bornemann-Kolatzki, E. Schütz, S. Küffer, L. Klein, T. J. Franks, A. Roden, A. Emmert, M. Hinterthaner, M. Marino, L. Brcic, H. Popper, C. A. Weis, G. Pelosi, A. Marx and P. Ströbel (2019). "Molecular Classification of Neuroendocrine Tumors of the Thymus." J Thorac Oncol **14**(8): 1472-1483.

Dizdar, L., T. A. Werner, J. C. Drusenheimer, B. Möhlendick, K. Raba, I. Boeck, M. Anlauf, M. Schott, W. Göring, I. Esposito, N. H. Stoecklein, W. T. Knoefel and A. Krieg (2019). "BRAF(V600E) mutation: A promising target in colorectal neuroendocrine carcinoma." Int J Cancer **144**(6): 1379-1390.

Donadel, G., N. Marinos, M. G. DeSilva, J. Lu, A. L. Notkins and M. S. Lan (1998). "Molecular cloning and characterization of a highly basic protein, IA-4, expressed in pancreatic islets and brain." Neuroendocrinology **67**(3): 190-196.

Dong, M., J. Liu, Z. Song, X. Li, T. Shi, D. Wang, D. Ren and J. Chen (2015). "Primary Multiple Pulmonary Primitive Neuroectodermal Tumor: Case Report and Literature Review." Medicine (Baltimore) **94**(27): e1136.

Durinovic-Bellò, I., A. Steinle, A. G. Ziegler and D. J. Schendel (1994). "HLA-DQ-restricted, islet-specific T-cell clones of a type I diabetic patient. T-cell receptor sequence similarities to insulitis-inducing T-cells of nonobese diabetic mice." Diabetes **43**(11): 1318-1325.

Durning, S. P., H. Flanagan-Steet, N. Prasad and L. Wells (2016). "O-Linked β-N-acetylglucosamine (O-GlcNAc) Acts as a Glucose Sensor to Epigenetically Regulate the Insulin Gene in Pancreatic Beta Cells." J Biol Chem **291**(5): 2107-2118.

Ediger, B. N., A. Du, J. Liu, C. S. Hunter, E. R. Walp, J. Schug, K. H. Kaestner, R. Stein, D. A. Stoffers and C. L. May (2014). "Islet-1 Is essential for pancreatic β-cell function." Diabetes **63**(12): 4206-4217.

Edil, B. H., W. Luo and M. Li (2020). "Genomic and single cell sequencing facilitate the dissection of heterogeneity of pancreatic tumors." BMC Medicine **18**(1): 177.

El-Ayadi, M., K. Egervari, D. Merkler, T. A. McKee, F. Gumy-Pause, D. Stichel, D. Capper, T. Pietsch, M. Ansari and A. O. von Bueren (2018). "Concurrent IDH1 and SMARCB1 Mutations in Pediatric Medulloblastoma: A Case Report." Front Neurol **9**: 398.

Evers, B. M., P. L. Rady, K. Sandoval, I. Arany, S. K. Tyring, R. L. Sanchez, W. H. Nealon, C. M. Townsend, Jr. and J. C. Thompson (1994). "Gastrinomas demonstrate amplification of the HER-2/neu proto-oncogene." Ann Surg **219**(6): 596-601; discussion 602-594.

Febres-Aldana, C. A., K. Krishnamurthy, R. Delgado, J. Kochiyil, R. Poppiti and A. M. Medina (2020). "Prostatic carcinoma with neuroendocrine differentiation harboring the EWSR1-FEV fusion transcript in a man with the WRN G327X germline mutation: A new variant of prostatic carcinoma or a member of the Ewing sarcoma family of tumors?" Pathol Res Pract **216**(2): 152758.

Finnerty, B. M., M. D. Moore, A. Verma, A. Aronova, S. Huang, D. P. Edwards, Z. Chen, M. Seandel, T. Scognamiglio, Y. N. Du, O. Elemento, R. Zarnegar, I. M. Min and T. J. Fahey (2019). "UCHL1 loss alters the cell-cycle in metastatic pancreatic neuroendocrine tumors." Endocr Relat Cancer **26**(4): 411-423.

Flaum, N., J. W. Valle, W. Mansoor and M. G. McNamara (2016). "Everolimus in the treatment of neuroendocrine tumors of the respiratory and gastroenteropancreatic systems." Future Oncol **12**(22): 2561-2578.

Fortunati, E., N. Bonazzi, L. Zanoni, S. Fanti and V. Ambrosini (2023). "Molecular imaging Theranostics of Neuroendocrine Tumors." Semin Nucl Med **53**(4): 539-554.

Fottner, C., S. Sollfrank, M. Ghiasi, A. Adenaeuer, T. Musholt, A. Schad, M. Miederer, S. Schadmand-Fischer, M. M. Weber, K. J. Lackner and H. Rossmann (2022). "Second MAFA Variant Causing a Phosphorylation Defect in the Transactivation Domain and Familial Insulinomatosis." Cancers (Basel) **14**(7).

Frampton, G. M., A. Fichtenholtz, G. A. Otto, K. Wang, S. R. Downing, J. He, M. Schnall-Levin, J. White, E. M. Sanford, P. An, J. Sun, F. Juhn, K. Brennan, K. Iwanik, A. Maillet, J. Buell, E. White, M. Zhao, S. Balasubramanian, S. Terzic, T. Richards, V. Banning, L. Garcia, K. Mahoney, Z. Zwirko, A. Donahue, H. Beltran, J. M. Mosquera, M. A. Rubin, S. Dogan, C. V. Hedvat, M. F. Berger, L. Pusztai, M. Lechner, C. Boshoff, M. Jarosz, C. Vietz, A. Parker, V. A. Miller, J. S. Ross, J. Curran, M. T. Cronin, P. J. Stephens, D. Lipson and R. Yelensky (2013). "Development and validation of a clinical cancer genomic profiling test based on massively parallel DNA sequencing." Nat Biotechnol **31**(11): 1023-1031.

Frühwald, M. C., M. S. O'Dorisio, L. J. Rush, J. L. Reiter, D. J. Smiraglia, G. Wenger, J. F. Costello, P. S. White, R. Krahe, G. M. Brodeur and C. Plass (2000). "Gene amplification in PNETs/medulloblastomas: mapping of a novel amplified gene within the MYCN amplicon." J Med Genet **37**(7): 501-509.

Gajate, P., O. Martínez-Sáez, T. Alonso-Gordoa and E. Grande (2017). "Emerging use of everolimus in the treatment of neuroendocrine tumors." Cancer Manag Res **9**: 215-224.

Gao, Y., Y. Y. Feng, J. H. Yu, Q. C. Li, X. S. Qiu and E. H. Wang (2018). "Diffuse midline gliomas with histone H3-K27M mutation: A rare case with PNET-like appearance and neuropil-like islands." Neuropathology **38**(2): 165-170.

Gerard, L., J. Garcia, A. Gauthier, J. Lopez, A. Durand, V. Hervieu, A. Lemelin, L. Chardon, V. Landel, B. Gibert, C. Lombard-Bohas, L. Payen and T. Walter (2021). "ctDNA in Neuroendocrine Carcinoma of Gastroenteropancreatic Origin or of Unknown Primary: The CIRCAN-NEC Pilot Study." Neuroendocrinology **111**(10): 951-964.

Gessi, M., P. Setty, M. Bisceglia, A. zur Muehlen, L. Lauriola, A. Waha, F. Giangaspero and T. Pietsch (2011). "Supratentorial primitive neuroectodermal tumors of the central nervous system in adults: molecular and histopathologic analysis of 12 cases." Am J Surg Pathol **35**(4): 573-582.

Giacché, M., A. Panarotto, L. Mori, L. Daffini, M. C. Tacchetti, I. Pirola, E. Agabiti Rosei and M. Castellano (2012). "A novel menin gene deletional mutation in a little series of Italian patients affected by apparently sporadic multiple endocrine neoplasia type 1 syndrome." J Endocrinol Invest **35**(2): 124-128.

Gierlikowski, W., A. Skwarek-Szewczyk and M. Popow (2020). "A Novel Germline c.1267T>A MEN1 Mutation in MEN1 Family-from Phenotype to Gene and Back." Genes (Basel) **11**(11).

Gisder, D. M., O. Overheu, J. Keller, S. Nöpel-Dünnebacke, W. Uhl, A. Reinacher-Schick, A. Tannapfel and I. Tischoff (2023). "DAXX, ATRX, and MSI in PanNET and Their Metastases: Correlation with Histopathological Data and Prognosis." Pathobiology **90**(2): 71-80.

Gleeson, F. C., J. S. Voss, B. R. Kipp, S. E. Kerr, J. S. Van Arnam, J. R. Mills, C. A. Marcou, A. R. Schneider, Z. J. Tu, M. R. Henry and M. J. Levy (2017). "Assessment of pancreatic neuroendocrine tumor cytologic genotype diversity to guide personalized medicine using a custom gastroenteropancreatic next-generation sequencing panel." Oncotarget **8**(55): 93464-93475.

Gong, J., E. M. Blais, J. R. Bender, M. Guan, V. Placencio-Hickok, E. F. Petricoin, M. J. Pishvaian, G. Gregory, R. Tuli and A. E. Hendifar (2019). "Multiplatform profiling of pancreatic neuroendocrine tumors: Correlative analyses of clinicopathologic factors and identification of co-occurring pathogenic alterations." Oncotarget **10**(58): 6260-6268.

Greco, F. A., W. J. Lennington, D. R. Spigel and J. D. Hainsworth (2015). "Poorly differentiated neoplasms of unknown primary site: diagnostic usefulness of a molecular cancer classifier assay." Mol Diagn Ther **19**(2): 91-97.

Grigoriu, C., D. C. Terzea, A. C. Lisievici, T. A. Georgescu, A. E. Constantin, N. Bacalbaşa, I. Ducu and R. E. Bohîlţea (2021). "Peripheral-type primitive neuroectodermal tumor of the ovary with EWSR1-FLI1 fusion transcript: a case report and brief review of literature." Rom J Morphol Embryol **62**(2): 581-586.

Hackeng, W. M., R. H. Hruban, G. J. Offerhaus and L. A. Brosens (2016). "Surgical and molecular pathology of pancreatic neoplasms." Diagn Pathol **11**(1): 47.

Hai, N., G. Muto, H. Okamoto, A. Tamada, R. Abe, S. Suzuki and S. Kosugi (2001). "A novel germline mutation of the MEN1 gene, L259del, in a patient with sporadic multiple endocrine neoplasia type 1 (MEN1)." Jpn J Clin Oncol **31**(3): 125-127.

Hameed, S., S. Ellard, H. J. Woodhead, K. A. Neville, J. L. Walker, M. E. Craig, T. Armstrong, L. Yu, G. S. Eisenbarth, A. T. Hattersley and C. F. Verge (2011). "Persistently autoantibody negative (PAN) type 1 diabetes mellitus in children." Pediatr Diabetes **12**(3 Pt 1): 142-149.

Hasani-Ranjbar, S., M. M. Amoli, A. Ebrahim-Habibi, M. H. Gozashti, N. Khalili, F. A. Sayyahpour, J. Hafeziyeh, A. Soltani and B. Larijani (2011). "A new frameshift MEN1 gene mutation associated with familial malignant insulinomas." Fam Cancer **10**(2): 343-348.

Hauser, H., D. S. Gerson, D. Reidy-Lagunes and N. Raj (2019). "Systemic Therapies for Metastatic Pancreatic Neuroendocrine Tumors." Curr Treat Options Oncol **20**(12): 87.

He, X., S. Song, P. Yang, F. Cao, W. Li and P. Liang (2022). "A rare radiotherapy-sensitive primitive neuroectodermal tumor with APC gene amplification in an adult: a case report and literature review." J Int Med Res **50**(8): 3000605221118704.

Herring, B. R., A. Bonner, R. E. Guenter, S. Vickers, C. Yates, G. Lee, D. Dhall, H. Chen and J. B. Rose (2022). "Under-Representation of Racial Groups in Genomics Studies of Gastroenteropancreatic Neuroendocrine Neoplasms." Cancer Res Commun **2**(10): 1162-1173.

Ho, D. M., C. C. Shih, M. L. Liang, C. Y. Tsai, T. H. Hsieh, C. H. Tsai, S. C. Lin, T. Y. Chang, M. E. Chao, H. W. Wang and T. T. Wong (2015). "Integrated genomics has identified a new AT/RT-like yet INI1-positive brain tumor subtype among primary pediatric embryonal tumors." BMC Med Genomics **8**: 32.

Hofving, T., Y. Arvidsson, B. Almobarak, L. Inge, R. Pfragner, M. Persson, G. Stenman, E. Kristiansson, V. Johanson and O. Nilsson (2018). "The neuroendocrine phenotype, genomic profile and therapeutic sensitivity of GEPNET cell lines." Endocr Relat Cancer **25**(3): 367-380.

Hong, X., S. Qiao, F. Li, W. Wang, R. Jiang, H. Wu, H. Chen, L. Liu, J. Peng, J. Wang, C. Jia, X. Liang, H. Dai, J. Jiang, T. Zhang, Q. Liao, M. Dai, L. Cong, X. Han, D. Guo, Z. Liang, D. Li, Z. Zheng, C. Ye, S. Li, Y. Zhao, K. Wu and W. Wu (2020). "Whole-genome sequencing reveals distinct genetic bases for insulinomas and non-functional pancreatic neuroendocrine tumours: leading to a new classification system." Gut **69**(5): 877-887.

Hu, J., W. Yu, Y. Dai, C. Liu, Y. Wang and Q. Wu (2022). "A Deep Neural Network for Gastric Cancer Prognosis Prediction Based on Biological Information Pathways." J Oncol **2022**: 2965166.

Huang, G., L. N. Howard, E. Alonsozana, D. Sill, D. Bose and J. Lai (2022). "Molecular Characteristics and Immunogenomic Profiling of Cholangioblastic Variant of Intrahepatic Cholangiocarcinoma in a 68-year-old Patient." Anticancer Res **42**(11): 5475-5478.

Iacovazzo, D., S. E. Flanagan, E. Walker, R. Quezado, F. A. de Sousa Barros, R. Caswell, M. B. Johnson, M. Wakeling, M. Brändle, M. Guo, M. N. Dang, P. Gabrovska, B. Niederle, E. Christ, S. Jenni, B. Sipos, M. Nieser, A. Frilling, K. Dhatariya, P. Chanson, W. W. de Herder, B. Konukiewitz, G. Klöppel, R. Stein, M. Korbonits and S. Ellard (2018). "MAFA missense mutation causes familial insulinomatosis and diabetes mellitus." Proc Natl Acad Sci U S A **115**(5): 1027-1032.

Ida, K., S. Kobayashi, T. Taki, R. Hanada, F. Bessho, S. Yamamori, T. Sugimoto, M. Ohki and Y. Hayashi (1995). "EWS-FLI-1 and EWS-ERG chimeric mRNAs in Ewing's sarcoma and primitive neuroectodermal tumor." Int J Cancer **63**(4): 500-504.

Iida, Y., Y. Nakanishi, T. Shimizu, M. Nomoto, Y. Nakagawa, R. Ito, N. Takahashi, S. Masuda and Y. Gon (2022). "Comprehensive genetic analysis of histological components of combined small cell carcinoma." Thorac Cancer **13**(16): 2362-2370.

Irshad, K., V. P. Jyotsna, S. Agarwal, K. Chosdol, S. Pal and R. K. Deepak (2017). "T372R Mutation Status in Yin Yang 1 Gene in Insulinoma Patients." Horm Metab Res **49**(6): 452-456.

Iyer, R., A. T. Phan and J. P. Boudreaux (2017). "Recent advances in the management of gastroenteropancreatic neuroendocrine tumors: insights from the 2017 ASCO Gastrointestinal Cancers Symposium." Clin Adv Hematol Oncol **15 Suppl 4**(4): 1-24.

Iyer, S., S. D. Modali and S. K. Agarwal (2017). "Long Noncoding RNA MEG3 Is an Epigenetic Determinant of Oncogenic Signaling in Functional Pancreatic Neuroendocrine Tumor Cells." Mol Cell Biol **37**(22).

Jeong, S. Y., Y. S. Park, J. Lee, J. Jang, Y. Jeon, Y. J. Jung, D. Choi, J. Hong and S. T. Kim (2023). "Tumor mutation burden in gastro-entero-pancreatic-neuroendocrine neoplasms." J Gastrointest Oncol **14**(4): 1707-1714.

Jiang, X. H., J. L. Lu, B. Cui, Y. J. Zhao, W. Q. Wang, J. M. Liu, W. Q. Fang, Y. N. Cao, Y. Ge, C. X. Zhang, H. Casse, X. Y. Li and G. Ning (2007). "MEN1 mutation analysis in Chinese patients with multiple endocrine neoplasia type 1." Endocr Relat Cancer **14**(4): 1073-1079.

Jyotsna, V. P., E. Malik, S. Birla and A. Sharma (2015). "Novel MEN 1 gene findings in rare sporadic insulinoma--a case control study." BMC Endocr Disord **15**: 44.

Karakose, E., H. Wang, W. Inabnet, R. V. Thakker, S. Libutti, G. Fernandez-Ranvier, H. Suh, M. Stevenson, Y. Kinoshita, M. Donovan, Y. Antipin, Y. Li, X. Liu, F. Jin, P. Wang, A. Uzilov, C. Argmann, E. E. Schadt, A. F. Stewart, D. K. Scott and L. Lambertini (2020). "Aberrant methylation underlies insulin gene expression in human insulinoma." Nature Communications **11**(1): 5210.

Katashima, R., M. Matsumoto, Y. Watanabe, M. Moritani and I. Yokota (2021). "Identification of Novel GCK and HNF4α Gene Variants in Japanese Pediatric Patients with Onset of Diabetes before 17 Years of Age." J Diabetes Res **2021**: 7216339.

Kawamura, J., Y. Shimada, I. Komoto, H. Okamoto, A. Itami, R. Doi, K. Fujimoto, S. Kosugi and M. Imamura (2005). "Multiple endocrine neoplasia type 1 gene mutations in sporadic gastrinomas in Japan." Oncol Rep **14**(1): 47-52.

Kawasaki, E., Y. Sera, K. Yamakawa, T. Abe, M. Ozaki, S. Uotani, N. Ohtsu, H. Takino, H. Yamasaki, Y. Yamaguchi, N. Matsuura and K. Eguchi (2000). "Identification and functional analysis of mutations in the hepatocyte nuclear factor-1alpha gene in anti-islet autoantibody-negative Japanese patients with type 1 diabetes." J Clin Endocrinol Metab **85**(1): 331-335.

Kawasaki, K., K. Toshimitsu, M. Matano, M. Fujita, M. Fujii, K. Togasaki, T. Ebisudani, M. Shimokawa, A. Takano, S. Takahashi, Y. Ohta, K. Nanki, R. Igarashi, K. Ishimaru, H. Ishida, Y. Sukawa, S. Sugimoto, Y. Saito, K. Maejima, S. Sasagawa, H. Lee, H. G. Kim, K. Ha, J. Hamamoto, K. Fukunaga, A. Maekawa, M. Tanabe, S. Ishihara, Y. Hamamoto, H. Yasuda, S. Sekine, A. Kudo, Y. Kitagawa, T. Kanai, H. Nakagawa and T. Sato (2020). "An Organoid Biobank of Neuroendocrine Neoplasms Enables Genotype-Phenotype Mapping." Cell **183**(5): 1420-1435.e1421.

Kelly, A. C., C. A. Bidwell, X. Chen, A. R. Macko, M. J. Anderson and S. W. Limesand (2018). "Chronic Adrenergic Signaling Causes Abnormal RNA Expression of Proliferative Genes in Fetal Sheep Islets." Endocrinology **159**(10): 3565-3578.

Kidd, M., I. M. Modlin, L. Bodei and I. Drozdov (2015). "Decoding the Molecular and Mutational Ambiguities of Gastroenteropancreatic Neuroendocrine Neoplasm Pathobiology." Cell Mol Gastroenterol Hepatol **1**(2): 131-153.

Kiesewetter, B. and M. Raderer (2020). "How I treat neuroendocrine tumours." ESMO Open **5**(4).

Kim, B.-Y., M.-H. Park, H.-M. Woo, H.-Y. Jo, J. H. Kim, H. J. Choi and S. K. Koo (2017). "Genetic analysis of parathyroid and pancreatic tumors in a patient with multiple endocrine neoplasia type 1 using whole-exome sequencing." BMC medical genetics **18**(1): 106-106.

Knappskog, S., T. Grob, A. Venizelos, U. Amstutz, G. O. Hjortland, I. M. Lothe, C. Kersten, E. Hofsli, A. Sundlöv, H. Elvebakken, H. Garresori, A. Couvelard, J. Svensson, H. Sorbye and A. Perren (2023). "Mutation Spectrum in Liquid Versus Solid Biopsies From Patients With Advanced Gastroenteropancreatic Neuroendocrine Carcinoma." JCO Precis Oncol **7**: e2200336.

Koch, A., A. Waha, J. C. Tonn, N. Sörensen, F. Berthold, M. Wolter, J. Reifenberger, W. Hartmann, W. Friedl, G. Reifenberger, O. D. Wiestler and T. Pietsch (2001). "Somatic mutations of WNT/wingless signaling pathway components in primitive neuroectodermal tumors." Int J Cancer **93**(3): 445-449.

Kong, G. and R. J. Hicks (2022). "PRRT for higher-grade neuroendocrine neoplasms: What is still acceptable?" Curr Opin Pharmacol **67**: 102293.

Kooblall, K. G., H. Boon, T. Cranston, M. Stevenson, A. T. Pagnamenta, A. Rogers, S. Grozinsky-Glasberg, T. Richardson, D. E. Flanagan, J. C. Taylor, K. E. Lines and R. V. Thakker (2021). "Multiple Endocrine Neoplasia Type 1 (MEN1) 5'UTR Deletion, in MEN1 Family, Decreases Menin Expression." J Bone Miner Res **36**(1): 100-109.

Korshunov, A., K. Okonechnikov, F. Schmitt-Hoffner, M. Ryzhova, F. Sahm, D. Stichel, D. Schrimpf, D. E. Reuss, P. Sievers, A. K. Suwala, E. Kumirova, O. Zheludkova, A. Golanov, D. T. W. Jones, S. M. Pfister, M. Kool and A. von Deimling (2021). "Molecular analysis of pediatric CNS-PNET revealed nosologic heterogeneity and potent diagnostic markers for CNS neuroblastoma with FOXR2-activation." Acta Neuropathol Commun **9**(1): 20.

Koyama, N., T. Nagase, M. Kure, T. Odaka, K. Kogure, Y. Takeda, T. Ishii, K. Narisawa, T. Fujita, M. Fujimori and Y. Katsura (2022). "Multiple Endocrine Neoplasia Type 1 with Functional Parathyroid Cysts." Intern Med **61**(8): 1183-1188.

Kubi, J. A., A. C. H. Chen, S. W. Fong, K. P. Lai, C. K. C. Wong, W. S. B. Yeung, K. F. Lee and Y. L. Lee (2019). "Effects of 2,3,7,8-tetrachlorodibenzo-p-dioxin (TCDD) on the differentiation of embryonic stem cells towards pancreatic lineage and pancreatic beta cell function." Environ Int **130**: 104885.

Kubota, Y., H. Kawakami, M. Natsuizaka, K. Kawakubo, K. Marukawa, T. Kudo, Y. Abe, K. Kubo, M. Kuwatani, Y. Hatanaka, T. Mitsuhashi, Y. Matsuno and N. Sakamoto (2015). "CTNNB1 mutational analysis of solid-pseudopapillary neoplasms of the pancreas using endoscopic ultrasound-guided fine-needle aspiration and next-generation deep sequencing." J Gastroenterol **50**(2): 203-210.

Kudo, N., J. Takano, S. Kudoh, N. Arima and T. Ito (2021). "INSM1 immunostaining in solid papillary carcinoma of the breast." Pathol Int **71**(1): 51-59.

Kövesdi, A., P. A. Kurucz, G. Nyírő, O. Darvasi, A. Patócs and H. Butz (2020). "Circulating miRNA Increases the Diagnostic Accuracy of Chromogranin A in Metastatic Pancreatic Neuroendocrine Tumors." Cancers (Basel) **12**(9).

Kövesdi, A., M. Tóth, H. Butz, N. Szücs, B. Sármán, P. Pusztai, J. Tőke, P. Reismann, M. Fáklya, G. Tóth, A. Somogyi, K. Borka, A. Erdei, E. V. Nagy, V. Deák, Z. Valkusz, P. Igaz, A. Patócs and V. K. Grolmusz (2019). "True MEN1 or phenocopy? Evidence for geno-phenotypic correlations in MEN1 syndrome." Endocrine **65**(2): 451-459.

Lagarde, A., G. Mougel, L. Coppin, M. Haissaguerre, L. Le Collen, A. Mohamed, M. Klein, M. F. Odou, A. Tabarin, H. Brixi, T. Cuny, B. Delemer, A. Barlier and P. Romanet (2022). "Systematic detection of mosaicism by using digital NGS reveals three new MEN1 mosaicisms." Endocr Connect **11**(11).

Lamberti, G., N. Prinzi, A. Bongiovanni, M. Torniai, E. Andrini, D. Biase, D. Malvi, M. Mosca, R. Berardi, T. Ibrahim, S. Pusceddu and D. Campana (2023). "Targeted Genomic Profiling and Chemotherapy Outcomes in Grade 3 Gastro-Entero-Pancreatic Neuroendocrine Tumors (G3 GEP-NET)." Diagnostics (Basel) **13**(9).

Lee, B., P. G. Bradford and S. G. Laychock (1998). "Characterization of inositol 1,4,5-trisphosphate receptor isoform mRNA expression and regulation in rat pancreatic islets, RINm5F cells and betaHC9 cells." J Mol Endocrinol **21**(1): 31-39.

Lee, M. S., S. Kim, J. H. Chung, M. K. Lee and K. W. Kim (1999). "Fas is expressed in murine pancreatic islet cells and an insulinoma cell line but does not mediate their apoptosis in vitro." Autoimmunity **29**(3): 189-199.

Lee, M. S. and B. H. O'Neil (2016). "Summary of emerging personalized medicine in neuroendocrine tumors: are we on track?" J Gastrointest Oncol **7**(5): 804-818.

Li, B. K., A. Vasiljevic, C. Dufour, F. Yao, B. L. B. Ho, M. Lu, E. I. Hwang, S. Gururangan, J. R. Hansford, M. Fouladi, S. Nobusawa, A. Laquerriere, M. B. Delisle, J. Fangusaro, F. Forest, H. Toledano, P. Solano-Paez, S. Leary, D. Birks, L. M. Hoffman, A. Szathmari, C. Faure-Conter, X. Fan, D. Catchpoole, L. Zhou, K. A. P. Schultz, K. Ichimura, G. Gauchotte, N. Jabado, C. Jones, D. Loussouarn, K. Mokhtari, A. Rousseau, D. S. Ziegler, S. Tanaka, S. L. Pomeroy, A. Gajjar, V. Ramaswamy, C. Hawkins, R. G. Grundy, D. A. Hill, E. Bouffet, A. Huang and A. Jouvet (2020). "Pineoblastoma segregates into molecular sub-groups with distinct clinico-pathologic features: a Rare Brain Tumor Consortium registry study." Acta Neuropathol **139**(2): 223-241.

Li, H., L. Zhao, R. Singh, J. N. Ham, D. O. Fadoju, L. J. H. Bean, Y. Zhang, Y. Xu, H. E. Xu and M. J. Gambello (2018). "The first pediatric case of glucagon receptor defect due to biallelic mutations in GCGR is identified by newborn screening of elevated arginine." Mol Genet Metab Rep **17**: 46-52.

Li, L., X. Zhang, X. Wang, S. W. Kim, J. M. Herndon, M. K. Becker-Hapak, B. M. Carreno, N. B. Myers, M. A. Sturmoski, M. D. McLellan, C. A. Miller, T. M. Johanns, B. R. Tan, G. P. Dunn, T. P. Fleming, T. H. Hansen, S. P. Goedegebuure and W. E. Gillanders (2021). "Optimized polyepitope neoantigen DNA vaccines elicit neoantigen-specific immune responses in preclinical models and in clinical translation." Genome Med **13**(1): 56.

Liang, M., J. Jiang, H. Dai, X. Hong, X. Han, L. Cong, A. Tong, F. Li, Y. Luo, W. Liu, L. Zhou, W. Di, W. Wu and Y. Zhao (2018). "Robotic enucleation for pediatric insulinoma with MEN1 syndrome: a case report and literature review." BMC Surg **18**(1): 44.

Libutti, S. K., P. L. Choyke, H. R. Alexander, G. Glenn, D. L. Bartlett, B. Zbar, I. Lubensky, S. A. McKee, E. R. Maher, W. M. Linehan and M. M. Walther (2000). "Clinical and genetic analysis of patients with pancreatic neuroendocrine tumors associated with von Hippel-Lindau disease." Surgery **128**(6): 1022-1027;discussion 1027-1028.

Lichtenauer, U. D., G. Di Dalmazi, E. P. Slater, T. Wieland, A. Kuebart, A. Schmittfull, T. Schwarzmayr, S. Diener, D. Wiese, W. E. Thasler, M. Reincke, T. Meitinger, M. Schott, M. Fassnacht, D. K. Bartsch, T. M. Strom and F. Beuschlein (2015). "Frequency and clinical correlates of somatic Ying Yang 1 mutations in sporadic insulinomas." J Clin Endocrinol Metab **100**(5): E776-782.

Lin, W., H. Watanabe, S. Peng, J. M. Francis, N. Kaplan, C. S. Pedamallu, A. Ramachandran, A. Agoston, A. J. Bass and M. Meyerson (2015). "Dynamic epigenetic regulation by menin during pancreatic islet tumor formation." Mol Cancer Res **13**(4): 689-698.

Lindberg, D., G. Akerström and G. Westin (2007). "Mutational analyses of WNT7A and HDAC11 as candidate tumour suppressor genes in sporadic malignant pancreatic endocrine tumours." Clin Endocrinol (Oxf) **66**(1): 110-114.

Lines, K. E., M. Stevenson, P. Filippakopoulos, S. Müller, H. E. Lockstone, B. Wright, S. Grozinsky-Glasberg, A. B. Grossman, S. Knapp, D. Buck, C. Bountra and R. V. Thakker (2017). "Epigenetic pathway inhibitors represent potential drugs for treating pancreatic and bronchial neuroendocrine tumors." Oncogenesis **6**(5): e332.

Ling, C., X. Hong, M. Xu, Y. Wang, X. Ma, Y. Cui, R. Jiang, D. Cao, H. Wu, A. Tong, Y. Zhao and W. Wu (2022). "Convergence between germline and somatic mutations in pancreatic neuroendocrine tumors." European Journal of Endocrinology **187**(1): 85-90.

Liu, A. P. Y., S. K. Dhanda, T. Lin, E. Sioson, A. Vasilyeva, B. Gudenas, R. G. Tatevossian, S. Jia, G. Neale, D. C. Bowers, T. Hassall, S. Partap, J. R. Crawford, M. Chintagumpala, E. Bouffet, G. McCowage, A. Broniscer, I. Qaddoumi, G. Armstrong, K. D. Wright, S. A. Upadhyaya, A. Vinitsky, C. L. Tinkle, J. Lucas, J. Chiang, D. J. Indelicato, R. Sanders, P. Klimo, Jr., F. A. Boop, T. E. Merchant, D. W. Ellison, P. A. Northcott, B. A. Orr, X. Zhou, A. Onar-Thomas, A. Gajjar and G. W. Robinson (2022). "Molecular classification and outcome of children with rare CNS embryonal tumors: results from St. Jude Children's Research Hospital including the multi-center SJYC07 and SJMB03 clinical trials." Acta Neuropathol **144**(4): 733-746.

Liu, B., L. H. Tang, Z. Liu, M. Mei, R. Yu, D. Dhall, X. W. Qiao, T. P. Zhang, Y. P. Zhao, T. H. Liu, Y. Xiao, J. Chen, H. D. Xiang, H. Y. Wu, C. M. Lu, B. Lv, Y. R. Zhou, Y. Zhang, D. Deng and Y. J. Chen (2014). "α-Internexin: a novel biomarker for pancreatic neuroendocrine tumor aggressiveness." J Clin Endocrinol Metab **99**(5): E786-795.

Liu, M., J. Zhu, H. Huang, Y. Chen and Z. Dong (2023). "Comparative analysis of nascent RNA sequencing methods and their applications in studies of co-transcriptional splicing dynamics." Plant Cell.

Lou, X., Z. Ye, X. Xu, M. Jiang, R. Lu, D. Jing, W. Zhang, H. Gao, F. Wang, Y. Zhang, X. Chen, Y. Qin, Q. Zhuo, X. Yu and S. Ji (2022). "Establishment and characterization of the third non-functional human pancreatic neuroendocrine tumor cell line." Hum Cell **35**(4): 1248-1261.

Lu, J., Q. Li, G. Donadel, A. L. Notkins and M. S. Lan (1998). "Profile and differential expression of protein tyrosine phosphatases in mouse pancreatic islet tumor cell lines." Pancreas **16**(4): 515-520.

Luley, K. B., S. B. Biedermann, A. Künstner, H. Busch, S. Franzenburg, J. Schrader, P. Grabowski, U. F. Wellner, T. Keck, G. Brabant, S. M. Schmid, H. Lehnert and H. Ungefroren (2020). "A Comprehensive Molecular Characterization of the Pancreatic Neuroendocrine Tumor Cell Lines BON-1 and QGP-1." Cancers (Basel) **12**(3).

Ma, C. H., H. B. Guo, X. Y. Pan and W. X. Zhang (2020). "Comprehensive treatment of rare multiple endocrine neoplasia type 1: A case report." World J Clin Cases **8**(12): 2647-2654.

MacDonald, M. J., R. D. Husain, S. Hoffmann-Benning and T. R. Baker (2004). "Immunochemical identification of coenzyme Q0-dihydrolipoamide adducts in the E2 components of the alpha-ketoglutarate and pyruvate dehydrogenase complexes partially explains the cellular toxicity of coenzyme Q0." J Biol Chem **279**(26): 27278-27285.

Mahjoub, A. R. and E. M. O'Reilly (2013). "Emerging therapies for pancreas neuroendocrine cancers." Chin Clin Oncol **2**(3): 23.

Mandal, P., D. De, D. U. Im, S. H. Um and K. K. Kim (2020). "Exosome-Mediated Differentiation of Mouse Embryonic Fibroblasts and Exocrine Cells into β-Like Cells and the Identification of Key miRNAs for Differentiation." Biomedicines **8**(11).

Manialawy, Y., S. R. Khan, A. Bhattacharjee and M. B. Wheeler (2020). "The magnesium transporter NIPAL1 is a pancreatic islet-expressed protein that conditionally impacts insulin secretion." J Biol Chem **295**(29): 9879-9892.

Martin, D. R., E. LaBauve, J. M. Pomo, V. K. Chiu, J. A. Hanson and R. R. Gullapalli (2018). "Site-Specific Genomic Alterations in a Well-Differentiated Pancreatic Neuroendocrine Tumor With High-Grade Progression." Pancreas **47**(4): 502-510.

Mathew, J. G., A. S. Bowman, J. Saab, K. J. Busam, K. Nehal and M. Pulitzer (2022). "Next-generation sequencing analysis suggests varied multistep mutational pathogenesis for endocrine mucin-producing sweat gland carcinoma with comments on INSM1 and MUC2 suggesting a conjunctival origin." J Am Acad Dermatol **86**(5): 1072-1079.

Matsutani, A., Y. Takeuchi, H. Ishihara, S. Kuwano and Y. Oka (1996). "Molecular cloning of human mitochondrial glycerophosphate dehydrogenase gene: genomic structure, chromosomal localization, and existence of a pseudogene." Biochem Biophys Res Commun **223**(3): 481-486.

Maxwell, J. E., S. K. Sherman and J. R. Howe (2016). "Translational Diagnostics and Therapeutics in Pancreatic Neuroendocrine Tumors." Clin Cancer Res **22**(20): 5022-5029.

Mellai, M., A. Piazzi, V. Caldera, O. Monzeglio, P. Cassoni, G. Valente and D. Schiffer (2011). "IDH1 and IDH2 mutations, immunohistochemistry and associations in a series of brain tumors." J Neurooncol **105**(2): 345-357.

Men, L., J. Sun, G. Luo and D. Ren (2019). "Acute Deletion of METTL14 in β-Cells of Adult Mice Results in Glucose Intolerance." Endocrinology **160**(10): 2388-2394.

Miller, H. C., M. Kidd, I. M. Modlin, P. Cohen, R. Dina, P. Drymousis, P. Vlavianos, G. Klöppel and A. Frilling (2015). "Glucagon receptor gene mutations with hyperglucagonemia but without the glucagonoma syndrome." World J Gastrointest Surg **7**(4): 60-66.

Miller, S., J. H. Ward, H. A. Rogers, J. Lowe and R. G. Grundy (2013). "Loss of INI1 protein expression defines a subgroup of aggressive central nervous system primitive neuroectodermal tumors." Brain Pathol **23**(1): 19-27.

Miranda, I. S. M., L. P. Valadares, G. B. Barra, P. G. Mesquita, L. B. de Santana, L. F. de Castro, T. H. S. Rita and L. A. Naves (2023). "Clinical and molecular features of four Brazilian families with multiple endocrine neoplasia type 1." Front Endocrinol (Lausanne) **14**: 1117873.

Mohammed Almehmadi, D., A. Saleh Dairi, A. Ali Hassan, A. Dannoun, H. Saleh Banni, M. M. E, A. R. Youssef and M. T. M (2021). "Identification of PKHD1 mutations in Brain, Breast and Rectal tumors by Next Generation DNA Sequencing." Gulf J Oncolog **1**(35): 42-53.

Moon, S., J. W. Lee, D. Shin, K. Y. Shin, J. Kim, I. Y. Choi, J. Kim and H. Kim (2015). "A Genome-wide Scan for Selective Sweeps in Racing Horses." Asian-Australas J Anim Sci **28**(11): 1525-1531.

Moore, F., D. A. Cunha, H. Mulder and D. L. Eizirik (2012). "Use of RNA interference to investigate cytokine signal transduction in pancreatic beta cells." Methods Mol Biol **820**: 179-194.

Muntasell, A., M. Carrascal, L. Serradell, P. Veelen Pv, F. Verreck, F. Koning, G. Raposo, J. Abián and D. Jaraquemada (2002). "HLA-DR4 molecules in neuroendocrine epithelial cells associate to a heterogeneous repertoire of cytoplasmic and surface self peptides." J Immunol **169**(9): 5052-5060.

Murat Cde, B., P. W. da Rosa, M. A. Fortes, L. Corrêa, M. C. Machado, E. M. Novak, S. A. Siqueira, M. A. Pereira, M. L. Corrêa-Giannella, D. Giannella-Neto and R. R. Giorgi (2015). "Differential expression of genes encoding proteins of the HGF/MET system in insulinomas." Diabetol Metab Syndr **7**: 84.

Mühlisch, J., A. Schwering, M. Grotzer, G. H. Vince, W. Roggendorf, C. Hagemann, N. Sörensen, C. H. Rickert, N. Osada, H. Jürgens and M. C. Frühwald (2006). "Epigenetic repression of RASSF1A but not CASP8 in supratentorial PNET (sPNET) and atypical teratoid/rhabdoid tumors (AT/RT) of childhood." Oncogene **25**(7): 1111-1117.

Mönig, H., I. U. Ali, E. H. Oldfield and H. M. Schulte (1993). "Structure of the POMC promoter region in pituitary and extrapituitary ACTH producing tumors." Exp Clin Endocrinol **101**(1): 36-38.

Niehusmann, P., E. Stensvold, H. Leske, T. Pietsch, T. Goschzik, G. H. Gielen, B. Due-Tønnessen, R. Frič, Y. Nilssen and P. Brandal (2022). "Molecular pathological insights reveal a high number of unfavorable risk patients among children treated for medulloblastoma and CNS-PNET in Oslo 2005-2017." Pediatr Blood Cancer **69**(9): e29736.

Nielsen, E., B. Welinder and O. D. Madsen (1990). "Protein HMG-17 is hyper-expressed in rat glucagonoma. Single-step isolation and sequencing." Eur J Biochem **192**(1): 81-86.

Niemeijer, N. D., T. G. Papathomas, E. Korpershoek, R. R. de Krijger, L. Oudijk, H. Morreau, J. P. Bayley, F. J. Hes, J. C. Jansen, W. N. Dinjens and E. P. Corssmit (2015). "Succinate Dehydrogenase (SDH)-Deficient Pancreatic Neuroendocrine Tumor Expands the SDH-Related Tumor Spectrum." J Clin Endocrinol Metab **100**(10): E1386-1393.

Nishi, M., T. Sanke, S. Ohagi, K. Ekawa, H. Wakasaki, K. Nanjo, G. I. Bell and D. F. Steiner (1992). "Molecular biology of islet amyloid polypeptide." Diabetes Res Clin Pract **15**(1): 37-44.

Noda, K., T. Kuroki, M. Yamashita, T. Hirayama, K. Natsuda, S. Kobayashi, T. Tokunaga, K. Yamanouchi, H. Takeshita, S. Miura and S. Maeda (2020). "Nondegenerated cystic neuroendocrine tumor of the pancreas: a case report." Surg Case Rep **6**(1): 154.

Oberg, K. (2018). "Management of functional neuroendocrine tumors of the pancreas." Gland Surg **7**(1): 20-27.

Okada, Y., S. Kamata, T. Akashi, M. Kurata, T. Nakamura and K. Kihara (2011). "Primitive neuroectodermal tumor/Ewing's sarcoma of the urinary bladder: a case report and its molecular diagnosis." Int J Clin Oncol **16**(4): 435-438.

Okamoto, H., A. Tamada, N. Hai, M. Doi, I. Uchimura, Y. Hirata and S. Kosugi (2002). "A novel six-nucleotide insertion in exon 4 of the MEN1 gene, 878insCTGCAG, in three patients with familial insulinoma and primary hyperparathyroidism." Jpn J Clin Oncol **32**(9): 368-370.

Orr-Asman, M. A., Z. Chu, M. Jiang, M. Worley, K. LaSance, S. E. Koch, V. S. Carreira, H. M. Dahche, D. R. Plas, K. Komurov, X. Qi, C. A. Mercer, L. B. Anthony, J. Rubinstein and H. E. Thomas (2017). "mTOR Kinase Inhibition Effectively Decreases Progression of a Subset of Neuroendocrine Tumors that Progress on Rapalog Therapy and Delays Cardiac Impairment." Mol Cancer Ther **16**(11): 2432-2441.

Panarelli, N., K. Tyryshkin, J. J. M. Wong, A. Majewski, X. Yang, T. Scognamiglio, M. K. Kim, K. Bogardus, T. Tuschl, Y. T. Chen and N. Renwick (2019). "Evaluating gastroenteropancreatic neuroendocrine tumors through microRNA sequencing." Endocr Relat Cancer **26**(1): 47-57.

Pardi, E., S. Borsari, F. Saponaro, F. Bogazzi, C. Urbani, S. Mariotti, F. Pigliaru, C. Satta, F. Pani, G. Materazzi, P. Miccoli, L. Grantaliano, C. Marcocci and F. Cetani (2017). "Mutational and large deletion study of genes implicated in hereditary forms of primary hyperparathyroidism and correlation with clinical features." PLoS One **12**(10): e0186485.

Pardi, E., S. Mariotti, N. S. Pellegata, K. Benfini, S. Borsari, F. Saponaro, L. Torregrossa, A. Cappai, C. Satta, M. Mastinu, C. Marcocci and F. Cetani (2015). "Functional characterization of a CDKN1B mutation in a Sardinian kindred with multiple endocrine neoplasia type 4 (MEN4)." Endocr Connect **4**(1): 1-8.

Parija, T., S. Shirley, S. Uma, K. R. Rajalekshmy, S. Ayyappan and T. Rajkumar (2005). "Type 1 (11;22)(q24:q12) translocation is common in Ewing's sarcoma/peripheral neuroectodermal tumour in south Indian patients." J Biosci **30**(3): 371-376.

Park, C., S. Y. Ha, S. T. Kim, H. C. Kim, J. S. Heo, Y. S. Park, G. Lauwers, J. Lee and K. M. Kim (2016). "Identification of the BRAF V600E mutation in gastroenteropancreatic neuroendocrine tumors." Oncotarget **7**(4): 4024-4035.

Patel, D., D. Chan, G. Cehic, N. Pavlakis and T. J. Price (2016). "Systemic therapies for advanced gastroenteropancreatic neuroendocrine tumors." Expert Rev Endocrinol Metab **11**(4): 311-327.

Pea, A., R. H. Hruban and L. D. Wood (2015). "Genetics of pancreatic neuroendocrine tumors: implications for the clinic." Expert Rev Gastroenterol Hepatol **9**(11): 1407-1419.

Pelosi, G., F. Bianchi, E. Dama, J. Metovic, M. Barella, A. Sonzogni, A. Albini, M. Papotti, Y. Gong and N. Vijayvergia (2021). "A Subset of Large Cell Neuroendocrine Carcinomas in the Gastroenteropancreatic Tract May Evolve from Pre-existing Well-Differentiated Neuroendocrine Tumors." Endocr Pathol **32**(3): 396-407.

Peng, W., L. Cao, L. Chen, G. Lin, B. Zhu, X. Hu, Y. Lin, S. Zhang, M. Jiang, J. Wang, J. Li, C. Li, L. Shao, H. Du, T. Hou, Z. Chen, J. Xiang, X. Pu, J. Li, F. Xu, H. Loong and L. Wu (2022). "Comprehensive Characterization of the Genomic Landscape in Chinese Pulmonary Neuroendocrine Tumors Reveals Prognostic and Therapeutic Markers (CSWOG-1901)." Oncologist **27**(2): e116-e125.

Perren, A., P. Wiesli, S. Schmid, M. Montani, A. Schmitt, C. Schmid, H. Moch and P. Komminoth (2006). "Pancreatic endocrine tumors are a rare manifestation of the neurofibromatosis type 1 phenotype: molecular analysis of a malignant insulinoma in a NF-1 patient." Am J Surg Pathol **30**(8): 1047-1051.

Perrier, N. D. (2018). "From Initial Description by Wermer to Present-Day MEN1: What have We Learned?" World J Surg **42**(4): 1031-1035.

Philipson, L. H., A. Kusnetsov, T. Larson, Y. Zeng and G. Westermark (1993). "Human, rodent, and canine pancreatic beta-cells express a sodium channel alpha 1-subunit related to a fetal brain isoform." Diabetes **42**(9): 1372-1377.

Pivovarcikova, K., A. Agaimy, P. Martinek, R. Alaghehbandan, D. Perez-Montiel, I. Alvarado-Cabrero, J. Rogala, N. Kuroda, B. Rychly, S. Gasparov, K. Michalova, M. Michal, M. Hora, T. Pitra, I. Tuckova, S. Laciok, J. Mareckova and O. Hes (2019). "Primary renal well-differentiated neuroendocrine tumour (carcinoid): next-generation sequencing study of 11 cases." Histopathology **75**(1): 104-117.

Portwine, C., S. Chilton-MacNeill, C. Brown, E. Sexsmith, J. McLaughlin and D. Malkin (2001). "Absence of germline and somatic p53 alterations in children with sporadic brain tumors." J Neurooncol **52**(3): 227-235.

Pozas, J., T. Alonso-Gordoa, M. S. Román, M. Santoni, C. Thirlwell, E. Grande and J. Molina-Cerrillo (2022). "Novel therapeutic approaches in GEP-NETs based on genetic and epigenetic alterations." Biochim Biophys Acta Rev Cancer **1877**(5): 188804.

Prisciandaro, M., M. Antista, A. Raimondi, F. Corti, F. Morano, G. Centonze, G. Sabella, A. Mangogna, G. Randon, F. Pagani, N. Prinzi, M. Niger, S. Corallo, E. Castiglioni di Caronno, M. Massafra, M. D. Bartolomeo, F. de Braud, M. Milione and S. Pusceddu (2022). "Biomarker Landscape in Neuroendocrine Tumors With High-Grade Features: Current Knowledge and Future Perspective." Front Oncol **12**: 780716.

Puccini, A., K. Poorman, M. E. Salem, D. Soldato, A. Seeber, R. M. Goldberg, A. F. Shields, J. Xiu, F. Battaglin, M. D. Berger, R. Tokunaga, M. Naseem, A. Barzi, S. Iqbal, W. Zhang, S. Soni, J. J. Hwang, P. A. Philip, S. Sciallero, W. M. Korn, J. L. Marshall and H. J. Lenz (2020). "Comprehensive Genomic Profiling of Gastroenteropancreatic Neuroendocrine Neoplasms (GEP-NENs)." Clin Cancer Res **26**(22): 5943-5951.

Quevedo, R., A. Spreafico, J. Bruce, A. Danesh, S. El Ghamrasni, A. Giesler, Y. Hanna, C. Have, T. Li, S. Y. C. Yang, T. Zhang, S. L. Asa, B. Haibe-Kains, M. Krzyzanowska, A. C. Smith, S. Singh, L. L. Siu and T. J. Pugh (2020). "Centromeric cohesion failure invokes a conserved choreography of chromosomal mis-segregations in pancreatic neuroendocrine tumor." Genome Med **12**(1): 38.

Rabinowits, G., J. Barletta, L. M. Sholl, E. Reche, J. Lorch and L. Goguen (2017). "Successful Management of a Patient with Malignant Thyroid Teratoma." Thyroid **27**(1): 125-128.

Radig, K., R. Schneider-Stock, I. Röse, U. Mittler, Y. Oda and A. Roessner (1998). "p53 and ras mutations in Ewing's sarcoma." Pathol Res Pract **194**(3): 157-162.

Rafaeloff, R., G. L. Pittenger, S. W. Barlow, X. F. Qin, B. Yan, L. Rosenberg, W. P. Duguid and A. I. Vinik (1997). "Cloning and sequencing of the pancreatic islet neogenesis associated protein (INGAP) gene and its expression in islet neogenesis in hamsters." J Clin Invest **99**(9): 2100-2109.

Raghavan, R., S. Shah, A. A. Kondkar, A. J. Dherai, D. Desai, P. Chauhan, M. Lala and T. F. Ashavaid (2007). "MEN1 935-1G>C splicing mutation in an Indian patient with multiple endocrine neoplasia type 1." Mol Diagn Ther **11**(2): 129-131.

Raj, N., K. Coffman, T. Le, R. K. G. Do, J. Rafailov, Y. Choi, J. F. Chou, M. Capanu, M. Dunphy, J. J. Fox, R. K. Grewal, R. P. Reddy, C. Riedl, H. Schoder, L. Bodei and D. Reidy-Lagunes (2022). "Treatment Response and Clinical Outcomes of Well-Differentiated High-Grade Neuroendocrine Tumors to Lutetium-177-DOTATATE." Neuroendocrinology **112**(12): 1177-1186.

Ramage, J., B. G. Naraev and T. R. Halfdanarson (2018). "Peptide receptor radionuclide therapy for patients with advanced pancreatic neuroendocrine tumors." Semin Oncol **45**(4): 236-248.

Raoul, J. L., M. F. Heymann, F. Dumont, A. Morel, H. Senellart and F. Bertucci (2021). "Case Report: Grade 2 Metastatic Pancreatic Neuroendocrine Tumor With Progression of One Metastasis After Pregnancy to Grade 3 Large-Cell Neuroendocrine Carcinoma: One Case Cured by Resection With Genomic Characterization of the Two Components." Front Oncol **11**: 646992.

Raymond, L. M., T. Korzun, A. Kardosh, K. J. Kolbeck, R. Pommier and E. S. Mittra (2021). "The State of Peptide Receptor Radionuclide Therapy and Its Sequencing among Current Therapeutic Options for Gastroenteropancreatic Neuroendocrine Tumors." Neuroendocrinology **111**(11): 1086-1098.

Regnell, S. E. and Å. Lernmark (2017). "Early prediction of autoimmune (type 1) diabetes." Diabetologia **60**(8): 1370-1381.

Rekhi, B., R. Basak, S. B. Desai and N. A. Jambhekar (2010). "A t (11; 22) (p13; q12) EWS-WT 1 positive desmoplastic small round cell tumor of the maxilla: an unusual case indicating the role of molecular diagnosis in round cell sarcomas." J Postgrad Med **56**(3): 201-205.

Remon, J., L. Lacroix, C. Jovelet, C. Caramella, K. Howarth, V. Plagnol, N. Rosenfeld, C. Morris, L. Mezquita, C. Pannet, M. Ngocamus, C. Le Pechoux, J. Adam, A. M. Grecea, D. Planchard, G. Vassal, J. C. Benitez, A. Gazzah, E. Green, J. C. Soria and B. Besse (2019). "Real-World Utility of an Amplicon-Based Next-Generation Sequencing Liquid Biopsy for Broad Molecular Profiling in Patients With Advanced Non-Small-Cell Lung Cancer." JCO Precis Oncol **3**.

Rico, K., S. Duan, R. L. Pandey, Y. Chen, J. T. Chakrabarti, J. Starr, Y. Zavros, T. Else, B. W. Katona, D. C. Metz and J. L. Merchant (2021). "Genome analysis identifies differences in the transcriptional targets of duodenal versus pancreatic neuroendocrine tumours." BMJ Open Gastroenterol **8**(1).

Roy, S., W. A. LaFramboise, T. C. Liu, D. Cao, A. Luvison, C. Miller, M. A. Lyons, R. J. O'Sullivan, A. H. Zureikat, M. E. Hogg, A. Tsung, K. K. Lee, N. Bahary, R. E. Brand, J. S. Chennat, K. E. Fasanella, K. McGrath, M. N. Nikiforova, G. I. Papachristou, A. Slivka, H. J. Zeh and A. D. Singhi (2018). "Loss of Chromatin-Remodeling Proteins and/or CDKN2A Associates With Metastasis of Pancreatic Neuroendocrine Tumors and Reduced Patient Survival Times." Gastroenterology **154**(8): 2060-2063.e2068.

Saeed, R., A. K. Mohammed, S. E. Saleh, K. M. Aboshanab, M. M. Aboulwafa and J. Taneera (2023). "Expression Silencing of Mitogen-Activated Protein Kinase 8 Interacting Protein-1 Conferred Its Role in Pancreatic β-Cell Physiology and Insulin Secretion." Metabolites **13**(2).

Saif, M. W., R. Parikh, D. Ray, J. A. Kaye, S. K. Kurosky, K. Thomas, R. A. Ramirez, T. R. Halfdanarson, T. J. R. Beveridge, B. Mirakhur, S. P. Nagar and H. P. Soares (2019). "Medical record review of transition to lanreotide following octreotide for neuroendocrine tumors." J Gastrointest Oncol **10**(4): 674-687.

Sakurai, M., T. Wakabayashi, Y. Kondo, E. Ikeda, K. Watanabe, A. Takei, H. Okazaki, K. Okada, K. Ebihara, N. Kakiuchi, S. Ogawa, N. Fukushima and S. Ishibashi (2023). "A case of vasoactive intestinal peptide-secreting tumor (VIPoma) arising from MEN1 inactivation which recurred 15 years after the initial resection." Endocr J.

Salinno, C., M. Büttner, P. Cota, S. Tritschler, M. Tarquis-Medina, A. Bastidas-Ponce, K. Scheibner, I. Burtscher, A. Böttcher, F. J. Theis, M. Bakhti and H. Lickert (2021). "CD81 marks immature and dedifferentiated pancreatic β-cells." Mol Metab **49**: 101188.

Saoud, C., A. A. Wu, E. K. Fishman, R. H. Hruban and S. Z. Ali (2023). "Pancreatoblastoma in an elderly woman: A case report simulating a pancreatic neuroendocrine tumour with emphasis on cytomorphological features." Cytopathology **34**(3): 254-258.

Sasaki, S., R. Tomomasa, S. Nobusawa, J. Hirato, T. Uchiyama, E. Boku, T. Miyasaka, T. Hirose and C. Ohbayashi (2019). "Anaplastic pleomorphic xanthoastrocytoma associated with an H3G34 mutation: a case report with review of literature." Brain Tumor Pathol **36**(4): 169-173.

Scarpa, A. (2019). "The landscape of molecular alterations in pancreatic and small intestinal neuroendocrine tumours." Ann Endocrinol (Paris) **80**(3): 153-158.

Scharfmann, R., A. Tazi, M. Polak, C. Kanaka and P. Czernichow (1993). "Expression of functional nerve growth factor receptors in pancreatic beta-cell lines and fetal rat islets in primary culture." Diabetes **42**(12): 1829-1836.

Schmitz, R. L., J. Weissbach, J. Kleilein, J. Bell, S. Hüttelmaier, F. Viol, T. Clauditz, P. Grabowski, H. Laumen, J. Rosendahl, P. Michl, J. Schrader and S. Krug (2021). "Targeting HDACs in Pancreatic Neuroendocrine Tumor Models." Cells **10**(6).

Schuppe, H. C., N. J. Neumann, G. Schock-Skasa, W. Höppner and J. Feldkamp (1999). "Secondary infertility as early symptom in a man with multiple endocrine neoplasia-type 1." Hum Reprod **14**(1): 252-254.

Schaaf, L., J. Pickel, K. Zinner, U. Hering, M. Höfler, P. E. Goretzki, F. Spelsberg, F. Raue, A. von zur Mühlen, H. Gerl, J. Hensen, D. K. Bartsch, M. Rothmund, U. Schneyer, H. Dralle, M. Engelbach, W. Karges, G. K. Stalla and W. Höppner (2007). "Developing effective screening strategies in multiple endocrine neoplasia type 1 (MEN 1) on the basis of clinical and sequencing data of German patients with MEN 1." Exp Clin Endocrinol Diabetes **115**(8): 509-517.

Scoville, S. D., J. M. Cloyd and T. M. Pawlik (2020). "New and emerging systemic therapy options for well-differentiated gastroenteropancreatic neuroendocrine tumors." Expert Opin Pharmacother **21**(2): 183-191.

Seabrook, A., A. Wijewardene, S. De Sousa, T. Wong, N. Sheriff, A. J. Gill, R. Iyer, M. Field, C. Luxford, R. Clifton-Bligh, A. McCormack and K. Tucker (2022). "MEN4, the MEN1 Mimicker: A Case Series of three Phenotypically Heterogenous Patients With Unique CDKN1B Mutations." J Clin Endocrinol Metab **107**(8): 2339-2349.

Shah, S. R., R. Raghavan, D. C. Desai, P. H. Chauhan, M. Lala, A. J. Dherai and T. F. Ashavaid (2008). "An Indian family of multiple endocrine neoplasia type 1 (MEN1): molecular diagnosis, treatment and follow up." Indian J Gastroenterol **27**(6): 242-244.

Shan, L., Y. Nakamura, M. Nakamura, T. Yokoi, M. Tsujimoto, R. Arima, T. Kameya and K. Kakudo (1998). "Somatic mutations of multiple endocrine neoplasia type 1 gene in the sporadic endocrine tumors." Lab Invest **78**(4): 471-475.

Shaw, C., K. Cormican, L. Thim, A. G. Maule, J. M. Sloan and K. D. Buchanan (1993). "Neuropeptide Y and neuropeptide Y 3-36: isolation from human pancreatic endocrine tumours." Regul Pept **45**(3): 387-394.

Shell, J., D. Patel, A. Powers, M. Quezado, K. Killian, P. Meltzer, J. Zhu, A. Gaitanidis, F. Karzai, V. Neychev, P. Green and E. Kebebew (2017). "Somatic VHL Mutation in a Patient With MEN1-Associated Metastatic Pancreatic Neuroendocrine Tumor Responding to Sunitinib Treatment: A Case Report." J Endocr Soc **1**(9): 1124-1134.

Shiratsuchi, H., T. Saito, A. Sakamoto, E. Itakura, S. Tamiya, Y. Oshiro, Y. Oda, S. Toh, S. Komiyama and M. Tsuneyoshi (2002). "Mutation analysis of human cytokeratin 8 gene in malignant rhabdoid tumor: a possible association with intracytoplasmic inclusion body formation." Mod Pathol **15**(2): 146-153.

Shulkes, A. (1994). "Somatostatin: physiology and clinical applications." Baillieres Clin Endocrinol Metab **8**(1): 215-236.

Simbolo, M., G. Centonze, L. Giudice, F. Grillo, P. Maisonneuve, A. Gkountakos, C. Ciaparrone, L. Cattaneo, G. Sabella, R. Giugno, P. Bossi, P. Spaggiari, A. Del Gobbo, S. Ferrero, L. Mastracci, A. Fabbri, M. Filugelli, G. Garzone, N. Prinzi, S. Pusceddu, A. Testi, V. Monti, L. Rolli, A. Mangogna, L. Bercich, M. R. Benvenuti, E. Bria, S. Pilotto, A. Berruti, U. Pastorino, C. Capella, M. Infante, M. Milella, A. Scarpa and M. Milione (2022). "Combined Large Cell Neuroendocrine Carcinomas of the Lung: Integrative Molecular Analysis Identifies Subtypes with Potential Therapeutic Implications." Cancers (Basel) **14**(19).

Singh, S., T. A. Hope, E. B. Bergsland, L. Bodei, D. L. Bushnell, J. A. Chan, B. R. Chasen, A. Chauhan, S. Das, A. Dasari, J. Del Rivero, G. El-Haddad, K. A. Goodman, D. M. Halperin, M. A. Lewis, O. W. Lindwasser, S. Myrehaug, N. P. Raj, D. L. Reidy-Lagunes, H. P. Soares, J. R. Strosberg, E. C. Kohn and P. L. Kunz (2023). "Consensus report of the 2021 National Cancer Institute neuroendocrine tumor clinical trials planning meeting." J Natl Cancer Inst **115**(9): 1001-1010.

Singhi, A. D., T. C. Liu, J. L. Roncaioli, D. Cao, H. J. Zeh, A. H. Zureikat, A. Tsung, J. W. Marsh, K. K. Lee, M. E. Hogg, N. Bahary, R. E. Brand, K. M. McGrath, A. Slivka, K. L. Cressman, K. Fuhrer and R. J. O'Sullivan (2017). "Alternative Lengthening of Telomeres and Loss of DAXX/ATRX Expression Predicts Metastatic Disease and Poor Survival in Patients with Pancreatic Neuroendocrine Tumors." Clin Cancer Res **23**(2): 600-609.

Skalniak, A., M. Trofimiuk-Müldner, A. Jabrocka-Hybel, J. Totoń-Żurańska, P. Wołkow and A. Hubalewska-Dydejczyk (2023). "Whole-exome sequencing as a tool for searching for genetic background modifiers in MEN1 patients with neuroendocrine pancreatic tumours, including insulinomas." Endokrynol Pol **74**(1): 31-46.

Song, Y. L., J. Xu, D. C. Zhao, T. P. Zhang, K. Z. Jin, L. M. Zhu, S. Yu and Y. J. Chen (2021). "Mutation and Expression of Gene YY1 in Pancreatic Neuroendocrine Tumors and Its Clinical Significance." Endocr Pract **27**(9): 874-880.

Song, Y. L., R. Yu, X. W. Qiao, C. M. Bai, C. M. Lu, Y. Xiao, D. R. Zhong, J. Chen, Y. P. Zhao, T. P. Zhang, T. T. Song, H. L. Gao, Y. H. Wan, L. Shen, J. Chen, B. Lv, J. J. Hao, Y. Zhang, L. Tang and Y. J. Chen (2017). "Prognostic relevance of UCH-L1 and α-internexin in pancreatic neuroendocrine tumors." Sci Rep **7**(1): 2205.

Stevenson, M., K. E. Lines and R. V. Thakker (2018). "Molecular Genetic Studies of Pancreatic Neuroendocrine Tumors: New Therapeutic Approaches." Endocrinol Metab Clin North Am **47**(3): 525-548.

Strosberg, J., J. Goldman, F. Costa and M. Pavel (2015). "The Role of Chemotherapy in Well-Differentiated Gastroenteropancreatic Neuroendocrine Tumors." Front Horm Res **44**: 239-247.

Suemitsu, Y., Y. Ono, Y. Mizukami, J. Ye, K. Yamakawa, T. Takamoto, Y. Nakano-Narusawa, Y. Mukai, M. Takamatsu, A. Nakazawa, M. Mino-Kenudson, T. Kumasaka and Y. Matsuda (2021). "A Case of Adult Pancreatoblastoma With Novel APC Mutation and Genetic Heterogeneity." Front Oncol **11**: 725290.

Sumegi, J., J. Nishio, M. Nelson, R. W. Frayer, D. Perry and J. A. Bridge (2011). "A novel t(4;22)(q31;q12) produces an EWSR1-SMARCA5 fusion in extraskeletal Ewing sarcoma/primitive neuroectodermal tumor." Mod Pathol **24**(3): 333-342.

Sun, C., J. S. Estrella, E. M. Whitley, G. P. Chau, G. Lozano and A. R. Wasylishen (2022). "Context matters - Daxx and Atrx are not robust tumor suppressors in the murine endocrine pancreas." Dis Model Mech **15**(8).

Sun, T. Y., L. Zhao, P. Van Hummelen, B. Martin, K. Hornbacker, H. Lee, L. C. Xia, S. K. Padda, H. P. Ji and P. Kunz (2022). "Exploratory genomic analysis of high-grade neuroendocrine neoplasms across diverse primary sites." Endocr Relat Cancer **29**(12): 665-679.

Suwala, A. K., D. Stichel, D. Schrimpf, S. L. N. Maas, M. Sill, H. Dohmen, R. Banan, A. Reinhardt, P. Sievers, F. Hinz, M. Blattner-Johnson, C. Hartmann, L. Schweizer, H. B. Boldt, B. W. Kristensen, J. Schittenhelm, M. D. Wood, G. Chotard, R. Bjergvig, A. Das, U. Tabori, M. Hasselblatt, A. Korshunov, Z. Abdullaev, M. Quezado, K. Aldape, P. N. Harter, M. Snuderl, J. Hench, S. Frank, T. Acker, S. Brandner, F. Winkler, P. Wesseling, S. M. Pfister, D. E. Reuss, W. Wick, A. von Deimling, D. T. W. Jones and F. Sahm (2021). "Glioblastomas with primitive neuronal component harbor a distinct methylation and copy-number profile with inactivation of TP53, PTEN, and RB1." Acta Neuropathol **142**(1): 179-189.

Szkukalek, J., R. Dóczi, A. Dirner, Á. Boldizsár, Á. Varga, J. Déri, D. Lakatos, D. Tihanyi, B. Vodicska, R. Schwáb, G. Pajkos, E. Várkondi, I. Vályi-Nagy, D. Valtinyi, Z. Nagy and I. Peták (2021). "Personalized First-Line Treatment of Metastatic Pancreatic Neuroendocrine Carcinoma Facilitated by Liquid Biopsy and Computational Decision Support." Diagnostics (Basel) **11**(10).

Taboada, R., L. Claro, T. Felismino, V. H. de Jesus, M. Barros and R. P. Riechelmann (2022). "Clinicopathological and molecular profile of grade 3 gastroenteropancreatic neuroendocrine neoplasms." J Neuroendocrinol **34**(4): e13099.

Tang, L. H., T. Contractor, R. Clausen, D. S. Klimstra, Y. C. Du, P. J. Allen, M. F. Brennan, A. J. Levine and C. R. Harris (2012). "Attenuation of the retinoblastoma pathway in pancreatic neuroendocrine tumors due to increased cdk4/cdk6." Clin Cancer Res **18**(17): 4612-4620.

Tateno, M., Y. Fukunishi, S. Komatsu, Y. Okazaki, J. Kawai, K. Shibata, M. Itoh, M. Muramatsu, W. A. Held and Y. Hayashizaki (2001). "Identification of a novel member of the snail/Gfi-1 repressor family, mlt 1, which is methylated and silenced in liver tumors of SV40 T antigen transgenic mice." Cancer Res **61**(3): 1144-1153.

Terracciano, F., A. Capone, A. Montori, M. Rinzivillo, S. Partelli, F. Panzuto, E. Pilozzi, P. G. Arcidiacono, C. Sette and G. Capurso (2021). "MYC Upregulation Confers Resistance to Everolimus and Establishes Vulnerability to Cyclin-Dependent Kinase Inhibitors in Pancreatic Neuroendocrine Neoplasm Cells." Neuroendocrinology **111**(8): 739-751.

Tirosh, A., S. Mukherjee, J. Lack, S. K. Gara, S. Wang, M. M. Quezado, X. M. Keutgen, X. Wu, M. Cam, S. Kumar, D. Patel, N. Nilubol, M. V. Tyagi and E. Kebebew (2019). "Distinct genome-wide methylation patterns in sporadic and hereditary nonfunctioning pancreatic neuroendocrine tumors." Cancer **125**(8): 1247-1257.

Tran, C. G., L. C. Borbon, J. L. Mudd, E. Abusada, S. AghaAmiri, S. C. Ghosh, S. H. Vargas, G. Li, G. V. Beyer, M. McDonough, R. Li, C. H. F. Chan, S. A. Walsh, T. J. Wadas, T. O'Dorisio, M. S. O'Dorisio, R. Govindan, P. F. Cliften, A. Azhdarinia, A. M. Bellizzi, R. C. Fields, J. R. Howe and P. H. Ear (2022). "Establishment of Novel Neuroendocrine Carcinoma Patient-Derived Xenograft Models for Receptor Peptide-Targeted Therapy." Cancers (Basel) **14**(8).

Tran, C. G., A. T. Scott, G. Li, S. K. Sherman, P. H. Ear and J. R. Howe (2021). "Metastatic pancreatic neuroendocrine tumors have decreased somatostatin expression and increased Akt signaling." Surgery **169**(1): 155-161.

Umetsu, S. E., S. Kakar, O. Basturk, G. E. Kim, D. Chatterjee, K. W. Wen, G. Hale, N. Shafizadeh, S. J. Cho, J. Whitman, R. M. Gill, K. D. Jones, P. Navale, E. Bergsland, D. Klimstra and N. M. Joseph (2023). "Integrated Genomic and Clinicopathologic Approach Distinguishes Pancreatic Grade 3 Neuroendocrine Tumor From Neuroendocrine Carcinoma and Identifies a Subset With Molecular Overlap." Mod Pathol **36**(3): 100065.

Ungefroren, H., A. Künstner, H. Busch, S. Franzenburg, K. Luley, F. Viol, J. Schrader, B. Konukiewitz, U. F. Wellner, S. M. Meyhöfer, T. Keck, J. U. Marquardt and H. Lehnert (2022). "Differential Effects of Somatostatin, Octreotide, and Lanreotide on Neuroendocrine Differentiation and Proliferation in Established and Primary NET Cell Lines: Possible Crosstalk with TGF-β Signaling." Int J Mol Sci **23**(24).

Unland, R., K. Kerl, S. Schlosser, N. Farwick, T. Plagemann, B. Lechtape, S. C. Clifford, J. H. Kreth, J. Gerss, J. Mühlisch, G. H. Richter, M. Hasselblatt and M. C. Frühwald (2014). "Epigenetic repression of the dopamine receptor D4 in pediatric tumors of the central nervous system." J Neurooncol **116**(2): 237-249.

Uttenthal, L. O., M. Ghiglione, S. K. George, A. E. Bishop, J. M. Polak and S. R. Bloom (1985). "Molecular forms of glucagon-like peptide-1 in human pancreas and glucagonomas." J Clin Endocrinol Metab **61**(3): 472-479.

van Noesel, M. M., S. van Bezouw, G. S. Salomons, P. A. Voûte, R. Pieters, S. B. Baylin, J. G. Herman and R. Versteeg (2002). "Tumor-specific down-regulation of the tumor necrosis factor-related apoptosis-inducing ligand decoy receptors DcR1 and DcR2 is associated with dense promoter hypermethylation." Cancer Res **62**(7): 2157-2161.

Vandamme, T., M. Beyens, G. Boons, A. Schepers, K. Kamp, K. Biermann, P. Pauwels, W. W. De Herder, L. J. Hofland, M. Peeters, G. Van Camp and K. Op de Beeck (2019). "Hotspot DAXX, PTCH2 and CYFIP2 mutations in pancreatic neuroendocrine neoplasms." Endocrine-Related Cancer **26**(1): 1-12.

Vandamme, T., M. Beyens, M. Peeters, G. Van Camp and K. O. de Beeck (2015). "Next generation exome sequencing of pancreatic neuroendocrine tumor cell lines BON-1 and QGP-1 reveals different lineages." Cancer Genet **208**(10): 523.

Vandamme, T., M. Peeters, F. Dogan, P. Pauwels, E. Van Assche, M. Beyens, G. Mortier, G. Vandeweyer, W. de Herder, G. Van Camp, L. J. Hofland and K. Op de Beeck (2015). "Whole-exome characterization of pancreatic neuroendocrine tumor cell lines BON-1 and QGP-1." J Mol Endocrinol **54**(2): 137-147.

Vashist, Y. K., G. Uzunoglu, L. Deutsch, V. Kalinin, O. Zehler, A. Alzadjali, A. Kutup, J. R. Izbicki and E. F. Yekebas (2011). "Heme oxygenase-1 promoter polymorphism is a predictor of disease relapse in pancreatic neuroendocrine tumors." J Surg Res **166**(2): e121-127.

Vax, V. V., R. Bibi, S. Diaz-Cano, M. Gueorguiev, B. Kola, N. Borboli, B. Bressac-de Paillerets, G. J. Walker, Dedov, II, A. B. Grossman and M. Korbonits (2003). "Activating point mutations in cyclin-dependent kinase 4 are not seen in sporadic pituitary adenomas, insulinomas or Leydig cell tumours." J Endocrinol **178**(2): 301-310.

Venizelos, A., H. Elvebakken, A. Perren, O. Nikolaienko, W. Deng, I. M. B. Lothe, A. Couvelard, G. O. Hjortland, A. Sundlöv, J. Svensson, H. Garresori, C. Kersten, E. Hofsli, S. Detlefsen, M. Krogh, H. Sorbye and S. Knappskog (2021). "The molecular characteristics of high-grade gastroenteropancreatic neuroendocrine neoplasms." Endocr Relat Cancer **29**(1): 1-14.

Venizelos, A., H. Sorbye, H. Elvebakken, A. Perren, I. M. B. Lothe, A. Couvelard, G. O. Hjortland, A. Sundlöv, J. Svensson, H. Garresori, C. Kersten, E. Hofsli, S. Detlefsen, L. W. Vestermark, M. Ladekarl, E. M. Tabaksblat and S. Knappskog (2023). "Germline pathogenic variants in patients with high-grade gastroenteropancreatic neuroendocrine neoplasms." Endocr Relat Cancer **30**(10).

Vessey, S. J., P. M. Jones, S. C. Wallis, J. Schofield and S. R. Bloom (1994). "Absence of mutations in the Gs alpha and Gi2 alpha genes in sporadic parathyroid adenomas and insulinomas." Clin Sci (Lond) **87**(5): 493-497.

Vijayvergia, N., P. M. Boland, E. Handorf, K. S. Gustafson, Y. Gong, H. S. Cooper, F. Sheriff, I. Astsaturov, S. J. Cohen and P. F. Engstrom (2016). "Molecular profiling of neuroendocrine malignancies to identify prognostic and therapeutic markers: a Fox Chase Cancer Center Pilot Study." British Journal of Cancer **115**(5): 564-570.

Viol, F., B. Sipos, M. Fahl, T. S. Clauditz, T. Amin, M. Kriegs, M. Nieser, J. R. Izbicki, S. Huber, A. W. Lohse and J. Schrader (2022). "Novel preclinical gastroenteropancreatic neuroendocrine neoplasia models demonstrate the feasibility of mutation-based targeted therapy." Cell Oncol (Dordr) **45**(6): 1401-1419.

Vizziano-Cantonnet, D., A. Lasalle, S. Di Landro, C. Klopp and C. Genthon (2018). "De novo transcriptome analysis to search for sex-differentiation genes in the Siberian sturgeon." Gen Comp Endocrinol **268**: 96-109.

von Eckardstein, K., H. Gries, E. Bolik, J. Cervós-Navarro, I. N. Tschairkin and S. Patt (1997). "p53 mutation and protein alteration in 50 gliomas. Retrospective study by DNA-sequencing techniques and immunohistochemistry." Histol Histopathol **12**(3): 611-616.

Walther, B. M., I. Walther, Y. Chen and I. Petersen (2014). "GNAS1 mutation analysis in gastrointestinal tumors." Folia Histochem Cytobiol **52**(2): 90-95.

Wang, K., L. Li, J. Jin, Y. An, Z. Wang, S. Zhou, J. Zhang, B. Abuduaini, C. Cheng and N. Li (2022). "Fatty acid synthase (Fasn) inhibits the expression levels of immune response genes via alteration of alternative splicing in islet cells." J Diabetes Complications **36**(6): 108159.

Wang, P., E. Karakose, C. Argmann, H. Wang, M. Balev, R. I. Brody, H. G. Rivas, X. Liu, O. Wood, H. Liu, L. Choleva, D. Hasson, E. Bernstein, J. A. Paulo, D. K. Scott, L. Lambertini, J. A. DeCaprio and A. F. Stewart (2022). "Disrupting the DREAM complex enables proliferation of adult human pancreatic β cells." J Clin Invest **132**(15).

Wang, X., L. Jiang, O. Wallerman, S. Younis, Q. Yu, A. Klaesson, A. Tengholm, N. Welsh and L. Andersson (2019). "ZBED6 negatively regulates insulin production, neuronal differentiation, and cell aggregation in MIN6 cells." Faseb j **33**(1): 88-100.

Wang, X. C., S. Y. Xu, X. Y. Wu, H. D. Song, Y. F. Mao, H. Y. Fan, F. Yu, B. Mou, Y. Y. Gu, L. Q. Xu, X. O. Zhou, Z. Chen, J. L. Chen and R. M. Hu (2004). "Gene expression profiling in human insulinoma tissue: genes involved in the insulin secretion pathway and cloning of novel full-length cDNAs." Endocr Relat Cancer **11**(2): 295-303.

Wang, Y., Y. Chen, X. Li, W. Hu, Y. Zhang, L. Chen, M. Chen and J. Chen (2018). "Loss of expression and prognosis value of alpha-internexin in gastroenteropancreatic neuroendocrine neoplasm." BMC Cancer **18**(1): 691.

Wei, Y. L., J. Hua, X. Y. Liu, X. M. Hua, C. Sun, J. A. Bai and Q. Y. Tang (2018). "LncNEN885 inhibits epithelial-mesenchymal transition by partially regulation of Wnt/β-catenin signalling in gastroenteropancreatic neuroendocrine neoplasms." Cancer Sci **109**(10): 3139-3148.

Weisbrod, A. B., L. Zhang, M. Jain, S. Barak, M. M. Quezado and E. Kebebew (2013). "Altered PTEN, ATRX, CHGA, CHGB, and TP53 expression are associated with aggressive VHL-associated pancreatic neuroendocrine tumors." Horm Cancer **4**(3): 165-175.

Wild, A., P. Langer, A. Ramaswamy, B. Chaloupka and D. K. Bartsch (2001). "A novel insulinoma tumor suppressor gene locus on chromosome 22q with potential prognostic implications." J Clin Endocrinol Metab **86**(12): 5782-5787.

Williamson, L. M., M. Steel, J. K. Grewal, M. L. Thibodeau, E. Y. Zhao, J. M. Loree, K. C. Yang, S. M. Gorski, A. J. Mungall, K. L. Mungall, R. A. Moore, M. A. Marra, J. Laskin, D. J. Renouf, D. F. Schaeffer and S. J. M. Jones (2019). "Genomic characterization of a well-differentiated grade 3 pancreatic neuroendocrine tumor." Cold Spring Harb Mol Case Stud **5**(3).

Woischke, C., P. Jung, A. Jung, J. Kumbrink, S. Eisenlohr, C. J. Auernhammer, M. Vieth, T. Kirchner and J. Neumann (2021). "Mixed large cell neuroendocrine carcinoma and squamous cell carcinoma of the colon: detailed molecular characterisation of two cases indicates a distinct colorectal cancer entity." J Pathol Clin Res **7**(1): 75-85.

Wong, H. L., K. C. Yang, Y. Shen, E. Y. Zhao, J. M. Loree, H. F. Kennecke, S. E. Kalloger, J. M. Karasinska, H. J. Lim, A. J. Mungall, X. Feng, J. M. Davies, K. Schrader, C. Zhou, A. Karsan, S. J. M. Jones, J. Laskin, M. A. Marra, D. F. Schaeffer, S. M. Gorski and D. J. Renouf (2018). "Molecular characterization of metastatic pancreatic neuroendocrine tumors (PNETs) using whole-genome and transcriptome sequencing." Cold Spring Harb Mol Case Stud **4**(1).

Wyvekens, N., L. M. Sholl, Y. Yang, I. Tran, V. Vasudevaraja, B. C. Dickson, K. I. Al-Obaidy, N. Baniak, K. Collins, J. B. Gordetsky, M. T. Idrees, C. S. Kao, F. Maclean, A. Matoso, T. M. Ulbright, S. E. Wobker, C. D. M. Fletcher, M. S. Hirsch, J. L. Hornick, M. Snuderl and A. M. Acosta (2022). "Molecular correlates of male germ cell tumors with overgrowth of components resembling somatic malignancies." Mod Pathol **35**(12): 1966-1973.

Xiao, Z., H. Xu, J. R. Strosberg, R. Lu, X. Zhu, S. Deng, L. Ding, Q. Ni, A. L. Warshaw, X. Yu and G. Luo (2023). "EGFR is a potential therapeutic target for highly glycosylated and aggressive pancreatic neuroendocrine neoplasms." International Journal of Cancer **153**(1): 164-172.

Xie, J., T. Cai, H. Zhang, M. S. Lan and A. L. Notkins (2002). "The zinc-finger transcription factor INSM1 is expressed during embryo development and interacts with the Cbl-associated protein." Genomics **80**(1): 54-61.

Xin, Z., Y. Zhang, Z. Jiang, L. Zhao, L. Fan, Y. Wang, S. Xie, X. Shangguan, Y. Zhu, J. Pan, Q. Liu, Y. Huang, B. Dong and W. Xue (2018). "Insulinoma-associated protein 1 is a novel sensitive and specific marker for small cell carcinoma of the prostate." Hum Pathol **79**: 151-159.

Xu, G., H. Zheng and J. Y. Li (2019). "Next-generation whole exome sequencing of glioblastoma with a primitive neuronal component." Brain Tumor Pathol **36**(3): 129-134.

Xu, L., X. Li, B. Feng, Y. Ni, H. Wang and L. Wang (2010). "A novel mutation of the MEN1 gene in a Chinese kindred with multiple endocrine neoplasia type 1." Endocr J **57**(9): 839-845.

Yan, J., S. Yu, C. Jia, M. Li and J. Chen (2020). "Molecular subtyping in pancreatic neuroendocrine neoplasms: New insights into clinical, pathological unmet needs and challenges." Biochim Biophys Acta Rev Cancer **1874**(1): 188367.

Yang, J., G. W. Zhou, X. Chen, Y. Wei, C. H. Peng, G. Ning and H. W. Li (2009). "[Diagnosis and treatment of multiple endocrine neoplasia type 1 related pancreatic endocrine tumors]." Zhonghua Wai Ke Za Zhi **47**(5): 329-332.

Yu, J. H., Y. Xin, J. Eng and R. S. Yalow (1991). "Rhesus monkey gastroenteropancreatic hormones: relationship to human sequences." Regul Pept **32**(1): 39-45.

Yuan, F., M. Shi, J. Ji, H. Shi, C. Zhou, Y. Yu, B. Liu, Z. Zhu and J. Zhang (2014). "KRAS and DAXX/ATRX gene mutations are correlated with the clinicopathological features, advanced diseases, and poor prognosis in Chinese patients with pancreatic neuroendocrine tumors." Int J Biol Sci **10**(9): 957-965.

Zatelli, M. C., F. Tagliati, M. Di Ruvo, E. Castermans, L. Cavazzini, A. F. Daly, M. R. Ambrosio, A. Beckers and E. degli Uberti (2014). "Deletion of exons 1-3 of the MEN1 gene in a large Italian family causes the loss of menin expression." Fam Cancer **13**(2): 273-280.

Zhang, C., J. Zhang, G. Wang, J. Xu, Y. Li, Q. Guo, T. Zheng and Y. Zhang (2016). "Benefit of Sunitinib in the treatment of pulmonary primitive neuroectodermal tumors: a case report and literature review." Oncotarget **7**(52): 87543-87551.

Zhang, F., X. Yu, X. Wang and H. Shao (2022). "Multiple endocrine neoplasia type 1: a new germline "homozygous" variant (c.201delC) caused by detection errors." Hered Cancer Clin Pract **20**(1): 10.

Zhang, J., R. Francois, R. Iyer, M. Seshadri, M. Zajac-Kaye and S. N. Hochwald (2013). "Current understanding of the molecular biology of pancreatic neuroendocrine tumors." J Natl Cancer Inst **105**(14): 1005-1017.

Zhang, J., R. Jiang, X. Hong, H. Wu, X. Han and W. Wu (2023). "Metastatic insulinoma: exploration from clinicopathological signatures and genetic characteristics." Front Oncol **13**: 1109330.

Zhang, S., Y. Chen, S. Guo and K. N. Chen (2022). "Primary EWS/PNET of the lung with TP53 germline and SKT11 somatic mutation: A case report and review of the literature." Thorac Cancer **13**(1): 137-140.

Zhao, L., C. M. Carmean, M. Landeche, B. Chellan and R. M. Sargis (2021). "Selenomethionine modulates insulin secretion in the MIN6-K8 mouse insulinoma cell line." FEBS Lett **595**(24): 3042-3055.

Zhou, C., D. Dhall, N. N. Nissen, C. R. Chen and R. Yu (2009). "Homozygous P86S mutation of the human glucagon receptor is associated with hyperglucagonemia, alpha cell hyperplasia, and islet cell tumor." Pancreas **38**(8): 941-946.

Zhou, W., L. Gong, X. Li, Y. Wan, X. Wang, H. Li and B. Jiang (2018). "Screening key candidate genes and pathways involved in insulinoma by microarray analysis." Medicine **97**(22): e10826.

Zhou, W., X. Han, Y. Ji, D. Wang, D. Xie, Z. Qiu and W. Lou (2023). "Targeted deep sequencing reveals the genetic heterogeneity in well-differentiated pancreatic neuroendocrine tumors with liver metastasis." Hepatobiliary Surg Nutr **12**(3): 302-313.

Zhou, Y., S. Liu, C. Liu, J. Yang, Q. Lin, S. Zheng, C. Chen, Q. Zhou and R. Chen (2021). "Single-cell RNA sequencing reveals spatiotemporal heterogeneity and malignant progression in pancreatic neuroendocrine tumor." Int J Biol Sci **17**(14): 3760-3775.

Zhu, J., M. Liu, X. Liu and Z. Dong (2018). "RNA polymerase II activity revealed by GRO-seq and pNET-seq in Arabidopsis." Nat Plants **4**(12): 1112-1123.

Zurawel, R. H., C. Allen, S. Chiappa, W. Cato, J. Biegel, P. Cogen, F. de Sauvage and C. Raffel (2000). "Analysis of PTCH/SMO/SHH pathway genes in medulloblastoma." Genes Chromosomes Cancer **27**(1): 44-51.
